# Using big data for evaluating development outcomes: A systematic map

**DOI:** 10.1002/cl2.1149

**Published:** 2021-07-03

**Authors:** Francis Rathinam, Sayak Khatua, Zeba Siddiqui, Manya Malik, Pallavi Duggal, Samantha Watson, Xavier Vollenweider

**Affiliations:** ^1^ Athena Infonomics Chennai India; ^2^ 3ie New Delhi India; ^3^ Flowminder Stockholm Sweden

## Abstract

**Background:**

Policy makers need access to reliable data to monitor and evaluate the progress of development outcomes and targets such as sustainable development outcomes (SDGs). However, significant data and evidence gaps remain. Lack of resources, limited capacity within governments and logistical difficulties in collecting data are some of the reasons for the data gaps. Big data—that is digitally generated, passively produced and automatically collected—offers a great potential for answering some of the data needs. Satellite and sensors, mobile phone call detail records, online transactions and search data, and social media are some of the examples of big data. Integrating big data with the traditional household surveys and administrative data can complement data availability, quality, granularity, accuracy and frequency, and help measure development outcomes temporally and spatially in a number of new ways.The study maps different sources of big data onto development outcomes (based on SDGs) to identify current evidence base, use and the gaps. The map provides a visual overview of existing and ongoing studies. This study also discusses the risks, biases and ethical challenges in using big data for measuring and evaluating development outcomes. The study is a valuable resource for evaluators, researchers, funders, policymakers and practitioners in their effort to contributing to evidence informed policy making and in achieving the SDGs.

**Objectives:**

Identify and appraise rigorous impact evaluations (IEs), systematic reviews and the studies that have innovatively used big data to measure any development outcomes with special reference to difficult contexts

**Search Methods:**

A number of general and specialised data bases and reporsitories of organisations were searched using keywords related to big data by an information specialist.

**Selection Criteria:**

The studies were selected on basis of whether they used big data sources to measure or evaluate development outcomes.

**Data Collection and Analysis:**

Data collection was conducted using a data extraction tool and all extracted data was entered into excel and then analysed using Stata. The data analysis involved looking at trends and descriptive statistics only.

**Main Results:**

The search yielded over 17,000 records, which we then screened down to 437 studies which became the foundation of our systematic map. We found that overall, there is a sizable and rapidly growing number of measurement studies using big data but a much smaller number of IEs. We also see that the bulk of the big data sources are machine‐generated (mostly satellites) represented in the light blue. We find that satellite data was used in over 70% of the measurement studies and in over 80% of the IEs.

**Authors' Conclusions:**

This map gives us a sense that there is a lot of work being done to develop appropriate measures using big data which could subsequently be used in IEs. Information on costs, ethics, transparency is lacking in the studies and more work is needed in this area to understand the efficacies related to the use of big data. There are a number of outcomes which are not being studied using big data, either due to the lack to applicability such as education or due to lack of awareness about the new methods and data sources. The map points to a number of gaps as well as opportunities where future researchers can conduct research.

**Abbreviations**
CRD: call record details
DFID: Department for International Development
DM: data mining
DMSP‐OLS: Defense Meteorological Satellite Program‐Operational Linescan System
EC: environmental clearance
EPPI: Evidence for Policy and Practice Information
EVI: Enhanced Vegetation Index
GPS: global positioning system
IDP: internally displaced person
IE: impact evaluation
IRB: Institutional Review Board
LMICs: low and middle income countries
ML: machine learning
MNO: mobile network operator
OECD: Organisation for Economic Co‐operation and Development
RCT: randomised controlled trial
SDG: sustainable development goal
SEDAC: Socioeconomic Data and Applications Center
SR: systematic review
SRS: satellite remote sensing
SSCI: Social Sciences Citation Index

## 1 PLAIN LANGUAGE SUMMARY

### 1.1 Big data offer unrealised potential for impact evaluations (IEs)

Traditional data collection can be costly; target populations may be inaccessible, phenomena cannot always be directly observed and interviewing people may be unethical, dangerous or impossible. In addition, budget constraints can limit data collection.

“Big data” does not require data collection in the field, and can provide insight into economic, social and political behaviour.

### 1.2 What is this evidence and gap map about?

Big data consist of data such as online searches, social media, citizen reporting or crowdsourced data, process‐mediated data such as mobile phone call record details (CRD), commercial transactions data and machine‐generated data from satellites, sensors or drones.

Big data can measure outcomes that could not previously be measured using household surveys. The potential of big data to answer causal attribution, however, is still not widely understood, especially in low‐ and middle‐income countries.

**What is the aim of this evidence and gap map (EGM)?**

This EGM of studies using big data reviews methodological, ethical and practical constraints relating to the use of big data.

### 1.3 What studies are included?

The map includes IEs that use big data to evaluate development outcomes, systematic reviews (SRs) of big data IEs and other measurement studies that innovatively use big data to measure and validate any development outcomes.

Sources include social networks, internet searches, mobile data, crowd sourced data, data from public agencies, data produced by business, CRD and satellite data.

The map contains 437 studies written in English published between 2005 and 2019: 48 IEs, 381 measurement studies and eight SRs.

### 1.4 What are the main findings of this map?

There is considerable potential for measuring various development indicators using big data. The measurement studies serve as a proof‐of‐concept for evaluators wanting innovative ways to measure development outcomes.

There is potential for more IEs on development interventions. The map shows that the number of IEs that use big data to measure outcomes or control variables is growing fast, and there is scope for greater use.

Satellite data are used the most. The use of satellite data for IEs and measurement studies has been facilitated by the availability of preprocessed satellite data, new ML techniques and increased computational capacity to process the satellite images into meaningful measures of development outcomes. Despite a number of high‐profile measurement studies, CRD data has not been used to rigorously evaluate any development outcome. Similarly, other human‐sourced data and process‐mediated data have been used only sparingly in IEs.

There are potential sectors and themes where SRs will be useful. The map highlights a few potential thematic areas where SRs will be informative, most notably those of (i) all IEs that have used satellite data; and (ii) those with reference to the data sources used in rigorously evaluating forest cover.

The number of measurement studies indicates potential for more IEs in fragile contexts.

Ethical concerns and transparency issues are substantial. Ethical issues related to informed consent, data privacy, data security and unintended exclusion are severe for some of the sources of big data. Few studies report on ethical issues related to using big data.

Some capacity constraints are acute. Computational capacity is constrained and technical expertise on large‐scale big data analysis is siloed.

### 1.5 What do the findings of the map mean?

This map shows that big data can contribute to the evidence base in development sectors where evaluations are often infeasible due to data issues.

One of the key “absolute gaps” that the map has identified is that there are fewer IEs than measurement studies. Given the fast‐growing availability of big data and improving computation capacity, there is great potential for the use of big data in future IEs. However, analytical, ethical and logistical challenges may hinder the use of big data in evaluations.

This report calls for standards to be set for the reporting of data quality issues, data representativeness and data transparency, and an Institutional Review Board (IRB) review specifically designed for ethical issues related to big data.

More interaction is needed between big data analysts, remote sensing scientists and evaluators.

### 1.6 How up‐to‐date is this EGM?

The authors searched for studies published up to December 2019.

## 2 BACKGROUND

### 2.1 Introduction

#### 2.1.1 The problem, condition or issue

Policymakers need access to reliable data to evaluate development outcomes and decide on future resource allocation. Governments, multilateral organisations and other development players in low and middle income countries (LMICs) use censuses, nationally representative household surveys, other household surveys and administrative data to evaluate development programmes and policies. With the increasing complexity of development programmes, there is a need to collect a vast array of output, outcomes and contextual variables to robustly assess impact. However, significant data collection challenges remain. Data challenges for IEs include limitations on sample size and power due to budget constraints, inaccessible or difficult‐to‐reach sections of target populations, measurement errors due to recall bias, inadequate frequency and level of aggregation, inadequate information on controls and covariates, data collection lag times and difficulties in measuring long‐term impact‡ (Wassenich, 2007). Further, in some contexts, like conflicts and humanitarian emergency situations, data collection is often impossible. The data gaps and challenges are particularly significant for the populations and countries where the need for evidence‐informed policy decisions are perhaps the greatest (Gaarder & Annan, 2013). Another key shortcoming of survey data are inadequate aggregation at subnational administrative units such as districts, counties or villages, inhibiting evaluation of programmes with spatial attributes.

#### 2.1.2 Data sources

Big data offers great potential for answering some of these data needs. More importantly, it answers the causal questions around which policies or interventions work, including in contexts where traditional methods of data collection are challenging. The UN Global Pulse (2013) defines big data as being digitally generated (as opposed to digitised manually), passively produced (a by‐product of digital services, transactions and interactions), automatically collected and geographically and temporally trackable. Although there is no formal definition for big data, currently the term is characterised by the three Vs: high volume, velocity and variety. Satellite images, sensors and drones, mobile phone CRDs, commercial transactions data, online searches, social media, citizen reporting or crowdsourced data are the sources of big data.

Integrating big data with traditional household surveys and administrative data can complement data availability, quality, granularity, accuracy and frequency, as well as help measure development outcomes temporally and spatially in a number of new ways (BenYishay et al., 2018; Lokanathan et al., 2017; Salganik, 2017; UN Global Pulse, 2016; York & Bamberger, 2020). For example, satellite images and mobile CRD have been used in mapping poverty (Blumenstock et al., 2015; Jean et al., 2016), disaster response (Lu et al., 2012; Wilson et al., 2016) and food security (Decuyper et al., 2014). Web searches and social media were used in predicting unemployment and crime instances (Gerber, 2014; Xu et al., 2013).

While big data is increasingly used for tracking indicators and monitoring development progress on SDGs (Lokanathan et al., 2017; UN Global Pulse, 2012; Vaitla, 2014), available data are less often utilised to address causal questions about the effects of specific policies and programmes. Big data can contribute to answering some of the causal questions around which interventions work. Big data prediction models can generate proxy estimates for key development outcomes such as wealth, human development, infrastructure quality, forest cover and more, which can be used in experimental (Jayachandran et al., 2016; Pellegrini, 2019) and quasi‐experimental studies (BenYishay et al., 2018; Jaiswal et al., 2020). Satellite images such as night light, crop intensity, water availability, land use, proximity to services and physical attributes such as elevation or slope can be used in IEs as a direct measure of outcomes or as covariates. Furthermore, big data can be used for measuring and evaluating the long‐term impacts of policies and programmes, conducting ex‐post evaluations and estimating spatial heterogeneity. For example, satellite data are available at least as far back as 1993 for all places (high‐resolution pictures are available for the entire globe at a granularity as low as 1 × 1 metre), allowing measurement of long‐term impacts. This can help fill the gaps in evidence that cannot be addressed by traditional data sources.

#### 2.1.3 Why it is important to develop the systematic map

The potential of big data to answer causal attribution, however, is still not widely understood or used, especially in LMICs (York & Bamberger, 2020). In this context, a systematic collection of various sources of big data and ways of measuring and evaluating development outcomes will be a great value addition to the development community's contribution to evidence‐informed policymaking.

In this paper we look at IEs, SRs and measurement studies§ that use big data to evaluate development outcomes with a special focus on fragile contexts. The study highlights the new sources of data; how these new data sources can be used for measuring development outcomes innovatively; and how these new measures can be used in IEs. We map different sources of big data onto development outcomes based on SDGs to identify the current evidence base and its gaps.

## 3 OBJECTIVES

The overarching aim of this report is to inform policymakers and evaluators of existing evaluations based on big data and to provide a database of big data‐based IEs and studies that could inform future IEs. Specifically, the objectives of the research are to:

- Identify rigorous IEs, SRs and the studies that have innovatively used big data to measure any development outcomes, with special reference to fragile contexts;
- Summarise current understanding of potential uses, pros and cons, reliability, biases, risks and ethical issues in using big data for measurement and evaluation of development outcomes and
- Generate interest and awareness among key stakeholders (evaluators, researchers, donors, practitioners, implementers and policymakers) of the potential as well as challenges of using big data.

This systematic map addresses the following questions:

- How have different types of big data and methods been used for measuring and evaluating development outcomes?
- How dispersed or concentrated is the use of big data across development goals and geographies?
- What are the potential biases, measurement reliability issues, pros and cons, risks and ethical issues in using big data for measuring and evaluating development outcomes?
- What are some of the unexplored but promising applications of big data for IEs?

## 4 METHODS

### 4.1 Evidence and gap map: Definition and purpose

We follow 3ie's methodology and process for evidence gap maps (Snilstveit et al., 2017). To create this map, we used systematic methods to identify any completed and ongoing IEs, SRs and big data measurement studies relevant to our research objectives. We conducted systematic searches and data extraction as described in Appendices C and D. The studies identified are mapped on to the framework of big data sources and SDG outcomes to provide a visual display of the volume of and the trends in the evidence base. We also coded how the included studies have dealt with ethical and transparency related challenges. The systematic map is available through an online interactive platform on the 3ie website and allows users to explore the available evidence through different filtering options.** There are links to study summaries in the 3ie repositories (wherever applicable) and confidence ratings for the SRs.

### 4.2 Framework development and scope

For the purpose of this research, we define big data sources as digitally generated, passively produced and automatically collected data, as defined in UN Global Pulse (2013). The sources of big data include satellite images, sensors and drones, mobile phone CRDs, commercial transactions data, online searches, social media, citizen reporting or crowdsourced data. See Table 2 for more details on various sources of big data adapted from UN Global Pulse (2012, 2013), Yeung & Fok (2014) and Blazquez and Domenech (2018).

**Table 1:** Selection criteria for studies

Category	Description
Population	This map includes all population from all countries but we provide breakdowns for rural areas, urban areas, conflicted‐affected persons and ethnic minorities. We also provide breakdowns for LMICs and fragile contexts separately
Sources of big data	Big data may originate from any of the following sources:
	Human‐sourced information: a. Social networks b. Internet searches c. Mobile data content d. Citizen reporting or crowdsourced data Process‐mediated data (traditional business systems and websites): a. Data produced by public agencies b. Data produced by businesses c. Mobile phone CRD Machine‐generated data (automated systems): a. Data from fixed sensors b. Data from mobile sensors (tracking) c. Data from satellites
Outcomes	1. Economic development and livelihoods 2. Agriculture and food security 3. Health and well‐being 4. Quality of education 5. Governance and human rights 6. Water and sanitation 7. Energy, industry and infrastructure provision 8. Urban development 9. Environmental sustainability 10. Partnerships for goals
Study design	IEs:
	Randomised controlled trial Regression discontinuity design Controlled before‐and‐after study using appropriate methods to control for selection bias and confounding, such as propensity score matching or other matching methods Instrumental variable estimation or other methods using an instrumental variable such as the Heckman two‐step approach Difference‐in‐differences A fixed‐effects or random‐effects model with an interaction term between time and intervention for baseline and follow‐up observations Natural experiments Other quasi‐experimental studies inducing synthetic control studies Survey, laboratory or lab‐in‐the‐field type experiments Cross‐sectional or panel studies with an intervention and comparison group using methods to control for selection bias and confounding as described above
	Measurement studies
	We included the studies that innovatively used big data to measure and validate any development outcomes. These studies use big data to measure components that would have been difficult to measure using survey data.
	SRs
	We include only the reviews that specifically looked at studies that used big data to measure development outcomes and explicitly described the search, data collection and synthesis methods according to a standard SR protocol, such as the 3ie SR protocol.

Abbreviations: CRD, call record detail; IE, impact evaluation; SR, systematic review.

**Table 2:** Sources of big data

Data type	Source of data
Human‐sourced information	
Social networks	Text, metadata and location data from social networking sites, opinion platforms, blogs, pictures, videos, and so forth, such as Twitter, Facebook, LinkedIn, YouTube, Wiki pages
Internet searches	Internet text and internet search queries, ie Google Trends; web logs (open data)
Mobile data content	Text messages
Citizen reporting or crowdsourced data	OpenStreetMap, Humanitarian Data Exchange platform, and so forth, Collected from a large group of people who voluntarily provide information. This is a useful source for recording events and receiving people's opinion and feedback on issues. Collected through hotlines, user‐generated maps, marking of instances, events, and so forth
Process‐mediated data	
Data produced by public agencies	Medical records; postal data; tax data, and so forth
Data produced by businesses	E‐commerce transaction records and credit card transaction records, ATM transactions, mobile money transfers, savings and loan repayments (collected by the service provider as a part of regular business operation and monitoring; proprietary and commercially sensitive data)
	Tolls and public transport card use data
Mobile phone CRD	Mobile CRDs that provide metadata on when the call took place, the cost, the time and the recipient of the call; location of the caller and of the recipient; and the users' mobility, social interaction and airtime transaction details; top‐up data
Machine‐generated data	
Data from fixed sensors	Home automation, weather/pollution sensors, traffic sensors/webcams, security/surveillance videos/images and activity records such as electricity metres (mostly administrative data collected by the authorities as a part of regular monitoring)
Data from mobile sensors (tracking)	Community or privately owned drones (common property or privately held data)
	Mobile phone GPS (open data available from Google)
	Vehicle GPS and self‐tracking apps (proprietary data)
Data from satellites	Open data available from a number of sources, for example:
	Defense Meteorological Satellite Program‐Operational Linescan System Visible Infraref Imaging Radiometers Landsat European Space Agency Land Cover, and so forth

Abbreviation: GPS, global positioning system.

3ie evidence gap maps compile IEs and SRs. However, in this study, we include IEs and SRs as well as measurement studies: the studies that have innovatively used big data to measure and validate any development outcome. These are multidisciplinary studies that use state‐of‐the‐art methods from computer science and statistics to collect, clean and analyse big data for measuring development outcomes. For example, Jean et al. (2016) use transfer learning techniques as well as daytime and night light data from satellite images to estimate consumption expenditure at the cluster (village) level to map poverty in five African countries: Nigeria, Tanzania, Uganda, Malawi and Rwanda. While a number of such studies have used big data for measuring various development outcomes, few IEs have used these innovative big data‐based outcome measures. These measurement studies, we hope, would serve as proofs of concept for innovative use of ML and big data that can be used in future evaluations.

3ie defines an IE as a “study of the attribution of changes in the outcome to the intervention.”†† For the purpose of this systematic map, we define big data‐based IEs as any experimental or quasi‐experimental studies that use any form of big data to measure the outcomes of interest and/or the confounding variables.

We use the Organisation for Economic Co‐operation and Development (OECD) definition of fragile contexts, which includes conflicts, institutional fragility, social fragility, environmental risks, health risks and climatic risks. This list is more inclusive than the list used by the UK Department for International Development (DFID) and the World Bank, which includes conflict and institutional and social fragility (DFID, 2016). See Appendix A for more details on the classifications and country list. We use the OECD definition for classifying fragile contexts based on:

- Difficult terrain
- Natural disasters
- Conflict or humanitarian crisis
- Chemical or radio‐nuclear issues
- Disease outbreaks or epidemics.

Using big data in evaluation poses a number of analytical challenges on issues including data quality, transparency, generalisability, and privacy and ethical challenges such as consent for using data and anonymisation of the data. This report also explores how the included studies dealt with these challenges.

#### 4.2.1 Types of study design

Table 1 summarises the criteria we used for searching, screening and including the studies for the map.

### 4.3 Search methods and sources

Innovations in the type of devices available for measurement (satellites and sensors); daily personal use (mobiles, wearables, Internet of Things, etc.); social interaction (blogs, Facebook, Twitter, WhatsApp, etc.); and recording business transactions digitally (CRD, e‐transactions, mobile money, credit card payment, etc.) have led to an explosion of automatically collected data. However, there is no official definition of big data. McKinsey defined it broadly as data “whose size is beyond the ability of typical database software tools to capture, store, manage, and analyze” (Manyika et al., 2011).

UN Global Pulse (2013) defines big data for the purposes of development as being digitally generated (as opposed to digitised manually), passively produced (a by‐product of digital services, transactions and interactions), automatically collected and geographically or temporally trackable. While the size, velocity and veracity are all defining characteristics of big data, the definition relevant for IE is that these are nonsampled data, passively left behind by humans using digital devices and services or automatically collected by the services providers for the purpose other than statistical inference (Letouzé, 2016; UN Global Pulse, 2016). Hence, unlike the conventional survey data where the respondents say what they do or feel, big data captures what people actually do. The implication of this is that big data is nonreactive: in other words, there is less likelihood of social desirability bias (Salganik, 2017). The other key characteristic of big data that matters for evaluation is that it is near real‐time and can be available across multiple frequencies (e.g., hourly, daily) over a long period. Table 2 provides the types of big data, subclassifications, definitions and sources.

Using these definitions, we include the following broad classification of big data‡‡:

- Human‐sourced information from social networks that is provided voluntarily by users;
- Process‐mediated data from traditional business systems and websites that includes digitally recorded business activities;
- Machine‐generated data from automated systems includes information from sensors and machines that measure and record events and situations in the physical world.

### 4.4 Analysis and presentation

#### 4.4.1 Report structure

We use the SDGs as the basis for identifying the outcome categories, similar to Phillips et al. (2017).§§ We have regrouped them into smaller thematic groups based on complementarities between the sectors. Table 3 provides the definition of each of the outcomes as defined in UN (2019). Appendix B provide more details on the submaps where further breakdowns have been provided for key subclassifications where relevant.

**Table 3:** Outcome categories and definitions

Category	Definition
Economic development and livelihoods (SDGs 1 and 8)	End poverty in all its forms everywhere
	Promote sustained, inclusive and sustainable economic growth, full and productive employment and decent work for all
Agriculture and food security (SDG 2)	End hunger, achieve food security and improved nutrition and promote sustainable agriculture
Health and well‐being (SDG 3)	Ensure healthy lives and promote well‐being for all ages
Quality of education (SDG 4)	Ensure inclusive and equitable quality education and promote life‐long learning opportunities for all
Governance and human rights (SDGs 5, 10 and 16)	Achieve gender equality and empower all women and girls
	Reduce inequality within and among countries
	Promote peaceful and inclusive societies for sustainable development, provide access to justice for all and build effective, accountable and inclusive institutions at all levels
Water and sanitation (SDG 6)	Ensure availability and sustainable management of water and sanitation for all
Energy, industry and infrastructure provision (SDGs 7 and 9)	Ensure access to affordable reliable, sustainable, and modern energy for all
	Build resilient infrastructure promote inclusive and sustainable industrialisation and foster innovation
Urban development (SDG 11)	Make cities and human settlements inclusive, safe, resilient and sustainable
Environmental sustainability (SDGs 12, 13, 14 and 15)	Ensure sustainable consumption and production patterns
	Take urgent action to combat climate change and its impacts
	Conserve and sustainable use the oceans, seas and marine resources for sustainable development
	Protect, restore and promote sustainable use of terrestrial ecosystems, sustainably manage forests, combat desertification, and halt and reverse land degradation and halt biodiversity loss
Partnerships for goals (SDG 17)	Strengthen the means of implementation and revitalise the global partnership for sustainable development

Abbreviation: SDG, sustainable development goal.

#### 4.4.2 Filters for presentation

We have added filters such as population, challenging contexts, geographic coverage to provide more details about the data sources and outcomes of interest. The population filter provides information on whether the studies in the map identify subgroups such as rural, urban, refugees, conflict affected persons, ethnic minorities. With the challenging contexts filter we show if the studies were in areas which were remote, conflict affected, affected by disease outbreak, and so on. We also identified different regions where the studies were conducted.

#### 4.4.3 Dependency

Each unit of analysis was a report, peer reviewed journal publication, working paper. We included one study per unit of analysis. In cases where we had multiple reports we included the latest one with preference given to peer review versions and/or based on ease of access to a particular version. When multiple studies were covered by a single report, we included the report and then mapped the data sources and outcomes accordingly.

### 4.5 Data collection and analysis

#### 4.5.1 Screening and study selection

We first employed Evidence for Policy and Practice Information (EPPI)‐Reviewer's built‐in text mining, an ML technique, to sort the studies based on the inclusion and exclusion criteria at the title and screening stage (see Appendix D for more details on how text mining was used for screening). This reduced the number of studies to be screened at the title and abstract level to 9720. At the second stage, three researchers screened the studies for eligibility based on inclusion and exclusion criteria defined in the study protocol. At the end of stage two, we had identified 1348 studies to be screened at the full‐text level.

At stage three, four researchers coded the studies at the full‐text level. We followed a single screening with safety first approach,*** where only one reviewer screened each record and coded doubtful studies as “to discuss” to avoid excluding the studies that are potential includes. About 25% of the studies at this stage were double‐coded for consistency and any disagreements were resolved with the lead author. About 20% of the studies were spot‐checked by the lead author. At the full‐text screening stage, we excluded 1292 studies. We excluded 251 studies based on irrelevant data sources, 408 studies on outcomes and 633 studies on study designs. Studies excluded on design were primarily policy papers or computer science technical research papers that aimed at developing new algorithms rather than measuring development outcomes.

#### 4.5.2 Data extraction and management

The three‐stage process of screening resulted in a final list of 437 studies including 48 IEs, 381 measurement studies and 8 SRs. Through a consultation process, we identified the metadata to be extracted using a standardised data extraction tool and defined them in the study protocol. During the final studies coding, we collected metadata such as outcomes studies, outcome subcategories, data sources used, geographical location of the intervention, country, evaluation design, target population, data transparency, ethics and other bibliographic information from the included studies (see Appendix D for the data extraction tool). We also critically appraised the SRs (see Appendix E for the SR appraisal tool and summary of the included SRs). Due to the size and the nature of included studies we did not conduct critical appraisal of IEs or measurement studies.

#### 4.5.3 Tools for assessing risk of bias/study quality of included reviews

All SRs have been critically appraised using 3ie's critical appraisal checklist, adopted from the SURE checklist. Appendix F summarises all the SRs included in our sample and Table G1 provides details on the critical appraisal of the included studies.

#### 4.5.4 Methods for mapping

The studies were mapped using 3ie's evidence gap map platform, which is organised into rows and columns. Various big data sources are placed in the rows and the development themes are placed in the columns. Any intersecting cell represents the development outcome measured or evaluated using the particular type of big data. Different colour bubbles represent the type of study: grey bubbles denote IEs, blue bubbles denote measurement studies, green bubbles denote high‐quality SRs and red bubbles denote low‐quality SRs. Hovering over the bubbles will show the links to studies. There are also filters for different regions, countries, study design, fragile context and the target population of the studies.

Development themes (such as environmental sustainability, economic development and livelihoods) contain a large number of studies. We have provided maps within the main map to show how the studies are distributed across the subthemes under these broad themes. In the submaps, the big data sources are mapped against level 2 or level 1 subclassification provided in the SDGs as relevant. For example, the submap for economic development and livelihoods has been coded against the level 2 indicators of eradicating poverty (SDG 1) and employment and economic growth (SDG 8), and the submap for environmental sustainability is coded against the level 1 classification of SDGs 12, 13, 14 and 15. The following development themes have submaps:

- Economic development and livelihoods
- Health and well‐being
- Governance and human rights
- Urban development
- Environmental sustainability.

## 5 RESULTS

### 5.1 Description of studies

#### 5.1.1 Results of the search

**Figure 1:**
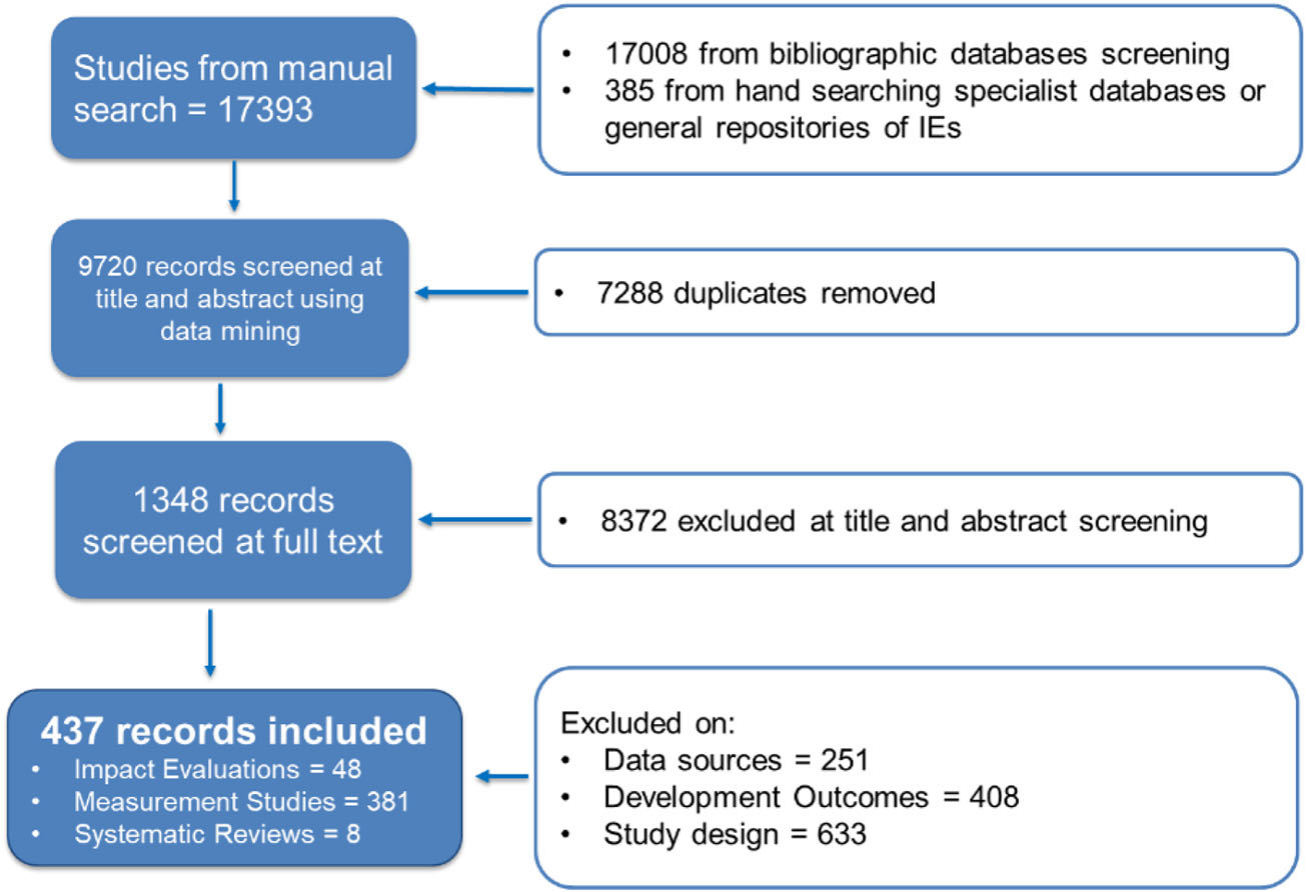
The PRISMA flowchart. *Source*: Author's own calculation

We systematically searched academic databases with the help of an information specialist (see Appendix C for the list of academic databases and search strings) and manually searched specialist organisational websites and grey literature sources. The initial searches resulted in 17,393 studies, of which 17,008 studies were identified through bibliographic databases search and 385 studies were identified through hand‐searching specialist databases and IE and grey literature repositories (Figure 1). All the results were uploaded onto EPPI‐Reviewer 4 for screening and coding. Screening of the studies was done in three stages.

#### 5.1.2 Excluded studies

This report includes papers written in English and published between 2005 and 2019. This map does not include studies that develop algorithms or methodologies for using big data without explicit application to measuring or evaluating a development outcome. This map does not include studies that describe how big data and ML have been used in development programming to help programme implementation, coordination and management for designing and scaling new development solutions; neither does it include studies that show how big data methods are used in randomised controlled trials (RCTs) to identify the differential impact of subgroups and in improving survey data collection, such as defining sampling frames.

#### 5.1.3 Studies awaiting classification (if applicable)

If applicable, list the characteristics of any studies that have been identified as potentially eligible but have not been incorporated into the map (ER40).

### 5.2 Synthesis of included studies

The map contains 437 studies, of which 48 are IEs, 381 are measurement studies and 8 are SRs. Of the 48 IEs, 8 are RCTs and the remaining are quasi‐experimental studies.

**Figure 2:**
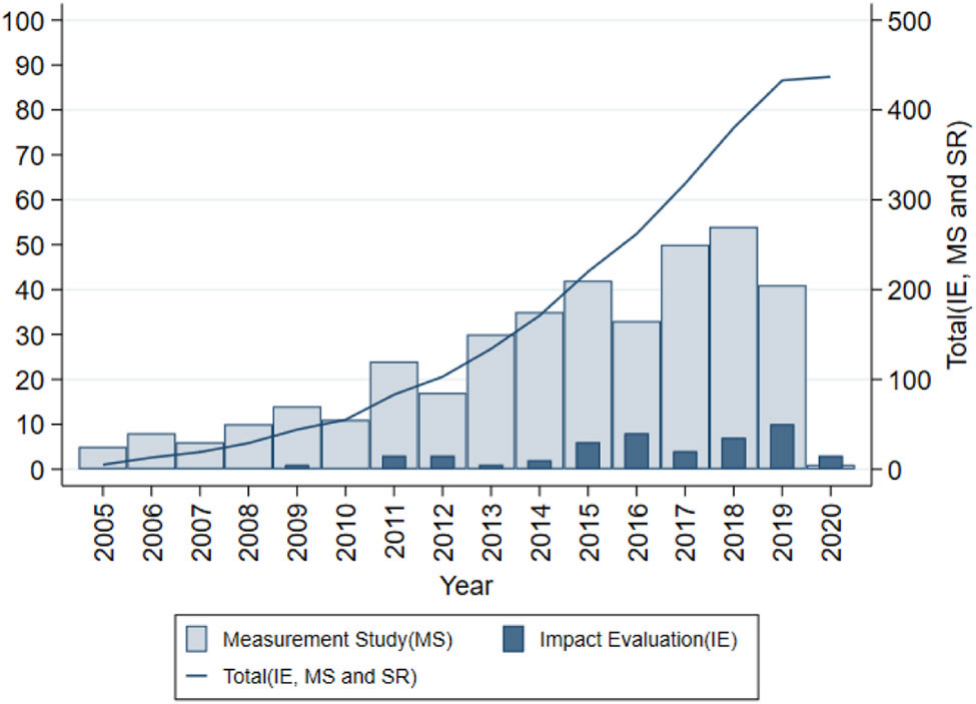
Number of studies published per year. *Source*: Authors' own calculation

Figure 2 displays the number of studies published each year from 2005 to 2020. The light blue bar shows the measurement studies, the dark blue bar shows IEs and the line indicates the cumulative number of measurement studies, IEs and SRs. The number of measurement studies has grown gradually from 2005 to 2012, increasing substantially every year since then with maximum numbers in 2017 and 2018. The past five years alone have accounted for more than 60% of the studies, indicating the increasing availability of big data, improved computational capacity and greater interest among researchers and journals.

The figure also shows that applying big data to IEs is a new phenomenon. The first IE using big data was published in 2009. While almost all the IEs were published after 2013, more than three‐quarters of the IEs were published in the last five years. We expect that the measurement studies will be proofs of concept, leading IEs to adopt to these approaches to innovatively measure development outcomes in evaluations. The map also points to the gap between the growth of measurement studies and use of big data in IEs.

**Figure 3:**
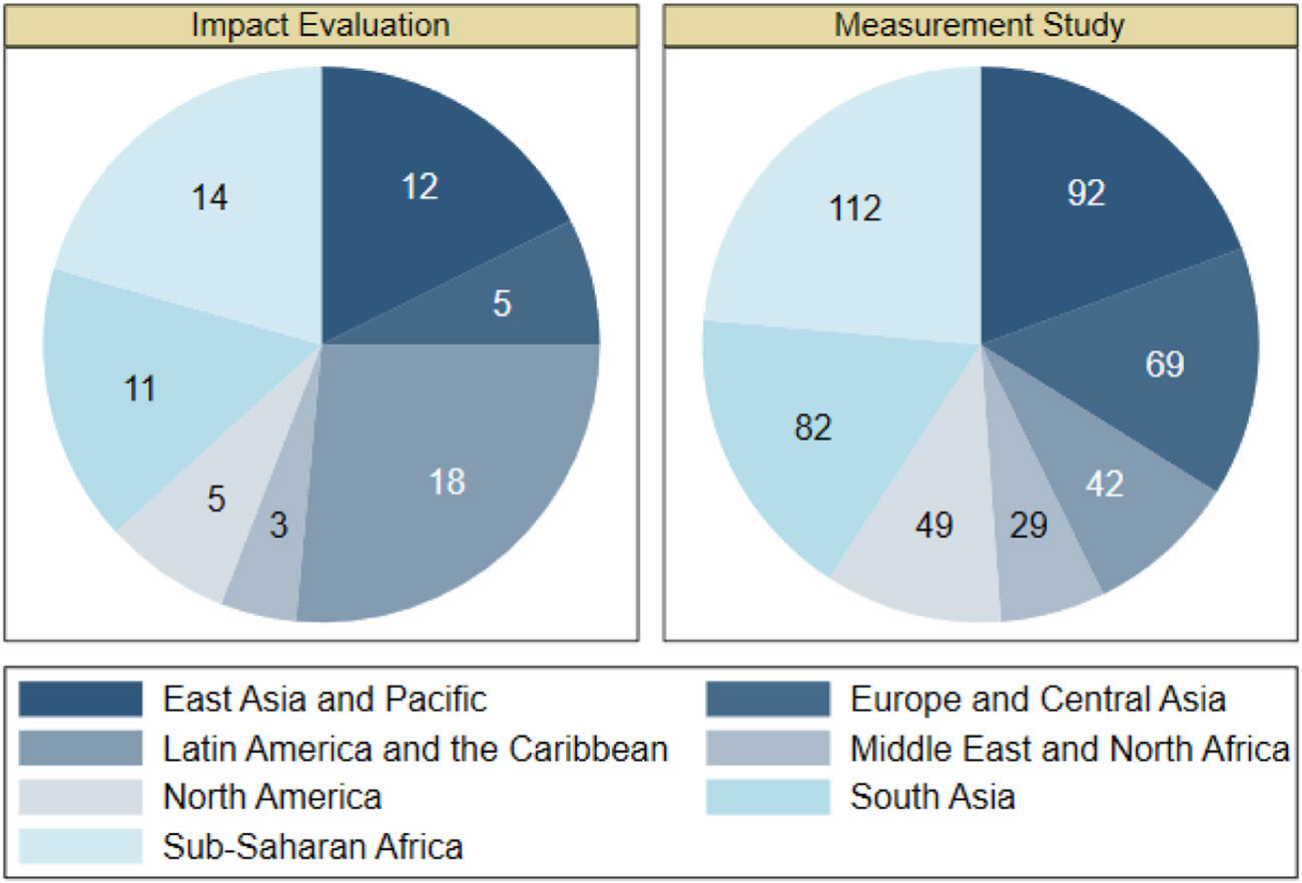
Distribution of studies over regions. *Source*: Authors' own calculation

Figure 3 shows the geographical distribution of the included studies. About 50% of the studies (*n* = 210) are from Asia and close to 30% (*n* = 132) are from Sub‐Saharan Africa. The distribution of IEs and measurement studies are roughly similar to the overall distribution. One notable exception is Latin America and the Caribbean where the region accounts for 15% of total studies (*n* = 65), but substantially more IEs (38%, *n* = 18).

**Figure 4:**
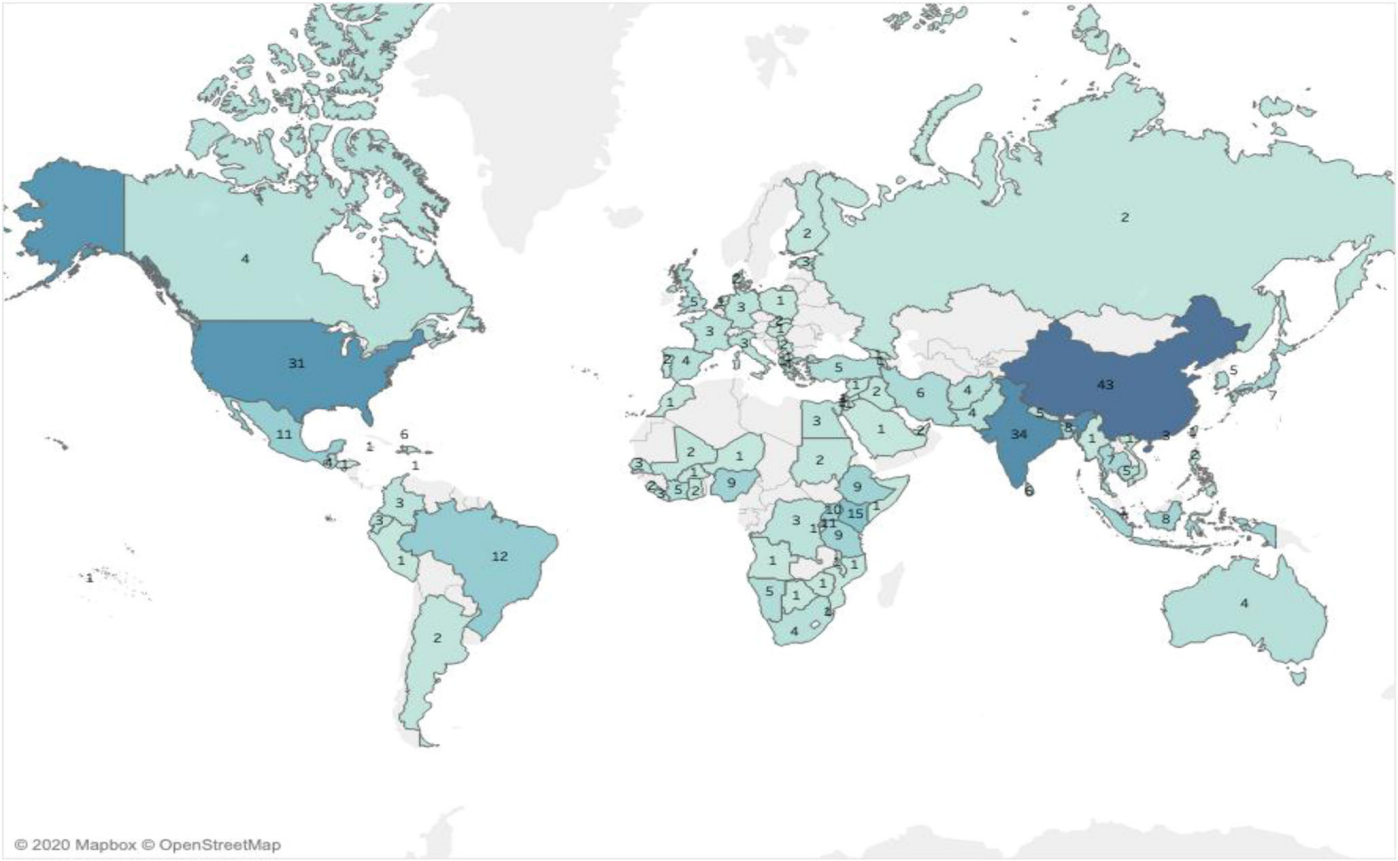
Geographical distribution of studies. *Source*: Authors' own calculation

China (43) and India (34) are the most‐studied countries, and Kenya has highest number of studies (15) in Sub‐Saharan Africa (Figure 4). While East Africa is very well‐represented on the map, other African countries have fewer entries and several countries in West and North Africa have no studies at all. About 35 of the studies are multi‐country studies and 11 studies did not specify the country name primarily to conceal the identity of the data provider. The distribution of IEs and measurement studies are again roughly similar to the overall distribution but Latin America countries, particularly Mexico (5), account for a higher proportion of IEs. See Table H1 for a list of top 20 countries with the maximum number of studies and Table H2 for the geographical distribution of studies across the regions in Appendix G.

**Figure 5:**
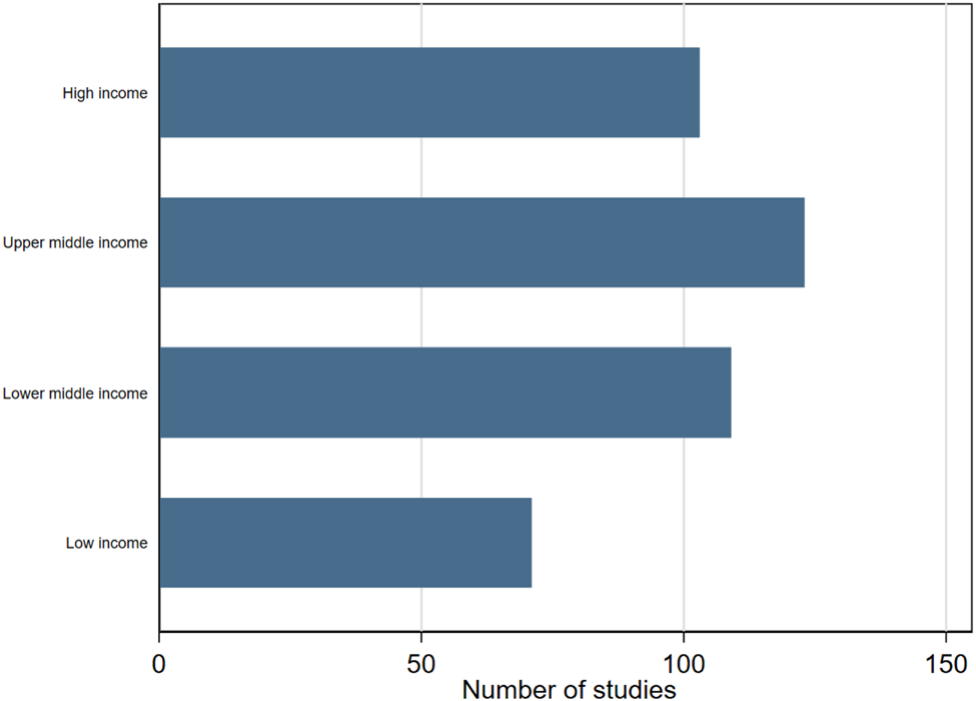
Number of studies by income classification. *Source*: Authors' own calculation

We find that most of the studies are concentrated in middle income countries. There are 232 studies (53%) in the middle income group, followed by 103 studies (24%) in the high income group and 71 studies (16%) in the low income group (Figure 5). Overall, about 69% (*n* = 303) of the total studies are from LMICs, but the IEs are distributed more in favour the LMICs as 83% of them (*n* = 40) are from LMICs. One of the notable features of the studies on the map is that about 82% (*n* = 359) of the total studies are published in peer‐reviewed journals and the remainder are working papers (18%, *n* = 78).

#### 5.2.1 Distribution of studies across data sources

**Figure 6:**
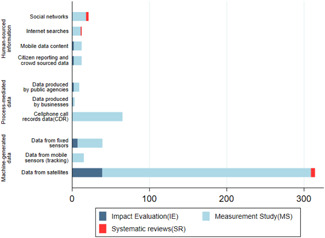
Number of studies per different type of big data. *Source*: Authors' own calculation

As discussed in Section 2.1.2, big data can be generated by human interaction on social media, process‐mediated data recorded by governments and the business and machine‐generated data that is recorded by the automated systems. Figure 6 shows that machine‐generated data are used the most. Of the total number of studies, close to 84% (*n* = 380) of the studies used some form of machine‐generated data, while 12% (*n* = 53) of the studies used human‐generated sources and 17% of the studies (*n* = 77) used process‐mediated data.

Table 4 below provides a detailed breakdown of the number of studies per data source.

**Table 4:** Number of IEs and measurement studies across data sources

Data source	IEs	Measurement studies	SRs
Human‐sourced			
Social networks	0	18	3
Internet searches	0	11	1
Mobile data content	2	10	0
Citizen reporting or crowdsourced data	2	10	0
Process‐mediated			
Data produced by public agencies	1	2	0
Data produced by businesses	2	7	0
Mobile phone CRD	0	65	0
Machine‐generated data			
Data from fixed sensors	7	32	0
Data from mobile sensors (tracking)	0	15	0
Data from satellites	39	270	5
Total studies	48	381	

*Note*: Percentage of subcategory total in parentheses. Columns do not add up due to multiple entries.

##### Data from satellites and fixed sensors

Satellite data are the most used source of big data as it accounts for 71% of the measurement studies (*n* = 210) and close to 81% of the IEs (*n* = 39). Data from fixed sensors (such as weather and pollution sensors, traffic sensors and electricity metres that provide high‐frequency, localised measurements) could also be readily used in IEs. This is the second most used data source, with 15% of the IEs using these sources. This shows that the data from satellites and in situ sensors that help measure spatial outcomes are used most in IEs. Other big data sources have been seldom used for IEs despite measurement studies showing proof‐of‐concept.

##### Mobile phone CRD

A good number of measurement studies have used CRDs (17% *n* = 65) for measuring population movement, migration, disease spread and even to understand the literacy level of the subscribers. Surprisingly, we found no IEs that used this source of big data despite the availability of a good number of proof‐of‐concept papers in measuring key development outcomes.

##### Human‐sourced data

Social networks including Facebook, Twitter and Wiki pages were used to measure development outcomes in 18 measurement studies. Internet searches like Google trends and other search engine queries were used in 11 studies. Mobile data content and crowdsourced data were used in 10 studies, primarily to measure disease outbreak, price data or opinion on issues like development services. This source has not been used in IEs, with two notable exceptions using crowdsourced data (Edjekumhene et al., 2019; Van der Windt & Humphreys, 2016).

##### Complementarity between data sources

There are about 57 studies on the map that have combined at least two sources of data and about seven of them have combined three or more sources (Table H3 in Appendix G). Data from fixed sensors and satellites data seem to complement each other well: 13 measurement studies and 2 IEs have combined these two sources. Mobile phone CRD and satellite data are the other combination that has been used repeatedly. About 10 of the measurement studies have combined CRD data and satellite data in their analysis (see Section 5 for a discussion and example on how satellite data and CRD can be combined together for better results). However, IEs seem not to have exploited this complementarity. See Table H3 in Appendix G for a list of studies using multiple sources of big data.

#### 5.2.2 Distribution of studies across development themes

**Figure 7:**
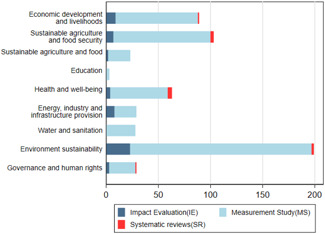
Number of studies against development outcomes. *Source*: Authors' own calculation

Section 4.2 identifies 10 broad development themes based on SDGs. Figure 7 and Table 5 show the number of studies across the development themes. About 50% of studies (*n* = 217) focus on environmental sustainability, which includes sustainable consumption and production, climate change, underwater life, and life on land. Economic development and livelihoods accounts for about 26% of the total studies (*n* = 114). Urban development and health account for 16% each (*n* = 68). Governance and human rights (7%, *n* = 30) and energy, industry and infrastructure (7%, *n* = 30) account for the remaining studies.

**Table 5:** Distribution of studies across development themes

Development themes	IEs	Measurement studies	SRs	Total
Economic development and livelihood	8	105	1	114
Sustainable agriculture and food security	2	21	0	23
Health and well‐being	5	58	5	68
Education	0	4	0	0
Water and sanitation	0	28	0	28
Governance and human rights	3	26	1	30
Energy, industry and infrastructure	8	22	0	30
Urban development	3	69	3	75
Environment sustainability	25	190	2	217
Global partnership	0	2	0	2

While the distribution of IEs and measurement studies across the development themes remains the same as the overall distribution, there are a substantial number of IEs on economic development and livelihoods (17%) and energy, industry and infrastructure (17%). While most of the SRs looked at cross‐sectoral themes, health is the most‐studied sector (*n* = 5), followed by urban development (*n* = 3) and environment sustainability and economic development (*n* = 2). See Appendix F for critical appraisal of the SRs.

#### 5.2.3 Units of observation

**Figure 8:**
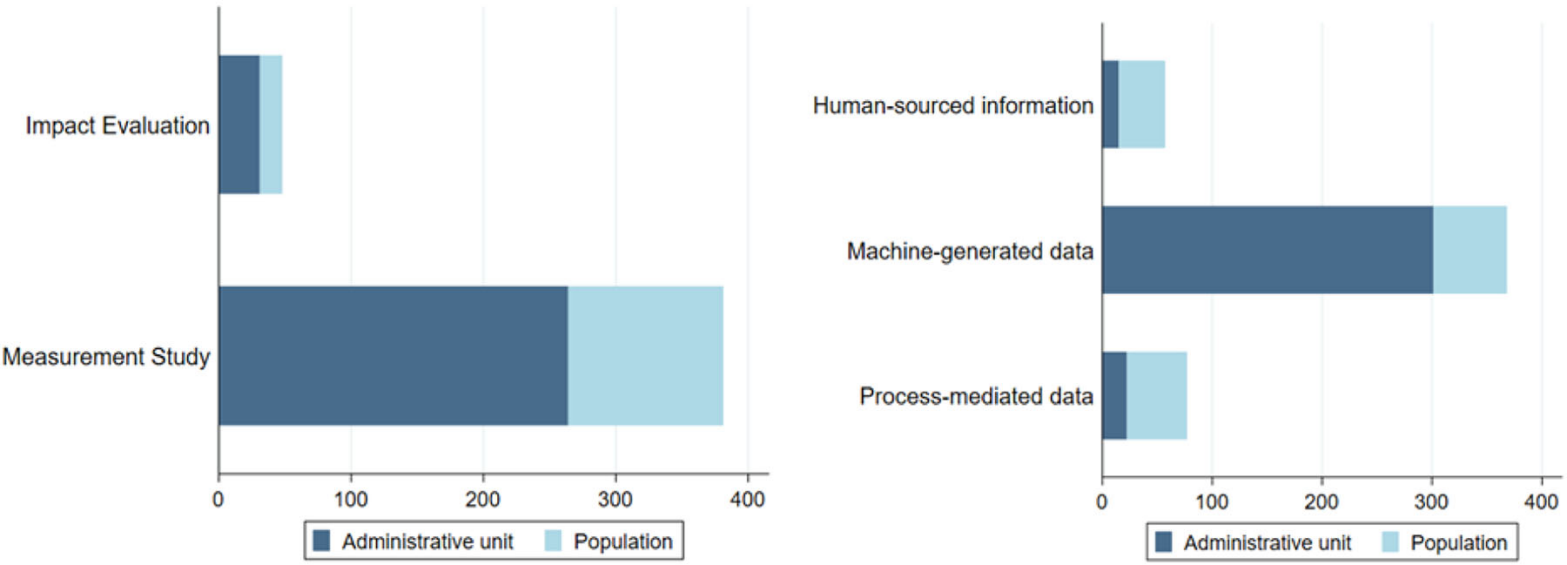
Units of observation. *Source*: Authors' own calculation

The unit of observation (or unit of analysis) is the class of elemental unit that constitutes the population and the units of measurement. Typically, in IEs, the units of observation are individuals, households, facilities (in facility surveys) or various level of administrative units such as villages, counties or districts. We have classified the unit of observation as population (including both individuals and households) or administrative units (villages, land parcels or any other units with a spatial element). The unit of observation seems to be an important element in analysing the use of big data in measuring development outcomes. Figure 8 (Panel 1) shows that about 70% of the measurement studies (*n* = 267) and 65% of the IEs (*n* = 31) have administrative units as their unit of observation. There is a clear distinction between different sources of big data, as shown in Figure 8, Panel 2. The unit of analysis for satellite data‐based studies is predominantly administrative units (*n* = 259, 83%), while CRD‐based studies are usually based on population units (*n* = 53, 82%). This difference shows that satellite data are more applicable when the outcome of interest has some spatial dimension such as local economic development, agricultural land productivity, forest cover or urban development.

#### 5.2.4 Studies using mixed methods

Mixed‐methods IEs that combine qualitative and quantitative analyses help assess the quality implementation and reliability of data and understand the mechanism of programme impact (Bamberger, 2012). Big data IEs can be combined with qualitative methods. However, only three IEs and five measurement studies reported using mixed methods.

#### 5.2.5 Studies with a rural or urban focus

**Figure 9:**
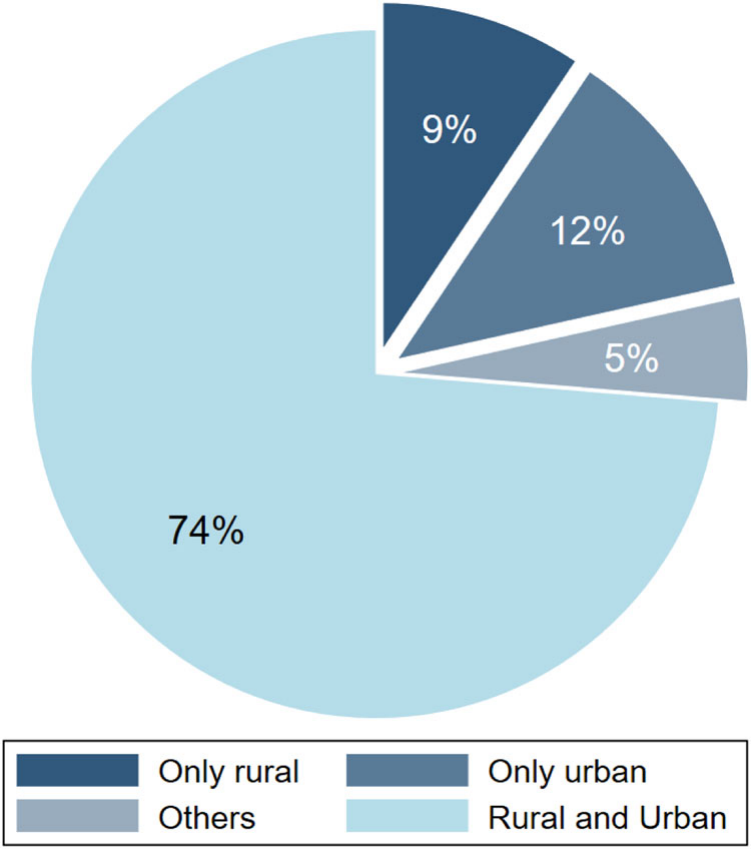
Distribution of population subgroups. *Source*: Authors' own calculation

Figure 9 shows the proportion of studies focused on rural areas or urban areas, or both. Most studies looked at both rural and urban areas (74%, *n* = 325). About 9% of the studies (*n* = 41) focused on rural areas; 12% (*n* = 51) focused on urban areas. Among the remaining studies, 14 studies looked at conflict affected population, 4 were studies of ethnic minorities and 2 studied refugees.

#### 5.2.6 Studies by fragile context

**Figure 10:**
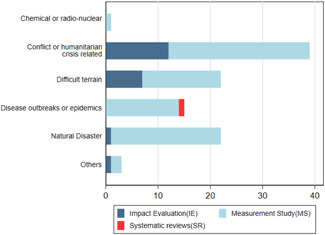
Number of studies in fragile contexts. *Source*: Authors' own calculation

We used the OECD definition of fragile context that includes conflict, institutional, social fragility, environmental, health and climatic risks (OECD, 2018). Figure 10 shows that 91 studies included on the map (21%) are from countries considered to be fragile. About 39 studies were conducted in a conflict or humanitarian crisis context; 22 studies each were conducted in contexts of difficult terrain and natural disasters; and 15 studies were conducted in the context of epidemics or disease outbreaks. There was one measurement study in the context of a chemical/radio‐nuclear disaster. IEs follow the same pattern, except for one notable gap: there are no IEs in the context of epidemics or disease outbreaks despite a reasonably good number of measurement studies.

Table H4 in Appendix G shows that satellite data are the most used in fragile contexts, followed by CRD data and then the sensor data. The table also shows that almost all the big data sources have been used in one or two fragile contexts, indicating the importance of big data in fragile contexts.

### 5.3 Risk of bias in included reviews

**Figure 11:**
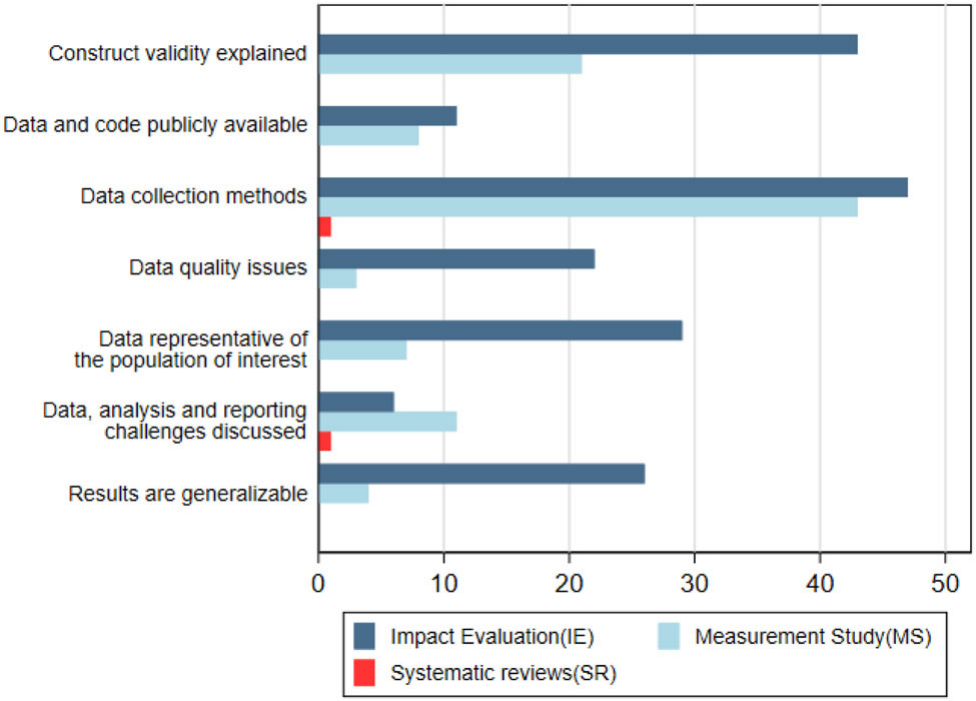
Number of IEs and MS against data quality and transparency. IE, impact evaluation. *Source*: Authors' own calculation

Figure 11 shows that very few studies meet any of the following methodological quality markers.

- Is the construct validity explained (ie is there a discussion on how the big data‐based indicator measures what the study claims to measure)?
- Are data and codes publicly available for replication?
- Are data collection methods discussed?
- Are there data quality issues in the dataset used and how are they addressed?
- Is the data representative of the population of interest?
- Are challenges in the analysis and reporting process discussed?
- Are the results generalisable? For example, are the research findings generalisable to other situations such as other platforms (data sources) or communities, or over time?

Only 95 studies (22%) have reported on at least one of the above transparency criteria. For example, only 20% (*n* = 91) of the total studies reported on data collection methods, 6% (*n* = 25) on data quality issues, 8% (*n* = 36) on data representativeness, 14% (*n* = 64) on construct validity and 7% (*n* = 30) on generalisability. Only 4% (*n* = 19) of the studies have data and codes publicly available or available upon request.

There is, however, considerable difference between IEs and measurement studies in terms of reporting on data quality issues and transparency. Table H4 in Appendix G shows that IEs report a lot better on all these parameters. Of the total 48 IEs on the map, 46 of them report at least one aspect of transparency. Almost all the IEs report on data collection methods, 90% (*n* = 43) report on construct validity, 60% (*n* = 29) discuss representativeness of data, 54% (*n* = 26) discuss generalisability and 45% (*n* = 22) discuss various data quality issues. However, only 23% (*n* = 11) make data and codes available and 13% (*n* = 6) discuss key data analysis and reporting challenges. Figure 11 shows that very few studies meet any of the following methodological quality markers.

- Is the construct validity explained (ie is there a discussion on how the big data‐based indicator measures what the study claims to measure)?
- Are data and codes publicly available for replication?
- Are data collection methods discussed?
- Are there data quality issues in the dataset used and how are they addressed?
- Is the data representative of the population of interest?
- Are challenges in the analysis and reporting process discussed?
- Are the results generalisable? For example, are the research findings generalisable to other situations such as other platforms (data sources) or communities, or over time?

Only 95 studies (22%) have reported on at least one of the above transparency criteria. For example, only 20% (*n* = 91) of the total studies reported on data collection methods, 6% (*n* = 25) on data quality issues, 8% (*n* = 36) on data representativeness, 14% (*n* = 64) on construct validity and 7% (*n* = 30) on generalisability. Only 4% (*n* = 19) of the studies have data and codes publicly available or available upon request.

There is, however, considerable difference between IEs and measurement studies in terms of reporting on data quality issues and transparency. Table H4 in Appendix G shows that IEs report a lot better on all these parameters. Of the total 48 IEs on the map, 46 of them report at least one aspect of transparency. Almost all the IEs report on data collection methods, 90% (*n* = 43) report on construct validity, 60% (*n* = 29) discuss representativeness of data, 54% (*n* = 26) discuss generalisability and 45% (*n* = 22) discuss various data quality issues. However, only 23% (*n* = 11) make data and codes available and 13% (*n* = 6) discuss key data analysis and reporting challenges.

## 6 DISCUSSION

### 6.1 Summary of main results

The use of big data in measuring development outcomes has been on the rise over the past 5 years. This rising trend is powered by the availability of (and our capacity to process) big data. In this section, we discuss the key findings, some of the notable gaps and the potential for future SRs.

#### 6.1.1 There is a considerable potential for measuring various development indicators using big data

We identify a significant and growing evidence base of measurement studies that use some form of big data to measure a development outcome. Some outcomes are more amenable to the use of big data than others; environmental sustainability, economic development and livelihoods, health and well‐being and urban development are where the majority of studies are concentrated. Education, sanitation, governance and human rights seem to be less responsive to big data use.

Multiple entries for most development theme indicate the potential of big data in contributing to measuring development indicators. Identifying measurement studies will be a valuable addition to development evaluators who look for innovative ways to measure a development outcome that was difficult to measure at all required spatial and temporal scales using conventional data collection methods.

#### 6.1.2 There is potential for more IEs using big data on development interventions

The map contains 48 IEs. Use of big data measures in IEs as main outcomes or for controlling key covariates is fast‐growing, but the IEs are fewer in number compared to measurement studies as well as in terms of the extent of their thematic and geographical coverage. IEs seem to be concentrated more around environmental sustainability, economic development and urban development. This complements existing efforts to build the evidence base in international development, as these sectors have much less rigorous evaluations (Sabet & Brown, 2018). The IEs also concentrate on using satellite data.

#### 6.1.3 Satellite data are used most

The map shows that 71% of the measurement studies and 81% of the IEs used satellite data. This is also one of the sources that has been used since the early 2000s. The prominence of satellite data are primarily due to the fact that satellite images offer unique possibilities for measuring and evaluating development outcomes. Given the vast number of satellites covering almost every location on earth, it is possible to collect data at a high granularity (spatial resolution) and for multiple temporal frequencies for the past 30 years. Satellite data are freely available from several sources (such as NASA's Landsat and MODIS and the European Space Agency's Sentinel); more importantly, several preprocessed databases are available (such as AidData's Geoquery,††† Yale University's G‐Econ Project, the United Nations Environment Programme's Environmental Data Explorer,‡‡‡ NASA's Socioeconomic Data and Applications Center§§§ [SEDAC], Global Forest Change 2000–2018 [Hansen et al., 2013], and several others). This preprocessed data or the image data could then be processed and converted into meaningful outcomes to measure economic activity at local level, urban development, forest cover, land productivity, distribution of the population, and so forth. These indicators can also be used for controlling for covariates.

#### 6.1.4 Spatial dimension matters

One of the key findings of the map is that most of the big data studies are applied in the context where the phenomenon studied has a spatial dimension, meaning the outcome and other covariates are measured on a spatial scale. Close to 70% of the studies on the map report using administrative units as their unit of measurement. This is particularly true for satellite and sensor data‐based studies, as 82% have administrative units as their unit of measurement (such as local economic development, agricultural land productivity, forest cover or urban development). This is referred to as geospatial IE (BenYishay et al., 2017). However, there is considerable difference across data sources as CRD data are used to measure changes at the population level.

#### 6.1.5 CRD data has great potential for measuring and evaluating development outcomes but is not yet used in IEs

CRD data are one of the most widely used sources in measurement studies. This is used for measuring population movement, migration, disease spread, and so forth. Despite a number of high‐profile measurement studies, our systematic search did not find even one IE that used CRD data for rigorously evaluating a development outcome. This is a notable gap and a potential area for future exploration. It should be noted that CRD data are also fraught with multiple methodological challenges (such as nonrepresentativeness, lack of completeness, etc.) and ethical challenges (such as consent, unintended exclusion, etc.). Further, CRD data has been difficult to obtain as it is proprietary and hence it is difficult to maintain data transparency.

#### 6.1.6 Other big data sources such as human‐sourced and process‐mediated data have good proof‐of‐concepts

Human‐sourced data (such as social networks, internet searches, mobile data content citizen reporting or crowdsourced data) and process‐mediated data (such as data produced by public agencies and by businesses) have a good number of measurement studies as proof‐of‐concept for using these sources to measure various development outcomes, but not many IEs use these sources. This also shows the possibility of potentially using these sources in future IEs. Similar caveats on methodological and ethical challenges discussed above in relation to CRD data will apply.

#### 6.1.7 East Africa is well‐represented, but not the rest of Africa

The geographical distribution of measurement studies and IEs show that the studies are evenly spread across the continents. However, Ethiopia, Kenya, Rwanda, Tanzania and Uganda are well‐represented in terms of number of measurement studies and IEs, but the only non‐East African country that seem to have well‐represented on the map is Nigeria. There are very few studies in the rest of Africa. This gap is particularly serious given Africa's data challenges (Serajuddin et al., 2015).

#### 6.1.8 Big data holds great potential for conducting IEs in fragile contexts, including during conflicts, humanitarian crises, epidemics and natural disasters

Conducting rigorous evaluation in fragile contexts (such as natural disasters, disease outbreaks and other crisis contexts) can be costly, risky to the beneficiaries and the evaluators, and in some cases outright infeasible. We identified 73 measurement studies, 17 IEs and one SR in such fragile contexts. Measurement studies are spread evenly across conflict or humanitarian crisis, disease outbreaks or epidemics, natural disasters and difficult‐to‐reach terrain. However, the IEs are concentrated around conflict and difficult terrain. The number of measurement studies indicate the potential for more IEs in fragile contexts.

#### 6.1.9 There are potential sectors and themes where SRs will be useful

Though the number of IEs are fewer, the map highlights a few potentials thematic areas where SRs will help answer key questions on policy and research methods. For example, there is a concentration of IEs using satellite data, referred to as geospatial IEs, but we know little about how satellite data can help evaluate development programmes better, where it can add value, what type of interventions could be better evaluated and the technical challenges involved in using satellite data. An SR of all geospatial IEs that have used satellite data across the sectors will help understand the potential and challenges in using satellite data for IEs.

Similarly, there is a concentration of IEs on environmental sustainability and within that climate action and forest management. Though there a few SRs and evidence gap maps on forest management (Pelletier et al., 2016; Puri et al., 2016), a new review with reference to innovative, new data sources used in rigorously evaluating forest cover and the advantages and challenges thereof may be useful.

### 6.2 Using big data in IEs: Potentials and challenges

Rigorous IEs require a valid counterfactual. Randomising programme placement ensures preprogramme comparability of the treatment and control groups in most cases and quasi‐experimental studies employ statistical procedures to identify a valid comparison group. In either case, evaluators collect require a vast array of data on the outcomes, covariates and other contextual factors. There is almost always a trade‐off between collecting a complete array of necessary data and cost‐effectiveness, and in a few cases, it may not be feasible to collect some of the covariates and confounders.

Big data, with the help of improved ML techniques and analytical capacity, can now be manipulated to evaluate development outcomes. The potential advantages for big data are, to date, most discernible in contexts where the immediacy, scale and/or reach of data are highly prized and alternative sources of data are absent or inadequate to the task. The ability to “zoom in” on particular zones of interest, and to produce estimates for small areas, is an oft‐cited advantage of many types of big data (e.g., satellite and building footprint data, mobile phone CRD and signalling data and app‐based location data) and one with particular relevance to evaluative contexts and SDG‐related urbanisation, climate change and infrastructure. This holds particular promise for settings where census data renders small area estimation methods unsuitable. Big data has also been shown to be particularly advantageous for the analysis of disaster‐induced displacement and disease outbreaks. In each of these cases, the advantage of big data is that it can support rapid appraisal and introduction or adjustment of policies/interventions on the basis of near real‐time information.

In this section, we highlight a few examples from the map to show the steps involved in collecting, processing and using satellite and CRD data for measuring development outcomes. We draw on recent projects from 3ie and Flowminder to illustrate the processes.

#### 6.2.1 Using satellite data in IEs

In a 3ie funded evaluation conducted by the Institute for Financial Management and Research, Pande and Sudarshan (2019) evaluated the recent environmental clearance (EC) reforms in India. Before the 2006 reform, mines of area over 25 hectares were required to hold a public hearing before approval. The new EC reform required mines of area between 5 and 25 hectares to hold a public hearing as well. This study exploits this historical discontinuity in clearance requirements to evaluate the impact of public hearings on mines' environmental compliance. Apart from rigorously evaluating the EC process in India, this study also provides a proof‐of‐concept for the use of remote sensing data and other publicly available data to monitor mines' environmental compliance. Using satellite data to assess the impact of EC process requires data on the timing of the intervention, the geographical scope of the intervention (ie the individual mines in this case), the outcome of interest (such as air pollution, land cover and water quality for the corresponding intervention) and control areas for the years before and after the intervention.

The following were the key steps involved in the big data IE.

**Step 1**: the researchers used web scraping techniques to collect information on the mines from their EC application for the years from 2006 to 2016, available online in a database published by Ministry of Environment, Forests and Climate Change. These are all mostly scanned PDF documents. The researchers scrapped for the information on project name and location (the tehsil and village where the mine is located); the dates of key EC stages of submission, review and approval; and mine characteristics such as minerals mined, mine production capacity and size of the mine. They also scrapped the clearing letters available in the same database for cross checking the data. They collected information about all 934 relevant mines and used 134 of them in their regression discontinuity analysis. Finally, the researchers hired ML Infomap, a local company, to geocode all the mines identified.

**Step 2**: Satellite data on various key environmental outcomes such as air pollution, land cover and water quality were collected from different sources.***

- The researchers used the data provided by Dalhousie University on the fine particulate matter concentration as a proxy for air pollution. This database contains average annual particulate matter concentration for every 1 km cell for the study period;
- They have used the Enhanced Vegetation Index (EVI) data from NASA's MODIS satellite to measure deforestation around the mining areas. EVI is available at a resolution of 250 metres for the entire globe and the researchers calculated annual maximum, median and mean EVI at mine sites. EVI data was used to measure the extend of and the date of beginning of deforestation (ie structural break in the time series) for each mine; and
- Data on water quality from the site monitor nearest to each mines was collected from the Central Pollution Control Board's ENVIS database. They used Biological Oxygen Demand, a measure of organics pollution, as a proxy for water pollution.

**Step 3**: The researchers then linked the geocoded mine sites to the corresponding cells of environmental outcomes. Of the total 934 mines in their database, they could link 889 of 1 km cells of EVI data and 882 of 250 metre cells with corresponding geocoded mine sites. They could also link 538 site monitors to the mines.

**Step 4**: The new EC reform required the mines of between 5 and 25 hectares in area to hold a public hearing that had not been considered big enough to hold public hearings during the previous regime (ie only the mines of above 25 hectares in area were required to hold the hearings). This study exploited the discontinuity around the 25 hectare mark and compared the mines marginally above 25 hectares with the ones marginally below 25 hectares. The final sample included 134 mines, of which 68 were treatment mines (<25 hectares) and 66 were control (>25 hectares). Using data before and after the EC applications, they estimated a difference‐in‐difference model.

This study, utilising web scraping to collect data on project characteristics and various sources of satellite data for measuring the outcomes of interest, is an excellent example of innovative data collection methods in a sector where the evidence base is very small (Rathinam et al., 2019b).

#### 6.2.2 Using CRD analytics to inform disaster management

In this section, we briefly outline the process for undertaking CRD analytics to measure, characterise and predict population displacement and returnee/resettlement patterns in post‐disaster settings. While applications of CRD data analytics to date have lacked an evaluative component, their potential in this regard is evident. We draw on a recent project at Flowminder, which revisited three sudden‐onset disaster events to investigate drivers of displacements (individual and contextual) and the feasibility of predicting displacement locations from CRD data and data on disaster intensity and damage, on population density and on the humanitarian response. The three events were the 2010 earthquake in Haiti, the 2015 Gorkha earthquake in Nepal and the 2016 Hurricane Matthew in Haiti.

Flowminder has long‐established partnerships with the major mobile phone network operators in Haiti and Nepal. Historically, data access has been a major barrier to the scale‐up of CRD analytics for humanitarian and development applications. Mobile network operators (MNOs) are justifiably hesitant to authorise third‐party access, given the need to safeguard subscribers' personal data.††††

Prior to analysis, MNO data underwent a long series of cleaning and preprocessing steps as part of quality assurance and to support the generation of standardised metrics. A first stage of analysis was undertaken to structure the data in a usable format and to detect data anomalies. Once data was cleaned, quality assured and converted into an analysable format, a number of preliminary processing steps were undertaken, including:

- Clustering of cell tower locations
- Assessment of each subscriber's phone usage behaviours (number of events, frequency and regularity)
- Determination of a predisaster “home” location.

#### 6.2.3 MNO data: Preprocessing steps

Here are some commonly occurring issues in MNO data. Once identified, corrections and/or accommodations can be performed prior to and/or during the preliminary processing and analysis phases.

1. **Standard data quality issues applicable to MNO data:**

- Item missing data (incomplete data records ie missing fields)
- Invalid entries for fields
- Duplicate records
- Interrupted data series' (e.g., no data for a particular time period)
- Inconsistent values (either in format, or definition) for keys that are used to join multiple datasets together
- Inconsistent entries for the “same” value (e.g., different spellings of the same place name).

2. **Issues specific to MNO datasets, CRD:**

- Inconsistencies or errors in method used for “hashing” (a form of pseudonymisation) subscribers' IDs
- Inconsistent “hashing” of sender and recipient IDs for communication events (e.g., standard SMS or phone calls).

3. **Issues specific to MNO datasets: Cell location and coverage maps:**

- Cell locations occur outside national borders
- Updated cell locations are not consistent with previous cell locations
- Small deviations in updated cell locations, possibly due to inexact global positioning system measurements
- Data, projection and coordinate system information are often missing in coverage datasets
- Inconsistent output formats
- For best server or cell‐in‐isolation maps, polygons should be labelled in a manner consistent with cell table and/or CRD dataset.

4. **Common data anomalies, indicative of a network issue or a sudden change in subscribers' behaviour due to an event or “shock”:**

- Individual cell towers have significantly more/less traffic than normal
- Overall network traffic is significantly higher/lower than expected
- Traffic from a particular region is significantly higher/lower than expected.

The processing steps undertaken to discern at individual level disaster‐induced displacements from pseudonymised, time series CRD data are presented below in Figure 13.

**Figure 12:**
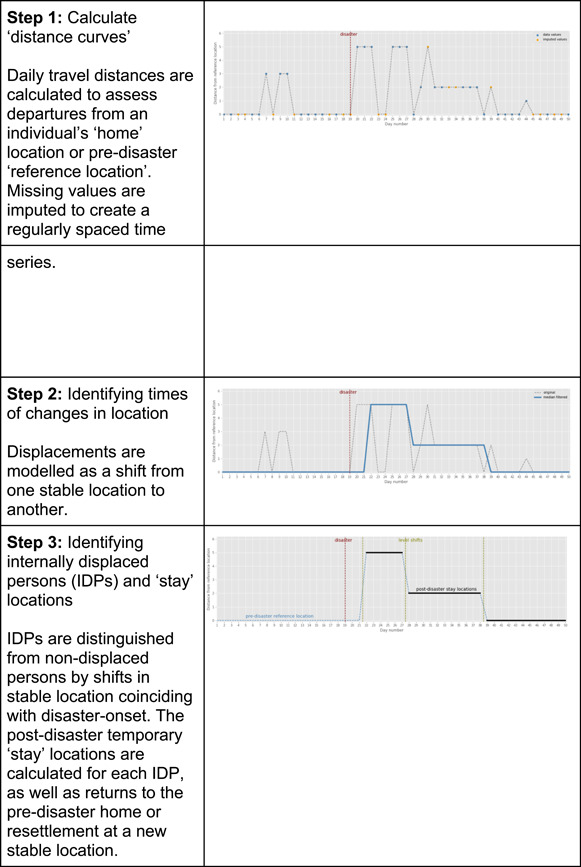
Number of studies reporting on ethics issues

**Figure 13:**
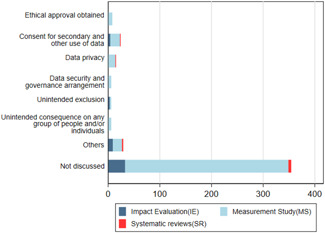
Steps in CRD processing for displacement and return/resettlement/recovery pattern analysis. CRD, call record detail

The analysis disclosed striking commonalities in internally displaced person (IDP) return/resettlement rates, with the fraction of IDPs who remain displaced exhibiting a common rate of decay across all three post‐disaster settings studied.

In a further step, the team developed new mobility and social network metrics to permit analysis of the relationships between contextual and individual variables and displacement duration, distance and trajectories, controlling for the severity of impacts and humanitarian response. The results suggest that the dispersal of an individual's social contacts and travel history predisaster are highly predictive of their post‐disaster displacement trajectories. Individuals with localised travel patterns and social contacts were more likely to be displaced in the vicinity of their usual residence compared with those with more dispersed travel patterns and social contacts. A majority of IDPs remained within a 10 km radius of their usual place of residence. Across the three disasters, 60–70% of long‐distance displacements (in excess of 100 km) involved travel to a familiar location and/or proximate to one or more contacts discernible in the predisaster CRD data. This pattern holds controlling for the severity of impacts at local area level and is consistent across all three disasters.

Results were validated via comparisons with reports retrospectively quantifying population displacements produced by the International Organisation for Migration, as well as with reference to data on the intensity of each disaster's impact on affected areas. The results indicate that CRD data analysis can be used to predict the estimated number and spatial distribution of IDPs at different time points based on initial estimates of the number of persons displaced in the immediate wake of a disaster, as well as to predict recovery/resettlement timelines. This has important implications for post‐disaster humanitarian response and resettlement efforts. The same methods can support disaster resilience assessments and planning and provide a means to compare recovery and resettlement rates across different disaster events.

### 6.3 Areas of major gaps in the evidence

#### 6.3.1 Satellite data also presents misclassification problems

Researchers, however, point to several technical challenges in using satellite images that may provide misleading conclusions. For example, Jain (2020) argues for the need for ground validation of satellite data as sometime the images could be misclassified (e.g., flood irrigation may be classified as flooded area); often this misclassification is systematic (ie forest cover is almost always misclassified as agriculture, which will bias the study results). This can be rectified with a field visit. Further, there could be differences in the data coming from different satellites and from the same satellite constellation but using different sensors (e.g., Normalised Difference Vegetation Index varies for different satellite sources and for same satellites across different versions, such as Landsat 5 and Landsat 8). Another example of what qualitative field visits could contribute to improving the interpretability of satellite data are the indicator “quality of roof construction” as a proxy for economic development. Straw roofs from satellite images are generally classified as low‐quality; zinc and other hard roofs are classified as a sign of development. However, several factors may bias this classification: in hot climates, straw roofs may be preferred for their ability to keep cool compared to other hard roofs; poor families may have received donations of high‐quality roofs; or lack of tenured security may discourage people invest in immoveable roof‐tops despite increase in income.

#### 6.3.2 Data quality and transparency is paramount

The map also points to the need to set standards for better reporting, as about 87% of the measurement studies did not report on data quality issues, representativeness, construct validity and generalisability. This would lead to questioning the internal and external validity of the findings. There is also a need to set standards for data transparency, taking into consideration the challenges in sharing proprietary data, data storing and the capacity of the Dataverse (see Box 1 for more details on data transparency).

Box 1 **Transparency in data analysis, use and sharing**

In recent times, the use of big data for in‐depth comprehension of developments in the social sector has gained traction. Transparency refers to publishing all relevant materials, including data and code, used in a research study in the public domain for independent verification. Transparency in research encompasses a number of elements that are no different while using big data as opposed to traditional data sources. Certain challenges that might arise are discussed below.

1. *Deidentification*: Deidentification is related to preserving the identity of the study subject before it is made available for any sort of analysis. A few concepts used in de‐identification of big data are K‐anonymity, L‐diversity and T‐closeness. A dataset is said to have K‐anonymity if each person in the dataset cannot be identified by information of the other K‐1 individuals in the dataset. In contrast, L‐diversity is a group‐based anonymisation technique that reduces the granularity in the dataset. T‐closeness is a refinement of L‐diversity and is used to decrease granularity over and above L‐diversity. However, there are several examples of combining different source to re‐identify the respondents in the data set (Archie et al., 2018).
2. Scale and storage: In today's world, storage of a high volume of data are not a challenge owing to developments in cloud computing. A fairly new system that provides solution to the scalability and storage issue is storage virtualisation. In simple terms, this is a network of storage devices that are combined to create a single storage space. A few ways of safeguarding the data storage is encrypting all processes and the usage of hybrid clouds. Data repositories such as Harvard dataverse and figshare have limited capacity to handle big data and are often restricted by the size of the data uploaded. Cloud storages such AWS and other similar such storage will be a better option.

#### 6.3.3 Ethical concerns are substantial

Ethical challenges such as consent, data privacy, data security and unintended exclusion are well documented in the literature (Lokanathan et al., 2017; York & Bamberger, 2020). A brief analysis of the studies on the map shows that very few studies report on any of these ethical challenges. However, the challenges are different for different sources of big data. For example, satellite data that involves little human interaction may not need an IRB review but most other big data source that use human‐generated data without explicit consent for secondary use should be reviewed by IRBs. We also recommend more mixed‐method big data evaluations to mitigate the potential disconnect between development stakeholders and big data researchers. Any mixed‐method research needs to be reviewed by IRBs.

The map shows that most IEs have done well on reporting data quality issues but not on ethical issues. Since big data involves ethical issues (such as consent for secondary data use and unintended exclusion) that are new to conventional ethical standards, there is a need to update the current ethical standards practice to include big data use as well.

#### 6.3.4 Big data may be growing in use and popularity, but the need for independent auxiliary data for “ground‐truthing” remain

Many sources of big data are partial in terms of coverage and prone to biases that are difficult to measure, control and correct for in the absence of secondary data. Despite growing awareness and acknowledgement of its limitations, the household sample survey remains the dominant source of development policymaking. Big data often require survey data as “ground‐truth” data to validate the findings. Demographic Health Surveys and Living Standards Measurement Studies are the two main surveys used in ground‐truthing. There is considerable scope for merging the income and expenditure surveys, and food surveys conducted in several developing countries with big data to assess food shortages, poverty hotspots, and so forth.

#### 6.3.5 Some capacity constraints are acute

Development organisations need to build staff capacity in order to use big data as a strategic asset (Perera Gomez & Lokanathan, 2017). They need to build multidisciplinary teams consisting of data experts and subject matter professionals, and also compete with the private sector to recruit the staff. Other major costs involve scaling up the technical infrastructure to enable data storage and processing on a large scale and data accessibility costs. The latter can be more difficult to predict considering that big data sources that are currently public may involve licensing in the future. Besides, ensuring the sustainability of data can be a cause of concern. As suggested by Hammer et al. (2017), as most of the big data is produced as a by‐product by the private sector, continuity of data provision cannot be guaranteed in this age of evolving technology and market conditions. These concerns will call the wisdom of committing resources upfront to build capacity into question.

#### 6.3.6 Need for better coordination between data scientists and evaluators

Big data analysts and evaluators use different framework and analytical tools. In particular, the big data measurement studies look for hidden patterns in the data with little support from theory and aim at prediction rather than causality (York & Bamberger, 2020). Further, the expertise needed to analyse big data remains largely localised and siloed. Outside of a small and highly specialised group of data scientists, there is uncertainty about how best to carry out large‐scale big data analysis. The degree of technical specialisation combined with strict access restrictions to many types of big data has hindered big data applications in development evaluation. Hence, there is a need to promote interaction between development evaluators and data scientists for better cross‐learning and adoption of big data in measuring and evaluating development outcomes.

#### 6.3.7 The cost of collecting, analysing, storing and reporting big data is largely unknown

There is very little publicly available information on the cost of collecting, analysing and reporting big data. Blumenstock et al. (2015) reported that the phone survey for ground‐truthing the CRD data costed USD 12,000 and took 4 weeks to administer. This is, however, only the variable cost of data collection in this study. There are multiple hidden costs such as staff costs and the cost of the necessary computing infrastructure (including storage); in addition, the opportunity cost of time involved in developing partnerships with data providers in some cases is not known. BenYishay et al. (2017) report that the cost of geospatial IE is around USD 150,000. One of the 3ie funded studies using satellite data are reported to have spent USD 3300 on data collection, which is about 1% of the total study budget, but have spent USD 103,864 on data analysis and reporting (about 32% of the total cost‡‡‡‡). Similarly, another 3ie funded study that used in situ fixed sensors reported spending USD 6152 on data collection or acquisition (11%) and USD 54,444 on staff costs for analysis and reporting (55%). However, studies that have combined satellite data with household survey have reported higher costs of data collection (USD 171,582; 43%) and analysis and reporting (USD 109,495; 27%). This is, however, roughly comparable to the data collection cost of an average 3ie funded multi‐year, multi‐round survey IE, which costs about USD 176,000 (Puri & Rathinam, 2019).

#### 6.3.8 Evaluation of potential methodological issues

While big data can help resolve many data related challenges, there are considerable methodological, analytical, logistical and ethical challenges in the way of using it in measuring development outcomes (Letouzé, 2016; Lokanathan et al., 2017; Olteanu et al., 2019; Salganik, 2017). This section briefly discusses some of the prominent methodological challenges. As big data is varied in type, quality and composition, we also discuss if the challenges are specific to any particular type of big data.

We have grouped all the big data challenges that may affect measuring development outcomes credibly and lead to questionable internal and external validity of the studies.

##### Nonrepresentativeness of data and selection bias

Big data may unintentionally exclude certain sections of the population or marginalised communities, thereby making the sample unrepresentative of the population being analysed. Large samples do not solve this systematic bias. This, however, is not a challenge when using satellite data that has universal coverage but, with human‐generated and CRD data, nonrepresentativeness is a serious challenge. Human‐generated data such as Twitter, Facebook or web searches, as well as mobile phone use that generate CRD data, are not representative as the usage is limited by income, education, infrastructure, and so forth. However, clarity on what is the sample frame (ie who is included and who is excluded) will help interpret big data results appropriately. Nonrepresentative sample is still useful for within‐sample comparisons, but may lead to erroneous out‐of‐sample generalisations (Olteanu et al., 2019; Salganik, 2017).

##### Construct validity

Construct validity is whether the proposed measure actually measures what it claims to be measuring. This becomes important when the construct is unobservable and has to be operationalised via some observed attributes (Olteanu et al., 2019). For example, does night light data truly reflect local GDP and other development outcomes such as health and education? What is it in the CRD data that reflects people's income or employment status? In many cases, the big data‐based measures may not be straightforward, and it is good practice to clearly state construct validity and provide necessary support to back the claim in the papers. Development measures based on social media are particularly challenging due to different communication styles, special usage of terms and differences in language proficiency.

###### Data quality issues

- *Comparability of data over time*: Since most of these data are collected routinely as a part of business, the nature and quality of data may change with the technology and business requirements. This may happen because the underlying technology has changed or because the people who use it have changed. For example, satellite data are not readily comparable across the years as there is a vast quality difference (Jain, 2020); good flu trends based on online searches peaked comparing to officially reported data when the underlying Google algorithm started prompting people to query more and broke the relationship between Google searches and flu prevalence (Archie et al., 2018).
- *Lack of completeness*: Most big data is a by‐product of peoples' everyday action and/or result of system logs of the government and businesses. It may not contain all the necessary information, such as demographic characteristics. However, combining multiple sources of data, especially big data and administrative data, can help resolve this problem (Salganik, 2017).

##### Generalisability

Generalisability or external validity refers to the applicability of the findings of a study to population or context other than it was produced. In the context of big data, generalisability would mean the applicability of the model to a setting different from the setting of the data that the model was trained on. For example, a model trained on satellite data from a specific geographical region may not be generalised (Head et al., 2017; Jean et al., 2016). It is good practice for studies to report on the representativeness of training data.

##### Data transparency

Transparency in this context refers to publishing all relevant materials, including the data and code, used in a study in the public domain for independent verification. Sharing of raw data in the public domain is often crucial for establishing confidence and reliability in the results. There are two challenges here: first, some of the major sources of big data (such as CRD) are proprietary and sharing may not be permitted beyond the closed group of researchers; and second, the data has to be de‐identified before it can be shared and it is crucial to check whether there are variables or a combination of variables that can be used to reidentify research subjects.

We assessed whether the studies included in the systematic map asked the following questions:

- Is the data representative of the population of interest?
- Is the construct validity explained (ie is there a discussion on how the big data‐based indicator measures what the study claims to measure)?
- Are there data quality issues in the dataset used and how are they addressed?
- Are the results generalisable? For example, are the research findings generalisable to other situations, such as other platforms (data source) or communities, or over time?
- Are data and codes publicly available for replication?

#### 6.3.9 Evaluating reporting on privacy and ethical considerations

There are concerns over data access, privacy, consent and ethics in using big data. Although these are foundational issues for both small and big data studies, the challenges posed by big data have greater repercussions.

When using big data sources such as mobile data, most mobile operators have “inform and consent” policies that mandate disclosure of all relevant information to potential participants who can then evaluate this information and give explicit permission. However, these policies often contain legal language that is generally not discernible, and it is not clear if explicit consent is obtained to repurpose the data. This kind of informed consent may be completely absent in research leveraging social media data due to the impracticality of obtaining consent from millions of users.

Mobile phone user data and social media data are some of the most used sources of big data that can inform researchers about individuals' behaviour. Even if the data are de‐identified, concerns still remain over the consent and ethics of sharing such data with researchers. It is thus imperative to have an ethics approval process in place that lays down the conditions under which such research can take place. There is a need for clear ethical standards for big data research and studies should be monitored by the IRBs.

Another ethical criterion when using big data can be concerned with the assessment of risks, the most common being privacy breaches leading to identity theft or other cybersecurity risks. The possibility of the reidentification of any individual user from poorly anonymised datasets adds to the concerns over anonymity of subjects. When combined with other sources, such datasets can be used to gain detailed insights about people without their knowledge. Such precise inferences may create the capacity for discrimination or mass manipulation. Sometimes data obtained for one purpose in social data research is used for secondary analyses, but the associated risks may not be well understood. For example, Facebook data in the past has been used for ad targeting, as well as for tailoring propaganda (Horowitz et al., 2018).

Big data may also inadvertently exclude certain sections of the population. For example, this bias can be observed in the case of “Street bump,” a mobile app that notifies the Boston City Hall whenever the user hits a bump on the road (Carrera et al., 2013). The data includes information only from the app users who often use both their cars and the app; this might inadvertently exclude poorer parts of the city that app users may not frequent. Policy based on such big data sources may have unintended consequences for the people who are excluded.

We assessed the studies on the following:

- Ethical approval obtained
- Consent for secondary and other use of data discussed explicitly
- Discussions on data privacy
- Discussions on data security and governance arrangement
- Discussion of any potential unintended exclusion
- Discussion of potential unintended consequences for any group of people or individuals.

#### 6.3.10 Reporting on privacy and ethical challenges

Figure 12 shows that most studies (81%) do not report on ethical challenges and privacy issues. Of the few that do discuss such challenges, the most frequently discussed issue is consent for data use.

### 6.4 Limitations of the systematic map

This map covers large thematic areas and outcomes corresponding to SDGs. Given the wide scope of the outcomes, the evidence is sparse and bunched around a few themes. The thematic gaps here may not be read as actual gaps, but these areas may be not readily relevant to using big data. This map rather shows what evidence or proof‐of‐concepts are available to measure and evaluate development outcomes using big data.

This map followed a systematic process of searching, screening and coding of studies based on a predefined set of criteria in the study protocol developed with inputs from key stakeholders. However, despite best efforts in searching and screening the studies, given the wide scope of big data sources and their application across all developmental themes and the pace at which the literature is growing, it is possible that some relevant studies (especially measurement studies) could have been missed out.

It was beyond the scope of the study to provide a critical quality appraisal of the IEs or the measurement studies, given the large number of studies included on the map; nor did the report look at the details of ML methods used in the included studies.

Given the wide scope of development applications, it was not possible to code the studies for all subclassifications. Though the submaps (especially for economic development and livelihoods, health and well‐being and urban development) provided coding at level 2 indicators, it was not possible to provide granular analysis of development themes corresponding to SDG indicators at level 3. Future systematic maps should aim to produce more granular classifications on the use of big data at the indicator level.

Some studies have used ML techniques for treatment effect heterogeneity in RCTs (Chernozhukov et al., 2019). However, it was beyond the scope of this report to include the role of big data analytical methods in conventional IE designs such as RCT and other quasi‐experimental designs. This is a nascent but growing body literature and could be considered for inclusion in future maps.

Several studies suggest that the key advantages of big data sources (especially satellite data) are their long‐term availability which will help evaluate the long‐term impact of development interventions. The possibility of collecting a vast array of information on several contextual factors using big data can help evaluate complex interventions (Bamberger, 2016). However, this map did not code the studies for long‐term impact or for complex interventions. Future maps may code and analyse the role of big data in measuring long‐term impact and in evaluating complex interventions.

### 6.5 Stakeholder engagement throughout the systemaic map process

The stakeholders in this systematic map included an advisory group comprised of sector experts, FCDO staff, CEDIL staff. All stakeholders were engaged in reviewing drafts and final reports associated with the map. The protocol for the map was developed with inputs from the advisory board, and FCDO and CEDIL staff. The advisory board played a key role in steering the search strategy and building the list of key words.

## 7 AUTHORS' CONCLUSIONS

Big data has great potential to help address questions of relevance to international development, including for evaluating the effects of interventions. This systematic map compiles IEs, SRs and measurement studies that incorporate big data to highlight how this innovative, new data source is being used to evaluate development outcomes and (more importantly) where there is more potential to use big data in the future evaluations. We found 437 studies, of which 48 are IEs, 381 are measurement studies and 8 are SRs. Roughly half the studies are from Asia and another 30% are from Africa; about 70% are from LMICs. Of the 48 IEs, 8 are RCTs and the remaining are quasi‐experimental studies.

Our results highlight considerable potential for using big data for measuring various development outcomes across SDG themes, but big data is more relevant to environmental sustainability, economic development and livelihoods, health and well‐being and urban development. This map also highlights that big data can contribute to the evidence base in development sectors where evaluations are not generally feasible due to a lack of data, particularly due to fragile contexts.

One of the key “absolute gaps” the map has identified is that the number of IEs is lower in comparison to measurement studies. Given the fast‐growing availability of big data and improving computation capacity, there is great potential for using big data in future IEs. This may not, however, be straightforward as there are several analytical, ethical and logistical challenges that may hinder the use of big data in evaluations. The development community that helps set standards and best practices and development stakeholders (including donors who facilitate rigorous evaluations and learning) have a strong role to play in facilitating this process. The report highlights the need for setting standards for better reporting on data quality issues, representativeness, construct validity and generalisability, as well as the need for data transparency and sharing. The report also calls for facilitating better interaction between big data analysts, remote sensing scientists and evaluators.

One of the key findings of the report is that satellite and sensor data are the most used data sources for both measurements studies and IEs. There are several sources of preprocessed satellite data that could be used directly in evaluations without the evaluators having to process them using complex ML models themselves. Satellite data seems to be particularly useful in the context where the development interventions and the outcomes studied have spatial dimension economic activity at the local level, urban development, forest cover, land productivity and distribution of the population, or where the outcome and other covariates are measured on a spatial scale (ie villages, counties, districts, plots or protected areas). CRD data, on the other hand, despite being used widely in measurement studies, is not yet used in IEs. The data deficiency in international development is partly due to fragile contexts such as diseases spread, violence, natural calamities and difficult terrain. This map highlights the potential of big data in fragile contexts: one‐quarter of the studies were conducted in such a context.

For evaluators and researchers, the report calls for better reporting on data quality, ethics and transparency. There is also an absolute gap in using mixed methods jointly with big data and cost‐effectiveness. For the donors, this report calls for more efforts on setting up best practices and ethical standards and in facilitating more interaction among remote sensing scientists, big data analysts and development evaluators.

### 7.1 Implications for research, practice and/or policy

- Reliable data are paramount to evaluating development outcomes and future resource allocation.
- This systematic map compiles the IEs, SRs and measurement studies to highlight how innovative, new data sources are being used in evaluating development outcomes, and more importantly where there is more potential to use big data in the future evaluations.
- This map shows that big data can contribute to evidence base in development sectors where evaluations are not generally feasible due data deficiency.
- Given the fast growing availability of big data and improving computation capacity, there is a great potential for using big data in the future IEs.
- There are several sources of preprocessed satellite data that could be used in evaluations directly without the evaluators having to process them using complex ML models themselves
- There is also an absolute gap in using mixed methods jointly with big data and cost effectiveness. This should be prioritised by donors and researchers as a mix of quantitative big data analysis and qualitative field level analysis will help strengthen the validity of the results.
- More efforts, on the donors' end, is required to set up best practices and ethical standards, and facilitating more interaction among remote sensing scientist, big data analysts and development evaluators.

## CONTRIBUTIONS OF AUTHORS

- Content: Francis Rathinam, Samantha Watson, Sebastian Vollenweider, Zeba Siddiqui, Manya Mallik, Pallavi Duggal, and Sayak Khatua.
- Map methods: Francis Rathinam.
- Statistical analysis: Sayak Khatua and Francis Rathinam.
- Information retrieval: Zeba Siddiqui, Manya Mallik, Pallavi Duggal, and Sayak Khatua.

## DECLARATIONS OF INTEREST

The authors declare no conflict of interest.

### PLANS FOR UPDATING THE SYSTEMATIC MAP

The systematic map shows that both IEs and measurement studies have dramatically increased in the past 5 years and are continuing to grow in number. Given the potential for faster growth in the availability and computational capacity, it is very likely that the number of studies will grow faster than we have witnessed over the past 5 years. Hence, we recommend that this map be updated within the next 2 years.

The fact that more than 80% of the included studies are peer‐reviewed shows the growing number of journals interested in big data application in international development. It will be useful to include a more exhaustive grey literature search to identify the full extent of the literature.

This map shows the potential for big data to measure and evaluate various development themes. However, most of these studies are supported by universities and specialist organisations and conducted by researchers associated with these organisations. Widely disseminating the findings of the map among development researchers, evaluators, practitioners and donors will help promote the adoption of big data measures in future IEs.

## Figures and Tables

**Figure A1:**
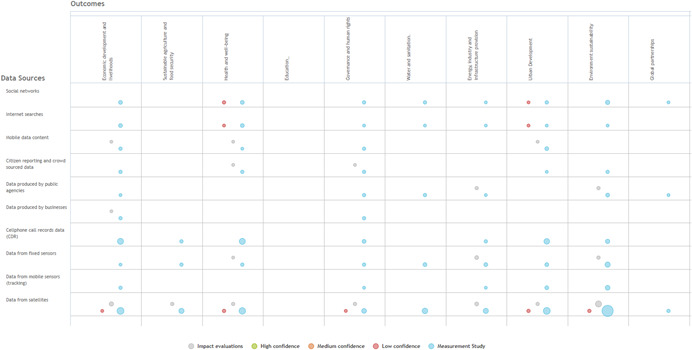
Systematic map of big data sources and outcomes. The online map can be accessed here: https://gapmaps.3ieimpact.org/evidence-maps/big-data-systematic-map

**Figure A6:**
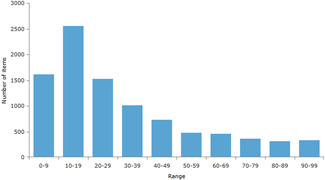
Order of relevance of studies at the title and abstract screening stage. *Source*: Authors' own calculation

## Footnotes

‡ The gap in data availability at the country level is partially driven by a lack of resources, limited capacity within governments and logistical difficulties in collecting the data. For example, the total cost of collecting data on all the 169 sustainable development goal (SDG) targets was estimated to be around USD 254 billion, which is about 12.5% of total official development assistance to be committed for the post‐2015 period (Jerven, 2014). A recent UN survey shows that there is existing capacity to collect data on only 40 SDG indicators and data sources for another 47 indicators are available in principle. There is little capacity and resources for collecting data on the remaining indicators (UN DESA, 2018).

§ For the purpose of this report, big data measurement studies are defined as the studies that have innovatively used big data to measure and validate any development outcome such as poverty measurement, crop productivity, employment, mobility, forest cover, and so forth. These are not IEs but can inform future evaluations.

** The online map can be accessed here: https://gapmaps.3ieimpact.org/evidence-maps/big-data-systematic-map.

†† 3ie (2012) impact evaluation glossary. Available at: https://www.3ieimpact.org/sites/default/files/2018-07/impact_evaluation_glossary_-_july_2012_3.pdf.

‡‡ There are also other classifications of big data based on its structure: structured, semistructured and unstructured data. Structured data are in the standard columns and rows form such as what sensors provide; semistructured data contains free texts but can be tagged, such as Twitter; and the unstructured data includes images and videos (Desouza & Jacob, 2017). Big data can also be classified as open and proprietary data (Maaroof, 2015).

§§ Our map refers to SDGs since they are globally acknowledged and fairly inclusive list of development outcomes. All the studies relevant to this map are expected to fall under at least one of the 169 targets listed within SDGs.

*** See Rathinam et al. (2019a) for more details on single screening with safety first approach. 9 See 3ie's critical appraisal checklist for SRs.

†††
http://geo.aiddata.org/query/.

‡‡‡
https://geodata.grid.unep.ch/.

§§§
https://sedac.ciesin.columbia.edu/.

***
*There are several ways to collect the required satellite data. For a few key variables such as night lights, air pollution, land cover and water quality, elevation, slope, distance from certain services or infrastructure, geocoded data for various granularity and frequency is readily available in several databases such as Aiddata DataQuary, SEDAC, and so forth. This study has utilised data from differences sources that provide readily useable data. Alternatively, the researchers use ML techniques to analyse satellite images and predict development outcomes* (Jean et al., 2016). *Another useful source of areal images come from custom built drones that can provide very high resolution data for the exact spatial and temporal frequency* (Pellegrini, 2019).

††††
*Flowminder's original “data partnership” model developed lasting collaborations with individual MNOs. The priority was countries where substatial potential gains were available from novel, digital data given the existing data landscape. Lengthy negotiations with MNOs followed, often spanning many years and consuming extensive organisational resources, with no guarantee of a successful outcome. When this model “worked,” it led to strong and sustained partnerships with MNOs. In pursuit of impact at scale, Flowminder Foundation supplemented its original “data partnership” model with a toolkit‐based approach designed to break down silos between data and methods, in effect negating the need for Flowminder to access MNO data by transferring methods expertise to MNOs themselves via the “Flowkit” suite of software*.

‡‡‡‡
*It should be noted that the cost discussed here includes only the variable cost of data collection and the staff time, but may not include the cost of fixed infrastructure and equipment*.

## APPENDIX B THE OECD DEFINITION OF FRAGILE STATES

OECD defines fragility as the combination of exposure to risk and insufficient coping capacity of the state, system and/or communities to manage, absorb or mitigate those risks. Fragility can lead to negative outcomes including violence, the breakdown of institutions, displacement, humanitarian crises or other emergencies. The OECD fragility framework considers not only current exposure to negative events such as natural disasters and armed conflict but also capacity to cope with likely future negative events.

The new framework considers five dimensions of fragility.

- **Economic fragility** is vulnerability to risks stemming from weaknesses in economic foundations and human capital including macroeconomic shocks, unequal growth and high youth unemployment;
- **Environmental fragility** is vulnerability to environmental, climatic and health risks that affect citizens' lives and livelihoods. Risk factors can be external or internal, including exposure to natural disasters; air, water and sanitation quality; prevalence of infectious disease; number of uprooted people; and vulnerability of household livelihoods;
- **Political fragility** is vulnerability to risks inherent in political processes, events or decisions; political inclusiveness (including of elites); and transparency, corruption and society's ability to accommodate change and avoid repression. Risk factors include regime persistence, state‐sponsored violence or political terror and levels of corruption;
- **Security fragility** is the vulnerability of overall security to violence and crime, including both political and social violence. Risks are measured by the homicide rate, level of violent organised crime, number of deaths from nonstate actors or terrorism, number of battle deaths from conventional warfare, and levels of domestic violence; and
- **Societal fragility** is vulnerability to risks affecting societal cohesion that stem from both vertical and horizontal inequalities, including inequality among culturally defined or constructed groups and social cleavages. Risk indicators include income inequalities (vertical) and social inequalities related to gender, growth in urbanisation and numbers of displaced people.

In the past few years, OECD has moved away from the “fragile states list” and towards measuring each of those five dimensions on a spectrum of intensity for 58 fragile contexts. This comes as a part of their effort to move towards a universal concept of fragility, recognising that it affects not only developing countries but all countries to some extent.

**Table B1:** Fragile contexts provided in the OECD fragility framework 2018 (decreasing order of severity)

Economic	Environmental	Political	Security	Societal
Central African Republic	Somalia	Eritrea	Syria	South Sudan
South Sudan	Central African Republic	Sudan	Libya	Yemen
Liberia	South Sudan	DPRK	Yemen	Syria
Somalia	Chad	Yemen	Somalia	Egypt
Solomon Islands	DRC	Syria	Afghanistan	Somalia
Afghanistan	Burundi	Somalia	South Sudan	Sudan
Niger	Mozambique	South Sudan	Iraq	Burundi
Sierra Leone	Guinea‐Bissau	Burundi	Sudan	Eritrea
Comoros	Niger	Chad	Central African Republic	DPRK
Guinea‐Bissau	Liberia	Congo	Nigeria	DRC
Mozambique	Burkina Faso	Gambia	Mali	Congo
Haiti	Guinea	Ethiopia	Pakistan	Chad
Mali	Sierra Leone	Guinea	Chad	Equatorial Guinea
Congo	Mali	DRC	DRC	Pakistan
Libya	Malawi	Mauritania	West Bank & Gaza	Iran
Syria	Zambia	Bangladesh	Cameroon	Central African Republic
Yemen	Cameroon	Angola	Egypt	Afghanistan
Tajikistan	Eswatini	Djibouti	Niger	Zimbabwe
Djibouti	Côte d'Ivoire	Venezuela	Haiti	Ethiopia
Gambia	Eritrea	Iran	Ethiopia	Kenya
Mauritania	Ethiopia	West Bank & Gaza	Kenya	Eswatini
Iraq	Afghanistan	Zimbabwe	Myanmar	Mauritania
Honduras	DPRK	Madagascar	Nepal	West Bank & Gaza
Timor‐Leste	Yemen	Afghanistan	Burkina Faso	Angola
Eswatini	Haiti	Central African Republic	Congo	Iraq
Pakistan	Syria	Libya	Eritrea	Guatemala
DRC	Tanzania	Egypt	Burundi	Gambia
Eritrea	Zimbabwe	Guinea‐Bissau	Mauritania	Cameroon
Burkina Faso	Madagascar	Iraq	Iran	Haiti
Burundi	Congo	Sierra Leone	Comoros	Uganda
Zimbabwe	Rwanda	Mali	Bangladesh	Rwanda
Ethiopia	Myanmar	Haiti	Venezuela	Lao PDR
Chad	Uganda	Côte d'Ivoire	Tanzania	Myanmar
Guinea	Pakistan	Mozambique	Uganda	Venezuela
Malawi	Comoros	Myanmar	Mozambique	Tajikistan
Madagascar	Bangladesh	Rwanda	Guatemala	Bangladesh
Rwanda	Tajikistan	Liberia	Côte d'Ivoire	Guinea
DPRK	Lao PDR	Nigeria	Sierra Leone	Libya
Zambia	Djibouti	Pakistan	Liberia	Djibouti
Uganda	Mauritania	Burkina Faso	Honduras	Guinea‐Bissau
Tanzania	Kenya	Uganda	Guinea	Tanzania
Papua New Guinea	Papua New Guinea	Kenya	Guinea‐Bissau	Honduras
Nepal	Angola	Honduras	Angola	Mozambique
Equatorial Guinea	Iraq	Niger	Gambia	Côte d'Ivoire
Lao PDR	Gambia	Tajikistan	Tajikistan	Madagascar
Myanmar	Sudan	Equatorial Guinea	Equatorial Guinea	Nigeria
Cameroon	Nepal	Cameroon	Lao PDR	Papua New Guinea
Angola	Timor‐Leste	Eswatini	Solomon Islands	Zambia
Bangladesh	Nigeria	Lao PDR	Madagascar	Mali
Guatemala	Guatemala	Guatemala	Papua New Guinea	Sierra Leone
Côte d'Ivoire	Solomon Islands	Zambia	Zimbabwe	Nepal
Kenya	Venezuela	Timor‐Leste	Zambia	Burkina Faso
Venezuela	Honduras	Nepal	Timor‐Leste	Malawi
Nigeria	West Bank & Gaza	Tanzania	Malawi	Niger
Sudan	Iran	Papua New Guinea	Eswatini	Timor‐Leste
Iran	Egypt	Comoros	Djibouti	Liberia
West Bank & Gaza	Libya	Malawi	Rwanda	Solomon Islands
Egypt	Equatorial Guinea	Solomon Islands	DPRK	Comoros

## APPENDIX C DETAILS OF SUBMAPS

**Table C1:** Outcome categories and definitions

Category	Definition	Subcategories
Economic development and livelihoods (SDG 1 and 8)	End poverty in all its forms everywhere	Poverty mapping/measurement
	Promote sustained, inclusive and sustainable economic growth, full and productive employment and decent work for all	Measuring wealth/GDP
		Access to basic services
		Social protection, including financial inclusion
		Vulnerability reduction
		Economic growth and productivity
		Full and productive employment, including youth unemployment
		Eradicate forced and underage labour, protect labour rights and promote safe workspaces
		Promote sustainable tourism
		Strengthen capacity of domestic financial institutions
Health and well‐being (SDG 3)	Ensuring healthy lives and promoting well‐being for all ages	Reduce mortality
		End epidemics of communicable diseases
		Strengthen prevention and treatment of substance abuse
		Decrease fatalities due to road accidents
		Universal access to sexual and reproductive healthcare services
		Achieve universal health coverage
		Reduce fatalities due to pollution
Governance and human rights (SDG 5, 10 and 16)	Achieving gender equality and empower all women and girls	
	Reduce inequality within and among countries	
	Promote peaceful and inclusive societies for sustainable development, provide access to justice for all and build effective, accountable and inclusive institutions at all levels	
Urban development (SDG 11)	Make cities and human settlements inclusive, safe, resilient and sustainable	Access to affordable housing
		Access to affordable transport systems
		Enhance inclusive and sustainable urbanisation
		Strengthen efforts to protect cultural and natural heritage
		Better disaster management
		Reduce environmental impact of cities
		Universal access to green public spaces
Environmental sustainability (SDG 12, 13, 14 and 15)	Ensure sustainable consumption and production patterns	
	Take urgent action to combat climate change and its impacts	
	Conserve and sustainable use the oceans, seas and marine resources for sustainable development	
	Protect, restore and promote sustainable use of terrestrial ecosystems, sustainably manage forests, combat desertification, halt and reverse land degradation and halt biodiversity loss	

## APPENDIX D SEARCH STRATEGY AND THE DATABASES SEARCHED

We developed a systematic search strategy in consultation with an information specialist after finalising the protocol.

We searched the following general databases:

- CAB Abst: www.cabi.org/publishing-products/online-information-resources/cab-abstracts/
- Econlit (Ovid): www.ovid.com/site/catalog/databases/52.jsp
- Ebsco Discovery: https://www.ebscohost.com/discovery
- Scopus: https://www.scopus.com/
- Social Sciences Citation Index (SSCI) (via Web of Science): https://library.maastrichtuniversity.nl/collections/databases/ssci/

Bilateral and multilateral agencies and general repositories of IEs in international development:

- 3ie Repository of IEs: www.3ieimpact.org/en/evidence/impactevaluations/
- 3ie RIDIE (Registry for International Development IEs): http://ridie.3ieimpact.org/
- USAID Evaluation Clearing House: https://dec.usaid.gov/dec/content/evaluations.aspx
- Innovations for Poverty Action: www.poverty-action.org/project-evaluations
- J‐Poverty Action Lab: www.povertyactionlab.org
- The World Bank: www.worldbank.org/
- AEA RCT Registry: https://www.socialscienceregistry.org/
- DFID Research for Development: http://r4d.dfid.gov.uk/
- Campbell Collaboration: www.campbellcollaboration.org
- African Development Bank: https://www.afdb.org/en/documents/publications/
- BREAD: http://ibread.org/bread/papers
- Center for Effective Global Action: http://cega.berkeley.edu/evidence/

We searched specialist organisational databases that might include big data measurement studies and IEs:

- AidData: A Research Lab at William & Mary: www.aiddata.org/
- Flowminder: https://web.flowminder.org/
- Stanford University Center on Food Security and the Environment: https://fse.fsi.stanford.edu/
- UN Global Pulse: www.unglobalpulse.org/
- Data‐Intensive Development Lab: http://didl.berkeley.edu/
- Global partnership for sustainable development: www.data4sdgs.org
- Arxiv database: (https://arxiv.org).
- Eldis: www.eldis.org

We also searched grey literature via Google Scholar and checked references of any SR that we find in our searches and met our inclusion criteria.

Given the relative recent state of the evidence base in this field and the fact that most programmes/initiatives started to flourish in the late 2000s we conducted the searches from 2005 onwards.

**Web of Science (SSCI and Science Citation Index), searched 22 September 2019**

# 1 34,746

TS= ("big data" or metadata or "meta data" or meta‐data or "macro data" or macrodata or "mass data" or massdata or "large data" or "bulk data")

# 2 45,418

TS= (((satellite* NEAR/3 imag*) or sensor* or surveillance or meter* or drone* or ((mobile* or cell) NEAR/3 (phone* or telephone*)) or mobiles or gps or "global positioning" or gis or "global information system*" or self‐tracking or "self tracking") NEAR/3 data)

# 3 41,524

TS= (((e‐commerce or commerc* or business* or "credit card*" or ATM* or "automated teller" or "cash machine*" or "money transfer*") and (transaction* or record or records)) or ((savings or loan or loans) NEAR/3 repay*) NEAR/3 data)

# 4 109

TS= (((((toll or tolls) NEAR/2 (road* or highway* or motorway*)) or "public transport") NEAR/3 data))

# 5 1,036

TS= ((((online or internet or web or virtual or google) NEAR/3 (search* or log or logs)) NEAR/3 data))

# 6 14,088

TS= ((("social network*" or "social media" or "opinion platform*" or blog* or Twitter or Facebook or LinkedIn or YouTube or Wiki* or Open or "text messag*") NEAR/3 data))

# 7 596

TS= ((((citizen* NEAR/2 (report* or derived or submit* or communicat* or inform or informed or informing or feedback)) or hotline* or crowdsourc*) NEAR/3 data))

# 8 133,635

#7 OR #6 OR #5 OR #4 OR #3 OR #2 OR #1

# 9 35,994

TS= (((poverty NEAR/5 (eradicat* or measur* or map*)) or ((wealth or GDP) NEAR/3 measur*) or (access* NEAR/5 (service* or financ*)) or "social protection" or (reduc* NEAR/3 vulnerab*)))

# 10 36,506

TS= ((((hunger or malnutrition or malnourished) NEAR/3 (eradicat* or decreas* or end*)) or "food security" or (improv* NEAR/3 nutrition*) or "sustain* agriculture" or (access* NEAR/3 food*) or ((productiv* or income*) NEAR/3 ("small scale" NEAR/2 produc*)) or (sustain* NEAR/3 "food produc*") or ((maintain* or maintenance) NEAR/2 "genetic diversity")))

# 11 57,651

TS= ((((health or well‐being or wellbeing or "well being") NEAR/2 (promot* or ensur*)) or (reduc* NEAR/3 (maternal or child* or "under 5*" or under‐5* or "under five*") NEAR/2 (mortality or death*)) or (("communicable disease*" or "infectious disease*") NEAR/3 (epidemic* or pandemic*)) or (reduc* NEAR/2 ("premature mortality" or "premature death*")) or ((prevent* or treatment) NEAR/3 "substance abuse") or ((reduc* or prevent*) NEAR/3 (road* or traffic or vehicle*) NEAR/2 (accident* or death* or fatal*)) or (access* NEAR/2 (reproductive or sexual) NEAR/2 service*) or (universal NEAR/2 health NEAR/2 coverage) or (pollut* NEAR/3 (death* or mortality or fatal*) NEAR/2 reduc*)))

# 12 26,746

TS= (((education or "life‐long learning" or (skill* NEAR/3 (build* or acquir* or learn*)) or literacy or numeracy) NEAR/3 (access* or complet* or graduat* or gender* or opportunit*)))

# 13 40,719

TS= (((gender* or women or girl* or female*) NEAR/3 (equalit* or inequalit* or discriminat* or empower* or violen* or harm* or (unpaid NEAR/2 (domestic or house*)) or participat* or ((sexual or reproductive) NEAR/3 right*))))

# 14 100,434

TS= (((water or WASH or sanitation) NEAR/3 (access* or quality or sustainable or manag* or ecosystem*)))

# 15 139,810

TS= (((energy NEAR/3 (access* or renewable or afford* or clean or sustainable or efficien*))))

# 16 210,410

TS= ((((econom* NEAR/2 (growth or productivity)) or employment or labour or work or job or jobs or development or "global resource*" or ((youth or "young people") NEAR/3 unemploy*) or tourism or (financ* NEAR/3 institution*) or bank or banks or banking) NEAR/3 (sustain* or increas* or promot* or efficien* or reduc* or forced or underage or "under age" or rights or safe or strengthen* or (capacity NEAR/2 build*))))

# 17 19,679

TS= (((industr* or innovation or infrastructure or (financ* NEAR/2 service*) or ((scien* or techn*) NEAR/2 research)) NEAR/3 (resilen* or sustain* or promot* or foster* or access* or upgrad* or modern*)))

# 18 97,676

TS= (((inequalit* NEAR/2 (reduc*)) or ((income NEAR/2 growth) or "equal opportunit*") NEAR/2 (sustain* or increas* or promot*)) or (inclusivity NEAR/2 (promot* or sustain* or empower*)) or (equality NEAR/2 (increas* or promot* or engender*)) or ((global NEAR/2 (financ* or market* or institution*) NEAR/3 (regulat* or monitor* or legislat* or law or laws)) or (("developing countr*" or LMIC* or "low and middle‐income" or "third world" or "global south") NEAR/3 (represent* NEAR/3 (enhanc* or ensur* or increas* or promot*)) or (migration NEAR/3 (policy or policies) NEAR/3 plan*) or (different* or special or positive) NEAR/2 (treat* or discriminat*)))

# 19 36,884

TS= (((city or cities or urban* or communit* or village* or housing or dwelling* or transport* or ((cultural or natural) NEAR/3 heritage) or ((disaster* or emergenc*) NEAR/3 manag*) or "green space*") NEAR/3 (sustain* or safe or inclusive or resilien* or afford* or protect* or ((reduc* or limit* or mitigat*) NEAR/3 "environmental impact" NEAR/3 (city or cities or urban*) or access*))))

# 20 149,994

TS= (((consum* or production or "natural resource*" or waste or wastes or procurement or (information NEAR/2 (access* or availab*))) NEAR/3 (sustain* or responsible or efficien* or environment* or reduc*)))

# 21 201,412

TS=(("climat* change*" or "global warming" or (greenhouse NEAR/2 (effect* or gas*)) NEAR/3 (action or combat* or mitigat* or impact* or resilien* or adapt* or (national NEAR/2 (policy or policies)) or ((promot* or rais* or improv*) NEAR/3 aware*))))

# 22 54,354

TS= (((ocean* or sea or seas or marine or coastal or fishing or fisher* or "small island*") NEAR/3 (sustain* or conserv* or manag* or protect* or regulat* or ecosystem* or (reduc* NEAR/2 pollut*) or acidity)))

# 23 22,000

TS= (((terrestrial or land or land‐based or forest* or mountain* or (natural NEAR/2 (habitat or ecosystem*)) or poaching or poacher* or traffick*) NEAR/3 (sustain* or conserv* or protect* or (degrad* NEAR/3 (neutral or prevent* or protect* or minimi* or reduc*)) or ("genetic resources" NEAR/2 (utilis or utiliz* or use or using or promot*)) or prohibit* or ban or banning or banned) or (("alien species" NEAR/2 (invasive or introduced or imported)) NEAR/3 (prevent* or impact*)) or ((ecosystem* or biodiversity) NEAR/3 (national or local*) NEAR/3 (plan* or policy or policies))))

# 24 2,378

TS=(((societ* NEAR/3 (peaceful or inclusive)) or justice or "rule of law") NEAR/3 (sustain* or access* or effective* or accountab* or strong or robust or promot*))

# 25 105,185

TS=((violen* or corrupt* or ((illicit or illegal) NEAR/2 financ* NEAR/2 (activit* or dealing*)) NEAR/2 (reduc* or mitigat* or eliminat*)) or (institution* NEAR/2 (accountab* or transparen* or effective*)))

# 26 17,951

TS=(((("decision making" or decision‐making or participati*) NEAR/2 (representative or democra*)) or (("global governance" NEAR/3 (participati* or involv* or includ*)) NEAR/3 ("developing countr*" or LMIC* or "low and middle‐income" or "global south" or "third world")) or ("legal identit*" NEAR/3 (provi* or promot* or regist*)) or (information NEAR/3 access*)))

# 27 141

TS= (("sustainable development goal*" NEAR/3 (partnership* or implement* or revitali* or strengthen* or sustainability or (capacity NEAR/2 build*) or ((development or financial or domestic) NEAR/2 (resource* or assistance)) or ((investment or technolog* or ("trading system*" NEAR/2 universal)) NEAR/3 (promot* or innovation*)) or (knowledge NEAR/2 shar*) or "technology bank*" or ((exports or market*) NEAR/3 (increas* or promot*) NEAR/3 ("developing countr*" or LMIC* or "low and middle‐income" or "third world" or "global south")) or "global macroeconomic" or ((policy or policies) NEAR/2 coheren*) or "global partnership*" or "public‐private partnership*" or (progress NEAR/3 (monitor* or assess* or evaluat* or review*)))))

#27 OR #26 OR #25 OR #24 OR #23 OR #22 OR #21 OR #20 OR #19 OR #18 OR #17 OR #16 OR #15 OR #14 OR #13 OR #12 OR #11 OR #10 OR #9

# 28 1,196,499

#28 AND #8

# 29 16,624

# 30 4,365

TS=(sdg or sdgs or "sustainable development goal*")

#30 AND #1

# 31 35

#31 OR #29

# 32 **16,625** (Indexes=SCI‐EXPANDED, SSCI Timespan=2005‐2019)

#28 AND #8

# 33 **3,519** (Indexes=SSCI Timespan=2005‐2019)

## APPENDIX E DATA EXTRACTION TOOL

**Table E1:** Data extraction tool

Variable name	Variable description
Study ID	Unique ID ascribed to each record
Title name	Use only the English version of the publication's main title. If paper is not written in English and has the title translated, use the translated version of the title. If the publication does not provide an English version, include the title in its original language. Please enter title in sentence case. Ensure there are no line breaks
Language	Select full‐text language that applies: English
Open access	If the study's (full‐text) content is available, code as “Yes.” If study has paywalls, code as “No”
	Please save the PDF in the Dropbox folder called “Full‐Text PDFs” using the following format Firstauthorsurname_year_record id
	If study has multiple versions, in other words, if the study has been published as both a journal article and a working paper, both versions may be included in the IER
**Type of big data**	Select one or more from the list based on the type of big data being used:
	1. Human‐sourced information (social networks) 11. Social networks 12. Internet searches 13. Mobile data content 14. Citizen reporting or crowdsourced data 2. Process‐mediated data (traditional business systems and websites) 21. Data produced by public agencies 22. Data produced by businesses 23. Mobile phone CRD 3. Machine‐generated data (automated systems) 31. Data from fixed sensors 32. Data from mobile sensors (tracking) 33. Data from satellites
**Outcome**	Select ONE outcome that applies according to the intervention being evaluated:
	Economic development and livelihood
	Sustainable agriculture and food security
	Health and well‐being
	Education
	Water and sanitation
	Governance and human rights
	Energy, industry and infrastructure
	Urban development
	Environment sustainability
	Global partnership
**Suboutcomes**	Poverty mapping/measurement
	Measuring wealth/GDP
	Access to basic services
	Social protection, including financial inclusion
	Vulnerability reduction
	Economic growth and productivity
	Full and productive employment, including youth unemployment
	Eradicate forced and underage labour, protect labour rights and promote safe workspace
	Promote sustainable tourism
	Strengthen capacity of domestic financial institutions
	Reduce mortality
	End epidemics of communicable diseases
	Strengthen prevention and treatment of substance abuse
	Decrease fatalities due to road accidents
	Universal access to sexual and reproductive healthcare services
	Achieve universal health coverage
	Reduce fatalities due to pollution
	Achieve gender equality and empower all women and girls
	Reduce inequality within and among countries
	Promote peaceful and inclusive societies for sustainable development, provide access to justice for all and build effective, accountable and inclusive institutions at all levels
	Access to affordable housing
	Access to affordable transport systems
	Enhance inclusive and sustainable urbanisation
	Strengthen efforts to protect cultural and natural heritage
	Better disaster management
	Reduce environmental impact of cities
	Universal access to green public spaces
	Ensure sustainable consumption and production patterns
	Take urgent action to combat climate change and its impacts
	Conserve and sustainable use the oceans, seas and marine resources for sustainable development
	Protect, restore and promote sustainable use of terrestrial ecosystems, sustainably manage forests, combat desertification, halt and reverse land degradation and halt biodiversity loss
**Gender** equity focus	Does this study consider gender and/or* equity?
	Yes
	No
**Cost**	Is cost data provided?
	Yes
	No
**Population**	What population sub‐groups does the study target?
	Rural
	Urban
	Refugees
	Conflict affected persons
	Ethnic minorities
	Sex
**Geographical coverage**	Select the continent/region in which the study was conducted:
	Asia
	Africa
	North America
	South America
	Europe
	Australia
	Antarctica
**Fragile context**	Select the type based on fragile context definition
	a. Conflict related difficulty b. Logistical difficulty c. All others
Evaluation design	Select one of three options defined as:
	1. Experimental
	a) RCT defined as prospective randomised assignment, where randomisation is implemented by researchers (or by decision makers in the context of an evaluation study)
	2. Quasi‐experimental
	a) Quasi‐random assignment: i) regression discontinuity design (sharp designs) or ii) natural experiment in which exposure to treatment is random b) b) Nonrandom assignment: i) studies that control for unobservables (DID, FE, IV, Fuzzy RDD, ITS) or ii) studies that control for observables only (e.g., statistical matching, synth control, regression adjustment)
	3. Measurement studies
	Studies that have innovatively used big data to measure and validate an SDG indicator such as poverty mapping, food security, forest cover, and so forth
Evaluation method	If experimental, then select (only if one or two selected under evaluation design):
	RCTs
	If quasi‐experimental, then select:
	Sharp RDD
	DID
	FE estimation
	IV estimation
	Fuzzy RDD
	Statistical matching (includes PSM)
Methods for analysing big data	Select one or more methods used in the analysis of “big data”:
	ML: supervised learning, unsupervised learning, clustering, anomaly detection, random forests, artificial neural network, convolutional neural network, support vector regression
	Bayesian geostatistical models: latent Gaussian models, integrated nested Laplace approximations
	Natural language processing: vectorisation, embedding, categorisation, sentiment analysis
	Any other: please specify
**Big data validation**	Select one or more methods used to calibrate/validate results of big data analysis using survey data:
	Administrative/survey data used for calibration or validation? Yes/No
	Type of data
	Administrative data Survey data a. Secondary survey data b. Primary survey data
	Survey name
	Demographic Health Survey Living Standards Measurement Study Other (specify)
	Mode of data collection
	Face‐to‐face Telephone mode Mobile phone telephone mode Dual‐frame Other (specify)
	Sample size
	Sample design
	Link to survey details (if available)
Mixed methods	Select YES if study includes quantitative and qualitative analyses, otherwise select NO
**Transparency in data collection, analysis and reporting**	Choose if the study discusses the following:
	Are the data collection methods described? Are data quality issues such as completeness (missing or incomplete entries; empty cells) and noise discussed? Is the data representative of the population of interest? Is the construct validity explained (ie is there a discussion on how the big data‐based indicator measures what the study claims to measure)? Are the results generalisable? For example, are the research findings generalisable to other situations such as other platforms (data source) or communities, or over time? Are data and codes publicly available for replication? Any other data, analysis and reporting challenge discussed?
**Ethical approval and discussion**	Choose if the study discusses the following:
	Ethical approval obtained? Consent for secondary and other use of data Data privacy Data security and governance arrangement Unintended exclusion Unintended consequence on any group of people and/or individuals Others (specify)
Unit of observation	Enter all the levels of observation of the variables used for the analysis:
	Community Cohort (includes schools or clinics) Household Individual
	If more than one, include in separate rows:
	Country Districts Sub‐districts Village/city
**Funding agency**	What category of funding agency funded the research?
	*Note: only code if reported in the study; no need to do additional research to find*
	Select one of the following:
	Government agency International aid agency International financial institution Nonprofit organisation For‐profit firm Academic institution Charitable foundation or private foundation Not specified

## APPENDIX F ML AND MANUAL SCREENING

All the studies obtained from manual and automatic search were imported to EPPI‐Reviewer 4, a software facilitating management of references, identification and removal of duplicates, and screening of studies at both title and abstract and full‐text stages. The title and abstract screening was done using the ML functionality available in EPPI in order to make the process more efficient. The model built using this functionality learns from the set of manually screened studies to guess the inclusion criteria and reorganises the list of studies based on the likelihood of being included. Using a training dataset consisting of 3300 manually screened studies, the algorithm ranked studies by prioritising them in order of likelihood of inclusion and excluding more than 1500 studies in a row. Based on this likelihood, the studies are grouped into 10 groups (Figure A6), with the set of excluded studies classified in the 0–10% range.

This systematic map includes studies that are extremely diverse; hence ML may not be accurate. To undertake a more nuanced approach and to account for this diversity in studies we conducted an additional manual screening within the groups. Since this manual screening gave us only 5% includes from the set of studies within 10–40% range, those were not considered in the subsequent screening process. Once we had a set of studies that were included in the title and abstract screening stage, this was followed by full‐text screening. Here, we screened studies that had more than a 50% likelihood of inclusion based on the ML model. A random check on 20% of studies belonging to the 40–49% group provided a very limited number of relevant studies that could be included, due to which the lower likelihood studies were not screened.

## APPENDIX G SR APPRAISAL TOOL AND SUMMARY

### G.1. Summary of the SRs

#### Yan et al. (2017). Utility and potential of rapid epidemic intelligence from internet‐based sources

The study aimed to summarise internet‐based methods that use freely accessible and unstructured data for epidemic surveillance, exploring their timeliness and accuracy outcomes. The study is based on 84 articles published between 2006 and 2016 relating to internet‐based public health surveillance methods. These studies employ search queries, social media posts and approached derived from existing internet‐based systems for early epidemic alerts and real‐time monitoring. The primary methodology used for this review is the preferred reporting items for SR and meta‐analyses. Using this method, the authors can assess the benefits and challenges of a healthcare intervention through an evidence‐based minimum set of items. The study does not clearly demonstrate the inclusion criteria for the study designs, making it difficult for the readers to interpret the findings.

#### Fung et al. (2016). Ebola virus disease and social media: A systematic review

The study is an SR of the existing research pertinent to the Ebola virus and social media, especially to identify the research questions and methods used to collect and analyse social media. The study searched six databases for research articles relevant to Ebola and social media. Twelve articles were included in the main analysis: seven from Twitter and one including Weibo, one from Facebook, three from YouTube and one from Instagram and Flickr. All the studies were cross‐sectional. The study uses a standardised form to extract the data. A key challenge of the review is that the methods used by the review authors to analyse the findings of the included studies are not clearly defined.

#### Williamson et al. (2019). Satellite remote sensing in shark and ray ecology, conservation and management

The study aims to present a summary of the existing state of knowledge on the application of satellite remote sensing (SRS). SRS is seen as a key opportunity to analyse important environmental drivers in elasmobranch ecology and to aid management decisions for the conservation of declining population. The review included 71 papers and made use of several academic tools in order to conduct the bibliographic search. These included ISI Web of Science, Scopus and Google Scholar. One of the limitations of the review is that it examines a wide variety of studies but does not combine the results of these studies explicitly.

#### Mehta and Pandit (2018). Concurrence of big data analytics and healthcare: A systematic review

This SR of literature aims to determine the scope of big data analytics in the field of healthcare including its applications and challenges in its adoption in healthcare. The review employs a systematic search of articles on five major scientific databases: Science Direct, PubMed, Emerald, IEEE Xplore and Taylor & Francis. Two reviewers independently extracted information on the definition of big data analytics and the sources and applications of big data. A total of 58 articles were selected. The authors did not explicitly state the methods used to analyse the quality of included studies and also did not report results for each of the studies in the review.

#### De Souza et al. (2019). Data mining and machine learning to promote smart cities: A systematic review from 2000 to 2018

This study aimed to present an SR regarding data mining (DM) and machine learning (ML) approaches adopted in the promotion of smart cities. The study seeks to provide, from a literature review in journals belonging to the Web of Science and Scopus databases, the different DM and ML techniques used, as well as to present the sectors most engaged in the promotion of smart cities. A total of 39 studies were included on the map, which were further analysed to assess the most commonly used DM and ML techniques to promote smart cities. While the report describes individual results of the studies clearly, it does not combine the results of all the studies.

#### Charles‐Smith et al. (2015). Using social media for actionable disease surveillance and outbreak management: A systematic literature review

The studies in this review demonstrate how social media may be a valuable tool in improving the ability of public health professionals to detect disease outbreaks faster than by using traditional methods and to enhance outbreak response. A social media application was defined for this review as “an internet‐based application where people can communicate and share resources and information, and where users can activate and set their own profiles, have the ability to develop and update them constantly and have the opportunity to make such profiles totally or partially public and linked with other profiles in the network.” A total of 60 articles were selected for this SR, which addressed the two research questions: can social media be integrated into disease surveillance and can it be used to improve health outcomes?

#### Krenn et al. (2011). Use of global positioning systems (GPSs) to study physical activity and the environment: a systematic review

The aims of this SR were to determine the capability of GPS to collect high‐quality data on the location of activities in research on the relationship between physical activity and the environment. Studies were eligible for inclusion if they were undertaken on humans, used GPS to measure the locations where physical activity occurred and included analysis of the relationship between the characteristics of the environment and any form of physical activity behaviour (including leisure‐time physical activity, sport or active travel). The capability of GPS was expressed in terms of data quality, which in turn was defined as the proportion of GPS data lost in each study. The authors do not mention the proper risk of bias assessment of the included study.

#### Bennett and Smith (2017). Advances in using multitemporal night‐time lights satellite imagery to detect and estimate and monitor socioeconomic dynamics

This paper reviews progress in using the multitemporal Defense Meteorological Satellite Program‐Operational Linescan System, for which digital imagery was available from 1992 to 2013. It also reviews Visible Infraref Imaging Radiometer imagery to analyse urbanisation, economic and population dynamics across range of geographic scales. The review provides an overview of data corrections and processing for a comparison of multitemporal nightlight imagery. The review includes a total of 17 studies. The results of all the studies were not properly combined and reported in the review. A risk of bias assessment of the included study has also not been reported in the review.

**Table G1:** SR appraisal

Questions	Yan et al. (2017)	Fung et al. (2016)	Williamson et al. (2019)	Mehta and Pandit (2018)	De Souza et al. (2019)	Charles‐Smith et al. (2015)	Krenn et al. (2011)	Benett and Smith (2017)
**Section A: Methods used to identify, include and critically appraise studies**								
A.1 Were the criteria used for deciding which studies to include in the review reported?	Yes	Yes	Yes	Partially	Yes	Yes	Yes	Yes
A.2 Was the search for evidence reasonably comprehensive?	No	No	Yes	No	Partially	No	Partially	No
A.3 Does the review cover an appropriate period?	Not clear	Not clear	Not clear	Not clear	Not clear	Not clear	Not clear	Not clear
A.4 Was bias in the selection of articles avoided?	No	Partially	No	No	Partially	No	No	Not clear
A.5 Did the authors use appropriate criteria to assess the quality and risk of bias in analysing the studies that are included?	No	Not clear	Not clear	No	No	No	No	No
A.6 Overall, how much confidence do you have in the methods used to identify, include and critically appraise studies?	Low (limitations are important enough that the results of the review are not reliable)	Low (limitations are important enough that the results of the review are not reliable)	Low (limitations are important enough that the results of the review are not reliable)	Low (limitations are important enough that the results of the review are not reliable)	Low (limitations are important enough that the results of the review are not reliable)	Low (limitations are important enough that the results of the review are not reliable)	Low (limitations are important enough that the results of the review are not reliable)	Low (limitations are important enough that the results of the review are not reliable)
**Section B Methods used to analyse the findings**								
B.1 Were the characteristics and results of the included studies reliably reported?	Yes	No	No	Partially	No	No	No	Partially
B.2 Are the methods used by the review authors to analyse the findings of the included studies clear, including methods for calculating effect sizes (if applicable)?	No	Yes	No	No	Yes	No	No	No
B.3 Did the review describe the extent of heterogeneity?	No	No	No	No	Yes	Yes	No	No
B.4 Were the findings of the relevant studies combined (or not combined) appropriately relative to the primary question the review addresses and the available data?	Yes	Yes	No	No	Partially	No	No	Partially
B.5 Does the review report evidence appropriately?	No	No	No	Partially	No	No	Yes	Yes
B.6 Did the review examine the extent to which specific factors might explain differences in the results of the included studies?	No	No	No	No	No	No	No	No
B.7 Overall, how much confidence do you have in the methods used to analyse the findings relative to the primary question addressed in the review?	Low (limitations are important enough that the results of the review are not reliable)	Low (limitations are important enough that the results of the review are not reliable)	Low (limitations are important enough that the results of the review are not reliable)	Low (limitations are important enough that the results of the review are not reliable)	Low (limitations are important enough that the results of the review are not reliable)	Low (limitations are important enough that the results of the review are not reliable)	Low (limitations are important enough that the results of the review are not reliable)	Low (limitations are important enough that the results of the review are not reliable)
**Section C: Overall assessment of the reliability of the review**								
C.1 Are there any other aspects of the review not mentioned before that lead you to question the results?	No	No	No	No	No	No	No	No
C.2 Are there any mitigating factors that should be taken into account in determining the review's reliability?	Not clear	Not clear	Not clear	Not clear	Not clear	Not clear	Not clear	Can't tell
C.3 Based on the above assessments of the methods, how would you rate the reliability of the review?	Low (limitations are important enough that the results of the review are not reliable)	Low (limitations are important enough that the results of the review are not reliable)	Low (limitations are important enough that the results of the review are not reliable)	Low (limitations are important enough that the results of the review are not reliable)	Low (limitations are important enough that the results of the review are not reliable)	Low (limitations are important enough that the results of the review are not reliable)	Low (limitations are important enough that the results of the review are not reliable)	Low (limitations are important enough that the results of the review are not reliable)

## APPENDIX H ADDITIONAL TABLES AND FIGURES

**Table H1:** Top 20 countries with maximum number of studies

Country	Total	IE	Measurement study	SRs
China	43	6	37
India	34	4	30	
United States	31	3	28
Multicountry	31	3	25	3
Unidentified	14	1	11	2
Kenya	13	13		
Brazil	11	3	8
Mexico	10	5	5	
Ethiopia	9	1	8
Bangladesh	8	1	7	
Indonesia	8	1	7
Rwanda	8	2	6	
Uganda	8	2	6	
Japan	7	7		
Nigeria	7	7		
Tanzania	7	1	6	
Iran	6	6		
Sri Lanka	6	6		

**Table H2:** Distribution of studies across the region

	East Asia and Pacific	Europe and Central Asia	Latin America and the Caribbean	Middle East and North Africa	North America	South Asia	Sub‐Saharan Africa
IE	12	5	18	3	5	11	14
Measurement study	92	69	42	29	49	82	112
SRs	7	6	5	6	6	6	6
Total	111	80	65	38	60	99	132

**Table H3:** Studies using multiple sources of big data

Combination of big data	IEs	Measurement study	SRs	Grand total
Mobile phone CRD || Data from fixed sensors		1		1
Mobile phone CRD || Data from satellites		10		10
Citizen reporting and crowdsourced data || Data from fixed sensors || Data from satellites		1		1
Citizen reporting and crowdsourced data || Data from mobile sensors (tracking)		1		1
Citizen reporting and crowdsourced data || Data from satellites		1		1
Data from fixed sensors || Data from satellites	2	13		15
Data from mobile sensors (tracking) || Data from satellites		5		5
Data produced by public agencies || Data from fixed sensors || Data from satellites	1	1		2
Data produced by public agencies || Data from satellites	1	3		4
Data produced by public agencies || Data produced by businesses		1		1
Mobile data content || Mobile phone CRD		2		2
Mobile data content || Citizen reporting and crowdsourced data		2		2
Mobile data content || Data from mobile sensors (tracking)		1		1
Social networks || Citizen reporting and crowdsourced data		2		2
Social networks || Data from satellites		1		1
Social networks || Data produced by public agencies || Data from fixed sensors || Data from satellites		1		1
Social networks || Internet searches		4	1	5
Social networks || Internet searches || Mobile data content || Mobile phone CRD		1		1
Social networks || Mobile data content || Mobile phone CRD		1		1
**Grand total**	**4**	**52**	**1**	**57**

**Table H4:** Big data source wise studies in fragile context

	Chemical or radio‐nuclear	Conflict or humanitarian crisis	Difficult terrain	Disease outbreaks or epidemics	Natural disaster	Others	Total (row)
Mobile phone CRD	1	6	0	11	10	0	28
Citizen reporting and crowdsourced data	0	7	1	0	1	0	9
Data from fixed sensors	0	5	7	0	1	1	14
Data from mobile sensors (tracking)	0	0	2	0	0	0	2
Data from satellites	0	25	18	4	10	3	60
Data produced by businesses	0	0	0	0	0	0	0
Data produced by public agencies	0	0	0	1	3	0	4
Internet searches	0	0	0	1	0	1	2
Mobile data content	0	1	0	5	0	0	6
Social networks	0	2	0	2	0	1	5
Total (column)	1	46	28	24	25	6

**Table H5:** How studies have dealt with data quality and transparency

	Data collection methods	Data quality issues	Data representative of the population of interest	Construct validity explained	Results are generalisable	Data and code publicly available	Data, analysis and reporting challenges discussed
IE	47	22	29	43	26	11	6
Measurement study	43	3	7	21	4	8	11
SRs	1	0	0	0	0	0	1
Grand total	91	25	36	64	30	19	18

## References

[cl21149-bib-0001] Alam, M. , Dappe, M.H. , Melecky, M. , & Goldblatt, R. (2019). *Wider economic benefits of transport corridors: Evidence from International Development Organizations*. Policy Research Working Papers. The World Bank. 10.1596/1813-9450-9057

[cl21149-bib-0002] Ali, D.A. , Deininger, K. , & Monchuk, D. (2018). *Using satellite imagery to assess impacts of soil and water conservation measures: Evidence from Ethiopia's Tana–Beles watershed*. Policy Research Working Papers. The World Bank. 10.1596/1813-9450-8321

[cl21149-bib-0003] Alix‐Garcia, J. , Aronson, G. , Radeloff, V. , Ramirez‐Reyes, C. , Shapiro, E. , Sims, K. , & Yanez‐Pagans, P. (2015). Impacts of payments for ecosystem services programme in Mexico. 3ie Impact Evaluation Report 20, 2014, New Delhi: International Initiative for Impact Evaluation (3ie). Available at. https://bit.ly/2UH1emH

[cl21149-bib-0004] Alix‐Garcia, J. M. , Shapiro, E. N. , & Sims, K. R. (2012). Forest conservation and slippage: Evidence from Mexico's national payments for ecosystem services program. Land Economics, 88(4), 613–638.

[cl21149-bib-0005] Allcott, H. (2011). Social norms and energy conservation. Journal of Public Economics, 95, 1082–1095. 10.1016/j.jpubeco.2011.03.003

[cl21149-bib-0006] Allcott, H. , & Rogers, T. (2014). The short‐run and long‐run effects of behavioral interventions: Experimental evidence from energy conservation. American Economic Review, 104, 3003–3037. 10.1257/aer.104.10.3003

[cl21149-bib-0007] Anderson, L.O. , De Martino, S. , Harding, T. , Kuralbayeva, K. , & Lima, A. (2016). *The effects of land use regulation on deforestation: Evidence from the Brazilian Amazon*. OxCarre Working Papers. Oxford Centre for the Analysis of Resource Rich Economies, University of Oxford.

[cl21149-bib-0008] Asher, S. , Garg, T. , & Novosad, P. (2018). *The ecological impact of transportation infrastructure*. Policy Research Working Papers. The World Bank. 10.1596/1813-9450-8507

[cl21149-bib-0009] Ayres, I. , Raseman, S. , & Shih, A. (2012). Evidence from two large field experiments that peer comparison feedback can reduce residential energy usage. The Journal of Law, Economics, and Organization, 29, 992–1022. 10.1093/jleo/ews020

[cl21149-bib-0498] Bamberger, M. (2012). Introduction to mixed methods in impact evaluation. Impact Evaluation Notes, 3(3), 1–38.

[cl21149-bib-0010] BenYishay, A. , Glenn, C. , Runfola, D. , Goodman, S. , & Trichler, R. (2018). Final Report: Evaluation of the on‐farm water management program. Williamsburg, VA: AidData at William & Mary.

[cl21149-bib-0011] BenYishay, A. , Parks, B. , Runfola, D. , & Trichler, R. (2016). *Forest cover impacts of chinese development projects in ecologically sensitive areas*. AidData Working Paper #32. Williamsburg, VA: AidData at William & Mary.

[cl21149-bib-0601] BenYishay, A. , Runfola, D. , Trichler, R. , Dolan, C. , Goodman, S. , Parks, B. , & Anand, A. (2017). A primer on geospatial impact evaluation methods, tools, and applications. In AidData Working Paper# 44. AidData at William & Mary Williamsburg, VA.

[cl21149-bib-0012] BenYishay, A. , Trichler, R. , Runfola, D. , & Goodman, S. (2018b). Evaluation of the infrastructure needs program II. Williamsburg, VA: AidData at William & Mary.

[cl21149-bib-0013] BenYishay, A. , Trichler, R. , Runfola, D. , & Heuser, S. (2016b). *Indigenous land rights and deforestation: evidence from the Brazilian Amazon*. AidData Working Paper #22. Williamsburg, VA: AidData at William & Mary.

[cl21149-bib-0014] Blackman, A. (2015). Strict versus mixed‐use protected areas: Guatemala's Maya Biosphere Reserve. Ecological Economics, 112, 14–24. 10.1016/j.ecolecon.2015.01.009

[cl21149-bib-0015] Blackman, A. , Pfaff, A. , & Robalino, J. (2015). Paper park performance: Mexico's natural protected areas in the 1990s. Global Environmental Change, 31, 50–61. 10.1016/j.gloenvcha.2014.12.004

[cl21149-bib-0016] Blackman, A. , & Villalobos, L. (2019). Clear, but don't invest: Protected areas discourage some land uses more than others. Environmental Research Letters, 14. 10.1088/1748-9326/ab3ca1

[cl21149-bib-0017] Blumenstock, J. E. , Callen, M. , Ghani, T. , & Koepke, L. (2015, May). Promises and pitfalls of mobile money in Afghanistan: Evidence from a randomized control trial. *Proceedings of the Seventh International Conference on Information and Communication Technologies and Development* (pp. 1–10).

[cl21149-bib-0018] Blumenstock, J. , Eagle, N. , & Fafchamps, M. (2011). *Risk and reciprocity over the mobile phone network: Evidence from Rwanda*. CSAE Working Paper Series. Centre for the Study of African Economies, University of Oxford.

[cl21149-bib-0019] Bonnier, E. , Poulsen, J. , Rogall, T. , & Stryjan, M. (2016). *Preparing for genocide: Quasi‐experimental evidence from Rwanda*. SITE Working Paper Series. Stockholm School of Economics, Stockholm Institute of Transition Economics.

[cl21149-bib-0020] Buchanan, G. M. , Parks, B. C. , Donald, P. F. , O'Donell, B. F. , Runfola, D. , Swaddle, J. P. , Tracewski, L. , & Butchart, S. H. M. (2015). *The impacts of World Bank development projects on sites of high biodiversity importance*. AidData Work. Paper Series.

[cl21149-bib-0021] Buchanan, G. M. , Parks, B. C. , Donald, P. F. , O'Donnell, B. F. , Runfola, D. , Swaddle, J. P. , Tracewski, Ł. , & Butchart Stuart, H. M. (2018). The local impacts of World Bank development projects near sites of conservation significance. Journal of Environment and Development, 27, 299–322. 10.1177/1070496518785943

[cl21149-bib-0022] Buntaine, M. T. , Hamilton, S. E. , & Millones, M. (2015). Titling community land to prevent deforestation: An evaluation of a best‐case program in Morona‐Santiago, Ecuador. Global Environmental Change, 33, 32–43. 10.1016/j.gloenvcha.2015.04.001

[cl21149-bib-0023] Bunte, J. B. , Desai, H. , Gbala, K. , Parks, B. , & Runfola, D. M. (2017). Natural resource sector FDI and growth in post‐conflict settings: Subnational evidence from Liberia. Williamsburg (VA): AidData.

[cl21149-bib-0024] Burwen, J. , & Levine, D. I. (2012). A rapid assessment randomized‐controlled trial of improved cook stoves in the Tumu Region of Ghana. Energy for Sustainable Development, 16(3), 328–338.

[cl21149-bib-0025] Chen, Y. , Ebenstein, A. , Greenstone, M. , & Li, H. (2013). Evidence on the impact of sustained exposure to air pollution on life expectancy from China's Huai River policy. Proceedings of the National Academy of Sciences of the United States of America, 110, 12936–12941. 10.1073/pnas.1300018110 23836630 PMC3740827

[cl21149-bib-0026] Corbi, R. , Papaioannou, E. , & Surico, P. (2014). *Federal transfer multipliers: Quasi‐experimental evidence*. NBER Working Paper, No. 20751.

[cl21149-bib-0495] Desouza, K. C. , & Jacob, B. (2017). Big data in the public sector: Lessons for practitioners and scholars. Administration & Society, 49(7), 1043–1064.

[cl21149-bib-0027] Gaveau, D. L. A. , Epting, J. , Lyne, O. , Linkie, M. , Kumara, I. , Kanninen, M. , & Leader‐Williams, N. (2009). Evaluating whether protected areas reduce tropical deforestation in Sumatra. Journal of Biogeography, 36, 2165–2175. 10.1111/j.1365-2699.2009.02147.x

[cl21149-bib-0028] Ibarra, G.L. , McKenzie, D. , & Ortega, C.R. (2017). *Learning the impact of financial education when take‐up is low*. Policy Research Working Papers. The World Bank. 10.1596/1813-9450-8238

[cl21149-bib-0029] Jain, M. , Balwinder‐Singh, R. P. , Srivastava, A. K. , Poonia, S. , Blesh, J. , Azzari, G. , McDonald, A. J. , & Lobell, D. B. (2019). The impact of agricultural interventions can be doubled by using satellite data. Nature Sustainability, 2, 931–934. 10.1038/s41893-019-0396-x

[cl21149-bib-0030] Jaiswal, S. , Bensch, G. , Navalkar, A. , Jayaraman, T. , Murari, K. , & Patnaik, U. (2020). Evaluating the impact of infrastructure development: Case study of the Konkan Railway in India. 3ie Impact Evaluation Report 114. New Delhi: International Initiative for Impact Evaluation (3ie). Available at. 10.23846/DPW1IE114

[cl21149-bib-0031] Jayachandran, S. , de Laat, J. , Lambin, E.F. , & Stanton, C.Y. (2016). *Cash for carbon: A randomized controlled trial of payments for ecosystem services to reduce deforestation*. National Bureau of Economic Research Working Paper Series No. 22378. 10.3386/w22378

[cl21149-bib-0497] Jerven, M. (2014). Benefits and costs of the data for development targets for the post‐2015 development agenda. Data for Development Assessment Paper, 16(9), 14.

[cl21149-bib-0032] Jianing, Z. , Runfola, D. , & Kemper, P. (2017). *Quantifying heterogeneous causal treatment effects in World Bank development finance projects*. In Joint European Conference on Machine Learning and Knowledge Discovery in Databases (pp. 204–215). Springer.

[cl21149-bib-0033] Li, S. , & Liu, Y. (2020). Using big data to evaluate the impacts of transportation infrastructure investment: The case of subway systems in Beijing. 3ie Impact Evaluation Report 115, New Delhi: International Initiative for Impact Evaluation (3ie). Available at. 10.23846/DPW1IE115

[cl21149-bib-0034] Li, S. , Liu, Y. , Purevjav, A.‐O. , & Yang, L. (2019). Does subway expansion improve air quality? Journal of Environmental Economics and Management, 96, 213–235. 10.1016/j.jeem.2019.05.005

[cl21149-bib-0035] Long, F. , Zheng, L. , & Song, Z. (2018). High‐speed rail and urban expansion: An empirical study using a time series of nightime light satellite data in China. Journal of Transport Geography, 72, 106–118.

[cl21149-bib-0496] Maaroof, A. (2015). Big data and the 2030 agenda for sustainable development. Report for UN‐ESCAP.

[cl21149-bib-0036] Marty, R. , Goodman, S. , LeFew, M. , Carrie, D. , BenYishay, A. , & Runfola, D. (2019). Assessing the causal impact of Chinese aid on vegetative land cover in Burundi and Rwanda under conditions of spatial imprecision. Development Engineering, 4, 100038. 10.1016/j.deveng.2018.11.001

[cl21149-bib-0501] OECD . (2018). States of Fragility. https://www.oecd.org/dac/conflict-fragility-resilience/docs/OECD%20Highlights%20documents_web.pdf

[cl21149-bib-0037] Pande, R. , & Sudarshan, A. (2019). Harnessing transparency initiatives to improve India's environmental clearance process for the mineral mining sector, 3ie Impact Evaluation Report 92. New Delhi: International Initiative for Impact Evaluation (3ie). 10.23846/TW8IE92

[cl21149-bib-0038] Parks, B. , Baehr, C. , Aboagye, D. , Trichler, R. , BenYishay, A. , Runfola, D. , & Puram, P. (2019). Building on a Foundation Stone: The Long‐Term Impacts of a Local Infrastructure and Governance Program in Cambodia. Stockholm: Swedish EBA, AidData, and Open Development Cambodia.

[cl21149-bib-0039] Pellegrini, L. (2019). Impacts of community monitoring of socio‐environmental liabilities in the Ecuadorian and Peruvian Amazon. 3ie Impact Evaluation Report 99. New Delhi: International Initiative for Impact Evaluation (3ie). 10.23846/TW8IE99

[cl21149-bib-0040] Persha, L. , & Meshack, C. (2016). *A triple win? The impact of Tanzania's Joint Forest Management programme on livelihoods, governance and forests*. 3ie Impact Evaluation Report 34, New Delhi: International Initiative for Impact Evaluation (3ie). https://bit.ly/391Au8K

[cl21149-bib-0041] Scullion, J. , Thomas, C. W. , Vogt, K. A. , Perez‐Maqueo, O. , & Logsdon, M. G. (2011). Evaluating the environmental impact of payments for ecosystem services in Coatepec (Mexico) using remote sensing and on‐site interviews. Environmental Conservation, 38, 426–434. 10.1017/S037689291100052X

[cl21149-bib-0042] Tanner, J. , Goodman, S. , Leu, M. , Trichler, R. , BenYishay, A. , Runfola, D. , Marty, R. , & Nagol, J. (2017). A top‐down approach to estimating spatially heterogeneous impacts of development aid on vegetative carbon sequestration. Sustainability, 2017(9), 409.

[cl21149-bib-0043] Van der Windt, P. , & Humphreys, M. (2016). Crowdseeding in Eastern Congo: Using cell phones to collect conflict events data in real time. Journal of Conflict Resolution, 60, 748–781.

[cl21149-bib-0044] Velilla, R. , & Braganca, A. (2019). Coffee price shock and local economic growth: Evidence from Colombia. https://bit.ly/2UQaGE5

[cl21149-bib-0045] Villa, J. M. (2016). Social transfers and growth: Evidence from luminosity data. Economic Development and Cultural Change, 65, 39–61. 10.1086/687548

[cl21149-bib-0046] Webster, J. , Landegger, J. , Bruce, J. , Malunda, J. , Chantler, T. , Kumakech, E. , Schmucker, L. , Kiapi, L. , Kozuki, N. , Olorunsaiye, C. , & Bryne, E. (2019). Impacts of IRC's Fifth Child community engagement strategy to increase immunisation in northern Uganda. 3ie Grantee Final Report. New Delhi: International Initiative for Impact Evaluation (3ie). https://bit.ly/391NX0q

[cl21149-bib-0047] Yang, J. , Chen, S. , Qin, P. , Lu, F. , & Liu, A. A. (2018). The effect of subway expansions on vehicle congestion: Evidence from Beijing. Journal of Environmental Economics and Management, 88, 114–133. 10.1016/j.jeem.2017.09.007

[cl21149-bib-0048] Yang, J. , Purevjav, A.‐O. , & Li, S. (2020). The marginal cost of traffic congestion and road pricing: Evidence from a natural experiment in Beijing. American Economic Journal: Economic Policy, 12, 418–453. 10.1257/pol.20170195

[cl21149-bib-0049] Bennett, M. M. , & Smith, L. C. (2017). Advances in using multitemporal night‐time lights satellite imagery to detect, estimate, and monitor socioeconomic dynamics. Remote Sensing of Environment, 192, 176–197. 10.1016/j.rse.2017.01.005

[cl21149-bib-0050] Charles‐Smith, L. E. , Reynolds, T. L. , Cameron, M. A. , Conway, M. , Lau, E. H. Y. , Olsen, J. M. , Pavlin, J. A. , Shigematsu, M. , Streichert, L. C. , Suda, K. J. , & Corley, C. D. (2015). Using social media for actionable disease surveillance and outbreak management: A systematic literature review. PLOS One, 10, e0139701. 10.1371/journal.pone.0139701 26437454 PMC4593536

[cl21149-bib-0051] De Souza, J. T. , de Francisco, A. C. , Piekarski, C. M. , & do Prado, G. F. (2019). Data mining and machine learning to promote smart cities: A systematic review from 2000 to 2018. Sustainability, 11, 1077. 10.3390/su11041077

[cl21149-bib-0052] Fung, C. H. , Duke, C. H. , Finch, K. C. , Snook, K. R. , Tseng, P. L. , Hernandez, A. C. , Manoj, G. , Fu, K. W. , & Tse, T. H. (2016). Ebola virus disease and social media: A systematic review. American Journal of Infection Control, 44, 1660–1671. 27425009 10.1016/j.ajic.2016.05.011

[cl21149-bib-0053] Krenn, P. J. , Titze, S. , Oja, P. , Jones, A. , & Ogilvie, D. (2011). Use of global positioning systems to study physical activity and the environment: A systematic review. American Journal of Preventive Medicine, 41, 508–515. 22011423 10.1016/j.amepre.2011.06.046 PMC3821057

[cl21149-bib-0054] Mehta, N. , & Pandit, A. (2018). Concurrence of big data analytics and healthcare: A systematic review. International Journal of Medical Informatics, 114, 57–65. 10.1016/j.ijmedinf.2018.03.013 29673604

[cl21149-bib-0055] Williamson, M. J. , Tebbs, E. J. , Dawson, T. P. , & Jacoby, D. M. P. (2019). Satellite remote sensing in shark and ray ecology, conservation and management. Frontiers in Marine Science, 6, 135. 10.3389/fmars.2019.00135

[cl21149-bib-0056] Yan, S. J. , Chughtai, A. A. , & Macintyre, C. R. (2017). Utility and potential of rapid epidemic intelligence from Internet‐based sources. International Journal of Infectious Diseases, 63, 77–87. 28765076 10.1016/j.ijid.2017.07.020

[cl21149-bib-0500] Yeung, W. J. J. , & Fok, Y. Y. (2014). United Nations Economic Commission for Europe. Springer Netherlands.

[cl21149-bib-0057] AbdelRahman, M. A. E. , Natarajan, A. , Hegde, R. , & Prakash, S. S. (2018). Assessment of land degradation using comprehensive geostatistical approach and remote sensing data in GIS‐model builder. Egyptian Journal of Remote Sensing and Space Sciences, 22(3), 323–334. 10.1016/j.ejrs.2018.03.002

[cl21149-bib-0058] Abt Associates . (2017). *Haryana Health GIS: Leveraging Technology to strengthen evidence based decision making in public health*. https://bit.ly/3lOiRg1

[cl21149-bib-0059] Acosta, M. M. , Pérez Miranda, R. , Romero Sánchez, M. E. , González Hernández, A. , & Martínez Ángel, L. (2017). Landsat ETM+ imaging for the estimation of the forest density in the southern region of the State of Mexico. Revista Mexicana de Ciencias Forestales, 8, 30–55.

[cl21149-bib-0060] Adhikary, P. P. , Barman, D. , Madhu, M. , Dash, C. J. , Jakhar, P. , Hombegowda, H. C. , Naik, B. S. , Sahoo, D. C. , & Beer, K. (2019). Land use and land cover dynamics with special emphasis on shifting cultivation in Eastern Ghats Highlands of India using remote sensing data and GIS. Environmental Monitoring and Assessment, 191, 1–15. 10.1007/s10661-019-7447-7 31037430

[cl21149-bib-0061] Agidew, A. A. , & Singh, K. N. (2017). The implications of land use and land cover changes for rural household food insecurity in the northeastern highlands of Ethiopia: The case of the Teleyayen sub‐watershed. Agriculture and Food Security, 6, 56.

[cl21149-bib-0062] Aguda, A. S. , Farinde, T. A. , Adegboyega, S. A. , & Olawole, M. O. (2013). Spatio‐temporal assessment of urban growth of medium‐size and nodal towns for sustainable management: Using GIS. Management of Environmental Quality: An International Journal, 24, 94–106. 10.1108/14777831311291159

[cl21149-bib-0063] Ahas, R. , Saluveer, E. , Tiru, M. , & Silm, S. (2008). Mobile positioning based tourism monitoring system: Positium barometer. In P. O'Connor , W. Höpken & U. Gretzel (Eds.), Presented at the Information and Communication Technologies in Tourism 2008 (pp. 475–485). Springer Vienna.

[cl21149-bib-0064] Ahmad, F. , & Goparaju, L. (2016). Geospatial technology in urban forest suitability: Analysis for Ranchi, Jharkhand, India. Ecological Questions, 24, 45–57.

[cl21149-bib-0065] Ahmad, F. , & Goparaju, L. (2017). Assessment of threats to forest ecosystems using geospatial technology in Jharkhand state of India. Current World Environment, 12, 355–365.

[cl21149-bib-0066] Aiello, A. , Adamo, M. , & Canora, F. (2015). Remote sensing and GIS to assess soil erosion with RUSLE3D and USPED at river basin scale in southern Italy. Catena, 131, 174–185.

[cl21149-bib-0067] Ajin, R. S. , Loghin, A. M. , Vinod, P. G. , Menon, A. R. R. , & Jacob, M. K. (2018). Forest fire risk assessment using geospatial techniques: A study in Mannarkkad forest division of Palakkad District, Kerala, India. Ecoterra, 15, 1–9.

[cl21149-bib-0068] Aklibasinda, M. (2019). Determining the active green areas and their adequacy by using satellite images and GIS: The case of Nevsehir city (Turkey). Fresenius Environmental Bulletin, 28, 7274–7281.

[cl21149-bib-0069] Aladangady, A. , Aron‐Dine, S. , Dunn, W. , Feiveson, L. , Lengermann, P. , & Sahm, C. (2019). From transactions data to economic statistics: Constructing real‐time, high‐frequency, geographic measures of consumer spending (No. w26253). National Bureau of Economic Research.

[cl21149-bib-0070] Al‐Bahrani, H. S. (2014). Spatial prediction and classification of water quality parameters for irrigation use in the Euphrates River (Iraq) using GIS and satellite image analyses. International Journal of Sustainable Development and Planning, 9, 389–399.

[cl21149-bib-0071] Albuquerque, M. , Espinoza, J. , Teixeira, P. , de Oliveira, A. , Corrêa, I. , & Calliari, L. (2013). Erosion or coastal variability: An evaluation of the DSAS and the change polygon methods for the determination of erosive processes on sandy beaches. Journal of Coastal Research, 165, 1710–1714.

[cl21149-bib-0072] Alegana, V. A. , Atkinson, P. M. , Pezzulo, C. , Sorichetta, A. , Weiss, D. , Bird, T. , Erbach‐Schoenberg, E. , & Tatem, A. J. (2015). Fine resolution mapping of population age‐structures for health and development applications. Journal of the Royal Society, Interface, 12, 20150073. 10.1098/rsif.2015.0073 25788540 PMC4387535

[cl21149-bib-0073] Allevato, E. , Saulino, L. , Cesarano, G. , Chirico, G. B. , D'Urso, G. , Falanga, B. , Salvatore, R. A. , Rossi, S. , Saracino, A. , & Bonanomi, G. (2019). Canopy damage by spring frost in European beech along the Apennines: Effect of latitude, altitude and aspect. Remote Sensing of Environment, 225, 431–440. 10.1016/j.rse.2019.03.023

[cl21149-bib-0074] Alshaikh, A. Y. (2015). Space applications for drought assessment in Wadi‐Dama (West Tabouk), KSA. Egyptian Journal of Remote Sensing and Space Sciences, 18, S43–S53. 10.1016/j.ejrs.2015.07.001

[cl21149-bib-0075] Althouse, B. M. , Ng, Y. Y. , & Cummings, D. A. T. (2011). Prediction of dengue incidence using search query surveillance. PLOS Neglected Tropical Diseases, 5, e1258. 21829744 10.1371/journal.pntd.0001258 PMC3149016

[cl21149-bib-0076] Alwadi, M. , & Chetty, G. (2015). Energy efficient data mining scheme for high dimensional data. Procedia Computer Science, 46, 483–490. 10.1016/j.procs.2015.02.047

[cl21149-bib-0077] Andres, L.A. , Bhatt, S. , Dasgupta, B. , & Echeniqu, J.A. (2018). *Geo‐spatial modeling of access to water and sanitation in Nigeria*. Policy Research Working Papers. The World Bank. 10.1596/1813-9450-8357

[cl21149-bib-0078] Antenucci, D. , Cafarella, M. , Levenstein, M. , Ré, C. , & Shapiro, M. D. (2014). *Using social media to measure labor market flows* (No. w20010). National Bureau of Economic Research. https://bit.ly/3pKXFKu

[cl21149-bib-0079] Armenteras, D. , Gibbes, C. , Anaya, J. A. , & Dávalos, L. M. (2017). Integrating remotely sensed fires for predicting deforestation for REDD+. Ecological Applications, 27, 1294–1304. 10.1002/eap.1522 28208227

[cl21149-bib-0080] Ashraf, S. , Afshari, H. , & Ebadi, A. G. (2011). Application of GIS for determination of groundwater quality suitable in crops influenced by irrigation water in the Damghan region of Iran. International Journal of Physical Sciences, 6, 843–854.

[cl21149-bib-0081] Askitas, N. , & Zimmermann, K. F. (2009). Google econometrics and unemployment forecasting. Applied Economics Quarterly, 55, 107–120.

[cl21149-bib-0082] Babaie‐Kafaky, S. , Mataji, A. , & Sani, N. A. (2009). Ecological capability assessment for multiple‐use in forest areas using GIS‐based multiple criteria decision making approach. American Journal of Environmental Sciences, 5, 714–721.

[cl21149-bib-0083] Badarinath, K. V. S. , Kiran Chand, T. R. , & Krishna, P. V. (2006). Agriculture crop residue burning in the Indo‐Gangetic Plains: A study using IRS‐P6 AWiFS satellite data. Current Science, 91, 1085.

[cl21149-bib-0084] Badarinath, K. V. S. , Kiran Chand, T. R. , & Krishna, P. V. (2009). Emissions from grassland burning in Kaziranga National Park, India: Analysis from IRS‐P6 AWiFS satellite remote sensing datasets. Geocarto International, 24, 89–97. 10.1080/10106040701207225

[cl21149-bib-0085] Bahuguna, A. , Shailesh, N. , & Dam, R. (2008). Impact of the tsunami and earthquake of 26 December 2004 on the vital coastal ecosystems of the Andaman and Nicobar Islands assessed using RESOURCESAT AWiFS data. International Journal of Applied Earth Observations and Geoinformation, 10, 229–237. 10.1016/j.jag.2008.02.010

[cl21149-bib-0086] Baltrusaitis, K. , Brownstein, J. S. , Scarpino, S. V. , Bakota, E. , Crawley, A. W. , Conidi, G. , Gunn, J. , Gray, J. , Zink, A. , & Santillana, M. (2018). Comparison of crowd‐sourced, electronic health records based, and traditional health‐care based influenza‐tracking systems at multiple spatial resolutions in the United States of America. BMC Infectious Diseases, 18. (15 August 2018). 30111305 10.1186/s12879-018-3322-3 PMC6094455

[cl21149-bib-0087] Banerjee, R. , & Srivastava, P. K. (2013). Reconstruction of contested landscape: Detecting land cover transformation hosting cultural heritage sites from Central India using remote sensing. Land Use Policy, 34, 193–203. 10.1016/j.landusepol.2013.03.005

[cl21149-bib-0088] Barbieri, T. , Despini, F. , & Teggi, S. (2018). A multi‐temporal analyses of land surface temperature using Landsat‐8 data and open source software: The case study of Modena, Italy. Sustainability, 10, 1678.

[cl21149-bib-0089] Barros, N. , Fontes, T. , Silva, M. P. , & Manso, M. C. (2013). How wide should be the adjacent area to an urban motorway to prevent potential health impacts from traffic emissions? Transportation Research Part A: Policy and Practice, 50, 113–128. 10.1016/j.tra.2013.01.021

[cl21149-bib-0090] Bartlett, J. , Krasodomski‐Jones, A. , Daniel, N. , Fisher, A. , & Jesperson, S. (2015). Social media for election communication and monitoring in Nigeria. Demos for the Department for International Development.

[cl21149-bib-0091] Baus, P. , Kováč, U. , Pauditšová, E. , Kohutková, I. , & Komorník, J. (2014). Identification of interconnections between landscape pattern and urban dynamics: Case study Bratislava, Slovakia. Ecological Indicators, 42, 104–111. 10.1016/j.ecolind.2013.12.011

[cl21149-bib-0092] Bekele, B. , Wu, W. , Legesse, A. , Temesgen, H. , & Yirsaw, E. (2018). Socio‐environmental impacts of land use/cover change in Ethiopian Central Rift Valley Lakes Region, East Africa. Applied Ecology and Environmental Research, 16, 6607–6632.

[cl21149-bib-0093] Bengtsson, L. , Gaudart, J. , Lu, X. , Moore, S. , Wetter, E. , Sallah, K. , Rebaudet, S. , & Piarroux, R. (2015). Using mobile phone data to predict the spatial spread of cholera. Scientific Reports, 5, 8923. 25747871 10.1038/srep08923 PMC4352843

[cl21149-bib-0094] Bengtsson, L. , Lu, X. , Thorson, A. , Garfield, R. , & von Schreeb, J. (2011). Improved response to disasters and outbreaks by tracking population movements with mobile phone network data: A post‐earthquake geospatial study in Haiti. PLOS Medicine, 8, e1001083. 21918643 10.1371/journal.pmed.1001083 PMC3168873

[cl21149-bib-0095] Berhan, G. , Tadesse, T. , Atnafu, S. , & Hill, S. (2011). Drought monitoring in food‐insecure areas of Ethiopia by using satellite technologies. In W. L. Filho (Ed.), Experiences of climate change adaptation in Africa (pp. 183–200). Springer.

[cl21149-bib-0096] Bharti, N. , Djibo, A. , Tatem, A. J. , Grenfell, B. T. , & Ferrari, M. J. (2016). Measuring populations to improve vaccination coverage. Scientific Reports, 6, 34541. 10.1038/srep34541 PMC5050518 27703191

[cl21149-bib-0097] Bharti, N. , Lu, X. , Bengtsson, L. , Wetter, E. , & Tatem, A. J. (2015). Remotely measuring populations during a crisis by overlaying two data sources. International Health, 7, 90–98. 10.1093/inthealth/ihv003 25733558 PMC4357797

[cl21149-bib-0098] Bick, I. A. , Bardhan, R. , & Beaubois, T. (2018). Applying fuzzy logic to open data for sustainable development decision‐making: A case study of the planned city Amaravati. Natural Hazards, 91, 1317–1339. 10.1007/s11069-018-3186-2

[cl21149-bib-0099] Blumenstock, J. E. (2018). Estimating economic characteristics with phone data. AEA Papers and Proceedings, 108, 72–76.

[cl21149-bib-0100] Blumenstock, J. (2014). Calling for better measurement: Estimating an individual's wealth and well‐being. ACM KDD (Data Mining for Social Good).

[cl21149-bib-0101] Blumenstock, J. , Cadamuro, G. , & On, R. (2015). Predicting poverty and wealth from mobile phone metadata. Science, 350, 1073–1076. 10.1126/science.aac4420 26612950

[cl21149-bib-0102] Blumenstock, J. E. , Chokkalingam, R. , Gaikwad, V. , & Kondepudi, S. (2014, December). Probabilistic inference of unknown locations: Exploiting collective behavior when individual data is scarce. *Proceedings of the fifth ACM Symposium on Computing for Development* (pp. 103–112).

[cl21149-bib-0103] Blumenstock, J. E. (2012). Inferring patterns of internal migration from mobile phone call records: Evidence from Rwanda. Information Technology for Development, 18, 107–125. 10.1080/02681102.2011.643209

[cl21149-bib-0104] Blumenstock, J. , & Eagle, N. (2010, December). Mobile divides: Gender, socioeconomic status, and mobile phone use in Rwanda. *Proceedings of the 4th ACM/IEEE International Conference on Information and Communication Technologies and Development* (pp. 1–10).

[cl21149-bib-0105] Blumenstock, J. E. , & Keleher, N. (2015, December). The price is right? statistical evaluation of a crowd‐sourced market information system in liberia. *Proceedings of the 2015 Annual Symposium on Computing for Development* (pp. 117–125).

[cl21149-bib-0106] Blumenstock, J. , Maldeniya, D. , & Lokanathan, S. (2017, November). Understanding the impact of urban infrastructure: New insights from population‐scale data. *Proceedings of the Ninth International Conference on Information and Communication Technologies and Development* (pp. 1–12).

[cl21149-bib-0107] Blumenstock, J. , Shen, Y. , & Eagle, N. (2010). A method for estimating the relationship between phone use and wealth. *QualMeetsQuant workshop at the 4th international conference on information and Communication Technologies and Development* (Vol. 13, pp. 114–125).

[cl21149-bib-0108] Bogomolov, A. , Lepri, B. , Staiano, J. , Letouzé, E. , Oliver, N. , Pianesi, F. , & Pentland, A. (2015). Moves on the street: Classifying crime hotspots using aggregated anonymized data on people dynamics. Big Data, 3, 148–158. 10.1089/big.2014.0054 27442957

[cl21149-bib-0109] Bosco, C. , Alegana, V. , Bird, T. , Pezzulo, C. , Bengtsson, L. , Sorichetta, A. , Steele, J. , Hornby, G. , Ruktanonchai, C. , Ruktanonchai, N. , Wetter, E. , & Tatem, A. J. (2017). Exploring the high‐resolution mapping of gender‐disaggregated development indicators. Journal of the Royal Society Interface, 14(129), 20160825. 28381641 10.1098/rsif.2016.0825 PMC5414904

[cl21149-bib-0110] Bosco, C. , Tejedor‐Garavito, N. , de Rigo, D. , Pezzulo, C. , Bengtsson, L. , Tatem, A. J. , & Bird, T. J. (2017, October). *Mapping the interaction between development aid and stunting in Nigeria*. 2017 International Population Conference. IUSSP.

[cl21149-bib-0111] Bosco, C. , Tejedor‐Garavito, N. , de Rigo, D. , Tatem, A. J. , Pezzulo, C. , Wood, R. , & Bird, T. (2018). Geostatistical tools to map the interaction between development aid and indices of need. Washington, DC: AidData.

[cl21149-bib-0112] Botha, R. , Labuschagne, C. , Williams, A. G. , Bosman, G. , Brunke, E. G. , Rossouw, A. , & Lindsay, R. (2018). Characterising fifteen years of continuous atmospheric radon activity observations at Cape Point (South Africa). Atmospheric Environment, 176, 30–39. 10.1016/j.atmosenv.2017.12.010

[cl21149-bib-0113] Brdar, S. , Gavrić, K. , Ćulibrk, D. , & Crnojević, V. (2016). Unveiling spatial epidemiology of HIV with mobile phone data. Scientific Reports, 6, 19342. 10.1038/srep19342 26758042 PMC4725841

[cl21149-bib-0114] Brewin, R. J. W. , de Mora, L. , Jackson, T. , Brewin, T. G. , & Shutler, J. (2015). On the potential of surfers to monitor environmental indicators in the coastal zone. PLOS One, 10, e0127706. 26154173 10.1371/journal.pone.0127706 PMC4496071

[cl21149-bib-0115] Burke, M. , & Lobell, D. B. (2017). Satellite‐based assessment of yield variation and its determinants in smallholder African systems. Proceedings of the National Academy of Sciences of the United States of America, 114, 2189–2194. 10.1073/pnas.1616919114 28202728 PMC5338538

[cl21149-bib-0116] Bundervoet, T. , Maiyo, L. , & Sanghi, A. (2015). *Bright lights, big cities: Measuring national and subnational economic growth in Africa from outer space, with an application to Kenya and Rwanda*. Policy Research Working Papers. The World Bank. 10.1596/1813-9450-7461

[cl21149-bib-0117] Cabral, A. I. R. , Saito, C. , Pereira, H. , & Laques, A. E. (2018). Deforestation pattern dynamics in protected areas of the Brazilian Legal Amazon using remote sensing data. Applied Geography, 100, 101–115. 10.1016/j.apgeog.2018.10.003

[cl21149-bib-0118] Cahoon, D.R. , Olker, J.H. , Yeates, A.G. , Guntenspergen, G.R. , Grace, J.B. , Adamowicz, S.C. , Anisfeld, S.C. , Baldwin, A.H. , Barrett, N. , Beckett, L. , Benzecry, A. , Blum, L.K. , Burdick, D.M. , Crouch, W. , Ekberg, M.C. , Fernald, S. , Grimes, K.W. , Grzyb, J. , Hartig, E.K. , … Mitchell, L.R. (2019). *Hurricane Sandy impacts on coastal wetland resilience*. Open‐File Report, US Geological Survey.

[cl21149-bib-0119] Cai, Y. , Guan, K. , Lobell, D. , Potgieter, A. B. , Wang, S. , Peng, J. , Xu, T. , Asseng, S. , Zhang, Y. , You, L. , & Peng, B. (2019). Integrating satellite and climate data to predict wheat yield in Australia using machine learning approaches. Agricultural and Forest Meteorology, 274, 144–159. 10.1016/j.agrformet.2019.03.010

[cl21149-bib-0120] Candelieri, A. , Soldi, D. , & Archetti, F. (2015). Short‐term forecasting of hourly water consumption by using automatic metering readers data. Procedia Engineering, 119, 844–853. 10.1016/j.proeng.2015.08.948

[cl21149-bib-0121] Carreiras, J. M. B. , Jones, J. , Lucas, R. M. , & Gabriel, C. (2014). Land use and land cover change dynamics across the Brazilian Amazon: insights from extensive time‐series analysis of remote sensing data. PLOS One, 9, e104144. 25099362 10.1371/journal.pone.0104144 PMC4123946

[cl21149-bib-0122] Caughlin, T. T. , Ruktanonchai, N. , Acevedo, M. A. , Lopiano, K. K. , Prosper, O. , Eagle, N. , & Tatem, A. J. (2013). Place‐based attributes predict community membership in a mobile phone communication network. PLOS One, 8, e56057. 23451034 10.1371/journal.pone.0056057 PMC3579832

[cl21149-bib-0123] Cavallo, A. (2013). Online and official price indexes: Measuring Argentina's inflation. Journal of Monetary Economics, 60, 152–165. 10.1016/j.jmoneco.2012.10

[cl21149-bib-0124] Chae, S. W. , Kwon, S. J. , & Lee, D. H. (2018). Predicting infectious disease using deep learning and big data. International Journal of Environmental Research and Public Health, 15, 1596. 30060525 10.3390/ijerph15081596 PMC6121625

[cl21149-bib-0125] Chakraborty, A. , Seshasai, M. V. R. , Reddy, C. S. , & Dadhwal, V. K. (2018). Persistent negative changes in seasonal greenness over different forest types of India using MODIS time series NDVI data (2001–14). Ecological Indicators, 85, 887–903. 10.1016/j.ecolind.2017.11.032

[cl21149-bib-0126] Chantarat, S. , Rakwatin, P. , & Charumilind, C. (2017). Farmers and pixels: Toward sustainable agricultural finance with space technology (No. 75). Puey Ungphakorn Institute for Economic Research.

[cl21149-bib-0127] Cheema, M. J. M. , Bakhsh, A. , Mahmood, T. , & Liaqat, M. U. (2016). *Assessment of water allocations using remote sensing and GIS modeling for Indus Basin* (No. 036). Pakistan. Working paper.

[cl21149-bib-0128] Chen, X. , & Nordhaus, W. D. (2011). Using luminosity data as a proxy for economic statistics. Proceedings of the National Academy of Sciences of the United States of America, 108, 8589–8594. 10.1073/pnas.1017031108 21576474 PMC3102367

[cl21149-bib-0129] Chen, Y. , Jin, G. Z. , Kumar, N. , & Shi, G. (2013). The promise of Beijing: Evaluating the impact of the 2008 Olympic Games on air quality. Journal of Environmental Economics and Management, 66, 424–443. 10.1016/j.jeem.2013.06.005

[cl21149-bib-0130] Chen, X. , Song, H. , Li, Y. S. , Yuan, Z. , Wai, O. W. H. , Li, Z. , Xu, Z. , & Zhang, B. (2006). Spatial analysis of water quality using a combination of GIS and statistical software. International Journal of Environment and Pollution, 28, 274–296. 10.1504/IJEP.2006.011212

[cl21149-bib-0131] Choi, H. , & Varian, H. (2012). Predicting the Present with Google Trends. Economic Record, 88, 2–9.

[cl21149-bib-0132] Chong, W. K. , Naganathan, H. , Liu, H. , Ariaratnam, S. , & Kim, J. (2018). Understanding infrastructure resiliency in Chennai, India Using Twitter's geotags and texts: A preliminary study. Engineering, 4, 218–223. 10.1016/j.eng.2018.03.010

[cl21149-bib-0133] Civelli, A. , Horowitz, A. , & Teixeira, A. (2017). *Foreign aid and growth at the subnational level* (No. 36). Aiddata working paper.

[cl21149-bib-0134] Clifford, E. , Mulligan, S. , Comer, J. , & Hannon, L. (2018). Flow‐signature analysis of water consumption in nonresidential building water networks using high‐resolution and medium‐resolution smart meter data: Two case studies. Water Resources Research, 54, 88–106.

[cl21149-bib-0135] Convergne, E. , & Snyder, M. R. (2015). Making maps to make peace: Geospatial technology as a tool for UN Peacekeeping. International Peacekeeping, 22, 565–586. 10.1080/13533312.2015.1094193

[cl21149-bib-0136] Coskun, H.G. , Alganci, U. , Usta, G. , & Celik, H. (2009). Analysis of the provincial structure of Sariyer/Istanbul using Remote Sensing and GIS. Remote sensing for a changing Europe. *Proceedings of the 28th Symposium of the European Association of Remote Sensing Laboratories*, Istanbul, Turkey, June 2–5, 2008.

[cl21149-bib-0137] Dadhich, P. N. , & Hanaoka, S. (2011). Spatio‐temporal urban growth modeling of Jaipur, India. Journal of Urban Technology, 18, 45–65. 10.1080/10630732.2011.615567

[cl21149-bib-0138] Dalezios, N. R. , Mplanta, A. , & Domenikiotis, C. (2011). Remotely sensed cotton evapotranspiration for irrigation water management in vulnerable agriculture of central Greece. Journal of Information Technology in Agriculture, 4, 1.

[cl21149-bib-0139] Damanik‐Ambarita, M. N. , Boets, P. , Nguyen, T. , Hanh, T. , Forio, M. A. E. , Everaert, G. , Lock, K. , Musonge, P. L. S. , Suhareva, N. , Bennetsen, E. , Gobeyn, S. , Ho, T. L. , Dominguez‐Granda, L. , & Goethals, P. L. M. (2018). Impact assessment of local land use on ecological water quality of the Guayas river basin (Ecuador). Ecological Informatics, 48, 226–237. 10.1016/j.ecoinf.2018.08.009

[cl21149-bib-0140] Davies, K. P. , Murphy, R. J. , & Bruce, E. (2016). Detecting historical changes to vegetation in a Cambodian protected area using the Landsat TM and ETM+ sensors. Remote Sensing of Environment, 187, 332–344. 10.1016/j.rse.2016.10.027

[cl21149-bib-0141] De Jong, B. , Anaya, C. , Masera, O. , Olguín, M. , Paz, F. , Etchevers, J. , Martínez, R. D. , Guerrero, G. , & Balbontín, C. (2010). Greenhouse gas emissions between 1993 and 2002 from land‐use change and forestry in Mexico. Forest Ecology and Management, 260, 1689–1701. 10.1016/j.foreco.2010.08.011

[cl21149-bib-0142] Decuyper, A. , Rutherford, A. , Wadhwa, A. , Bauer, J.‐M. , Krings, G. , Gutierrez, T. , Blondel, V. D. , & Luengo‐Oroz, M. A. (2014). Estimating food consumption and poverty indices with mobile phone data. arXiv.

[cl21149-bib-0143] Dehkordi, L. F. , Sohrabi, T. A. , Ghanavizbaf, M. H. , & Ghazavi, R. (2016). Drought monitoring by using of MODIS satellite images in dry lands (case study: Isfahan rangelands). Geography and Environmental Planning, 27, 177–190.

[cl21149-bib-0144] Deng, X. Z. , Gibson, J. , & Wang, P. (2017). Relationship between landscape diversity and crop production: A case study in the Hebei Province of China based on multi‐source data integration. Journal of Cleaner Production, 142, 985–992.

[cl21149-bib-0145] Dengiz, O. , & Baskan, O. (2009). Land quality assessment and sustainable land use in Salt Lake (Tuz Gölü) specially protected area. Environmental Monitoring and Assessment, 148, 233–243. 18214696 10.1007/s10661-008-0154-4

[cl21149-bib-0146] Dennison, P. E. , Thorpe, A. K. , Pardyjak, E. R. , Roberts, D. A. , Qi, Y. , Green, R. O. , Bradley, E. S. , & Funk, C. C. (2013). High spatial resolution mapping of elevated atmospheric carbon dioxide using airborne imaging spectroscopy: Radiative transfer modeling and power plant plume detection. Remote Sensing of Environment, 139, 116–129. 10.1016/j.rse.2013.08.001

[cl21149-bib-0147] Development Alternatives Inc . (2008). *Liberia environmental threats an opportunities assessment (LETOA): GIS and spatial data infrastructure final assessment*. https://bit.ly/3nGy57r

[cl21149-bib-0148] Deville, P. , Linard, C. , Martin, S. , Gilbert, M. , Stevens, F. R. , Gaughan, A. E. , Blondel, V. D. , & Tatem, A. J. (2014). Dynamic population mapping using mobile phone data. Proceedings of the National Academy of Sciences of the United States of America, 111(45), 15888–15893. 10.1073/pnas.1408439111 25349388 PMC4234567

[cl21149-bib-0149] Dewan, A. M. , Yamaguchi, Y. , & Rahman, M. Z. (2012). Dynamics of land use/cover changes and the analysis of landscape fragmentation in Dhaka Metropolitan, Bangladesh. GeoJournal, 77, 315–330.

[cl21149-bib-0150] Dimobe, K. , Kouakou, J. L. N. , Tondoh, J. E. , Zoungrana, B. J. B. , Forkuor, G. , & Ouédraogo, K. (2018). Predicting the potential impact of climate change on carbon stock in semi‐arid West African Savannas. Land, 7, 1.

[cl21149-bib-0151] Dingel, J. I. , Miscio, A. , & Davis, D. R. (2019). Cities, lights, and skills in developing economies. Journal of Urban Economics, 103174. 10.1016/j.jue.2019.05.005

[cl21149-bib-0152] Dobra, A. , Williams, N. E. , & Eagle, N. (2015). Spatiotemporal detection of unusual human population behavior using mobile phone data. PLOS One, 10(3), e0120449. 10.1371/journal.pone.0120449 25806954 PMC4373934

[cl21149-bib-0153] Doll, C. N. H. , Muller, J.‐P. , & Morley, J. G. (2006). Mapping regional economic activity from night‐time light satellite imagery. Ecological Economics, 57, 75–92. 10.1016/j.ecolecon.2005.03.007

[cl21149-bib-0154] Doll, C. N. H. , & Pachauri, S. (2010). Estimating rural populations without access to electricity in developing countries through night‐time light satellite imagery. Energy Policy, 38, 5661–5670. 10.1016/j.enpol.2010.05.014

[cl21149-bib-0155] Dushaj, L. , Sallaku, F. , Tafaj, S. , & Rrapo, S. (2011). Application on GIS for land use planning in central part of Albania, Maminas commune. Albanian Journal of Agricultural Sciences, 10, 23–29.

[cl21149-bib-0156] Eagle, N. , Macy, M. , & Claxton, R. (2010). Network diversity and economic development. Science, 328, 1029–1031. 10.1126/science.1186605 20489022

[cl21149-bib-0157] Ebener, S. , Murray, C. , Tandon, A. , & Elvidge, C. (2005). From wealth to health: Modelling the distribution of income per capita at the sub‐national level using night‐time light imagery. International Journal of Health Geographics, 4, 5. 10.1186/1476-072X-4-5 15705196 PMC549533

[cl21149-bib-0158] Engstrom, R. , Hersh, J. , & Newhouse, D. (2017). *Poverty from space: Using high‐resolution satellite imagery for estimating economic well‐being*. Policy Research Working Papers. The World Bank. 10.1596/1813-9450-8284

[cl21149-bib-0159] El‐Zeiny, A. , & El‐Kafrawy, S. (2017). Assessment of water pollution induced by human activities in Burullus Lake using Landsat 8 operational land imager and GIS. Egyptian Journal of Remote Sensing and Space Sciences, 20, S49–S56. 10.1016/j.ejrs.2016.10.002

[cl21149-bib-0160] Emadodin, I. , Taravat, A. , & Rajaei, M. (2016). Effects of urban sprawl on local climate: A case study, north central Iran. Urban Climate, 17, 230–247. 10.1016/j.uclim.2016.08.008

[cl21149-bib-0161] Enoguanbhor, E. C. , Gollnow, F. , Nielsen, J. O. , Lakes, T. , & Walker, B. B. (2019). Land cover change in the Abuja City‐Region, Nigeria: Integrating GIS and remotely sensed data to support land use planning. Sustainability, 11, 1.

[cl21149-bib-0162] Erhan, L. , Ndubuaku, M. , Ferrara, E. , Richardson, M. , Sheffield, D. , Ferguson, F. J. , Brindley, P. , & Liotta, A. (2019). Analyzing objective and subjective data in social sciences: Implications for smart cities. IEEE Access, 7, 19890–19906. 10.1109/access.2019.2897217

[cl21149-bib-0163] Faber, B. , & Gaubert, C. (2019). Tourism and economic development: Evidence from Mexico's Coastline. American Economic Review, 109, 2245–2293. 10.1257/aer.20161434

[cl21149-bib-0164] Feyisa, G. L. , Meilby, H. , Fensholt, R. , & Proud, S. R. (2014). Automated Water Extraction Index: A new technique for surface water mapping using Landsat imagery. Remote Sensing of Environment, 140, 23–35. 10.1016/j.rse.2013.08.029

[cl21149-bib-0165] Feyera, S. (2018). Community perception of land use/land cover change and its impacts on biodiversity and ecosystem services in northwestern Ethiopia. Journal of Sustainable Development in Africa, 20, 108–126.

[cl21149-bib-0166] Finger, F. , Genolet, T. , Lorenzo, M. , de Magny, G. C. , Manga, N. M. , Rinaldo, A. , & Bertuzzo, E. (2016). Mobile phone data highlights the role of mass gatherings in the spreading of cholera outbreaks. Proceedings of the National Academy of Sciences of the United States of America, 113, 6421–6426. 10.1073/pnas.1522305113 27217564 PMC4988598

[cl21149-bib-0167] Firchow, P. , & MacGinty, R. (2017). Including hard‐to‐access populations using mobile phone surveys and participatory indicators. Sociological Methods and Research, 49, 133–160. 10.1177/0049124117729702

[cl21149-bib-0168] Foster, A. , Gutierrez, E. , & Kumar, N. (2009). Voluntary compliance, pollution levels, and infant mortality in Mexico. American Economic Review, 99, 191–197. 10.1257/aer.99.2.191 29505212

[cl21149-bib-0169] Frías‐Martínez, V. , Soguero Ruiz, C. , & Frias‐Martinez, E. (2012). Estimation of urban commuting patterns using cellphone network data. *Proceedings of the ACM SIGKDD International Conference on Knowledge Discovery and Data Mining*. 10.1145/2346496.2346499

[cl21149-bib-0170] Frías‐Martínez, V. , Soto, V. , Hohwald, H. , & Frias‐Martinez, E. (2012). *Characterizing urban landscapes using geolocated tweets*. The 2012 International Conference on Privacy, Security, Risk and Trust and 2012 International Conference on Social Computing (pp. 239–248). 10.1109/SocialCom-PASSAT.2012.19

[cl21149-bib-0171] Frías‐Martínez, V. , Virseda‐Jerez, J. , & Frias‐Martinez, E. (2012). On the relation between socio‐economic status and physical mobility. Information Technology for Development, 18, 91–106. 10.1080/02681102.2011.630312

[cl21149-bib-0172] Garcia, A. J. , Pindolia, D. K. , Lopiano, K. K. , & Tatem, A. J. (2014). Modeling internal migration flows in sub‐Saharan Africa using census microdata. Migration Studies, 3, 89–110. 10.1093/migration/mnu036

[cl21149-bib-0173] Georgiadis, G. , Spanou, S. , Kokkoris, I. , Tiniakou, A. , & Georgiadis, T. (2014). Introducing an integrated monitoring system for natural ecosystems: The example of Strofilia wetlands in Western Peloponnese, Greece. International Journal of Environmental Research, 8, 1195–1202.

[cl21149-bib-0174] Ghimire, S. , Deo, R. C. , Raj, N. , & Mi, J. (2019). Deep learning neural networks trained with MODIS satellite‐derived predictors for long‐term global solar radiation prediction. Energies, 12, 1.

[cl21149-bib-0175] Gillespie, T. W. , Chu, J. , Frankenberg, E. , & Thomas, D. (2007). Assessment and prediction of natural hazards from satellite imagery. Progress in Physical Geography: Earth and Environment, 31, 459–470. 10.1177/0309133307083296 PMC4144012 25170186

[cl21149-bib-0176] Goab, J. , & Uczkowski, B. (2017). GIS maps and analysis in designing forest road system on mountainous areas. Electronic Journal of Polish Agricultural Universities, 20(4), 14.

[cl21149-bib-0177] Gonder, J. , Markel, T. , & Thornton, M. (2007). Using global positioning system travel data to assess real‐world energy use of plug‐in hybrid electric vehicles. Transportation Research Record: Journal of the Transportation Research Board, 2017, 26–32. 10.3141/2017-04

[cl21149-bib-0178] Gong, H. L. , Tang, T. , Gong, Z. N. , Li, X. J. , Chen, Y. Z. , & Zhao, W. J. (2015). Spatial and temporal trend of water resources in Beijing, China during 1999–2012 and its impact analysis. British Journal of Environment and Climate Change, 5, 176–188.

[cl21149-bib-0179] González, M. C. , Hidalgo, C. A. , & Barabási, A.‐L. (2008). Understanding individual human mobility patterns. Nature, 453, 779–782. 10.1038/nature06958 18528393

[cl21149-bib-0180] Grant, A. T. J. , McKinney, N. L. , & Ries, R. (2018). An approach to quantifying rainwater harvesting potential using imagery, geographic information systems (GIS) and LiDAR data. Water Science and Technology: Water Supply, 18, 108–118.

[cl21149-bib-0181] Guan, K. , Wu, J. , Kimball, J. S. , Anderson, M. C. , Frolking, S. , Li, B. , Hain, C. R. , & Lobell, D. B. (2017). The shared and unique values of optical, fluorescence, thermal and microwave satellite data for estimating large‐scale crop yields. Remote Sensing of Environment, 199, 33–349. 10.1016/j.rse.2017.06.043

[cl21149-bib-0182] Gumma, M. K. , Birhanu, B. Z. , Mohammed, I. A. , Tabo, R. , & Whitbread, A. M. (2016). Prioritization of watersheds across Mali using remote sensing data and GIS techniques for agricultural development planning. Water, 8(6), 260.

[cl21149-bib-0183] Guo, Y. , & Su, X. M. (2012). Mobile device‐based reporting system for Sichuan earthquake‐affected areas infectious disease reporting in China. Biomedical and Environmental Sciences, 25, 724–729. 23228844 10.3967/0895-3988.2012.06.016

[cl21149-bib-0184] Gutierrez, T. , Krings, G. , & Blondel, V. D. (2013). Evaluating socio‐economic state of a country analyzing airtime credit and mobile phone datasets. arXiv, 1309, 4496.

[cl21149-bib-0185] Han, W. Y. , Liu, G. H. , Su, X. K. , Wu, X. , & Chen, L. (2019). Assessment of potential land degradation and recommendations for management in the south subtropical region, Southwest China. Land Degradation and Development, 30, 979–990.

[cl21149-bib-0186] Hartter, J. , & Southworth, J. (2009). Dwindling resources and fragmentation of landscapes around parks: Wetlands and forest patches around Kibale National Park, Uganda. Landscape Ecology, 24, 643–656. 10.1007/s10980-009-9339-7

[cl21149-bib-0187] Hassan, M. M. , & Nazem, M. N. I. (2016). Examination of land use/land cover changes, urban growth dynamics, and environmental sustainability in Chittagong city, Bangladesh. Environment, Development and Sustainability, 18, 697–716. 10.1007/s10668-015-9672-8

[cl21149-bib-0188] Hayano, R. S. , & Adachi, R. (2013). Estimation of the total population moving into and out of the 20 km evacuation zone during the Fukushima NPP accident as calculated using “Auto‐GPS” mobile phone data. Proceedings of the Japan Academy, Series B, 89(5), 196–199. 10.2183/pjab.89.196 PMC3722575 23666090

[cl21149-bib-0189] Head, A. , Manguin, M. , Tran, N. , & Blumenstock, J. E. (2017). Can human development be measured with satellite imagery? Proceedings of the Ninth ACM/IEEE International Conference on Information and Communication Technologies and Development, ICTD '17, https://bit.ly/3kLaMHL

[cl21149-bib-0190] Heathman, G. C. , Larose, M. , & Ascough, J. C., II (2009). Soil and water assessment tool evaluation of soil and land use geographic information system data sets on simulated stream flow. Journal of Soil and Water Conservation (Ankeny), 64, 17–32.

[cl21149-bib-0191] Heft‐Neal, S. , Burney, J. , Bendavid, E. , Voss, K. , & Burke, M. (2019). *Air pollution and infant mortality: Evidence from Saharan Dust*. National Bureau of Economic Research Working Paper Series No. 26107. 10.3386/w26107

[cl21149-bib-0192] Heinzelman, J. , Brown, R. , & Meier, P. (2011). Mobile technology, crowdsourcing and peace mapping: New theory and applications for conflict management. In M. Poblet (Ed.), Mobile technologies for conflict management: Online dispute resolution, governance, participation (pp. 39–53). Springer Netherlands. 10.1007/978-94-007-1384-0_4

[cl21149-bib-0193] Hernandez, M. , Hong, L. , Frias‐Martinez, V. , & Frias‐Martinez, E. (2017). *Estimating poverty using cell phone data: Evidence from Guatemala*. Policy Research Working Papers. The World Bank. 10.1596/1813-9450-7969

[cl21149-bib-0194] Hively, W. D. , Lang, M. , McCarty, G. W. , Keppler, J. , Sadeghi, A. , & McConnell, L. L. (2009). Using satellite remote sensing to estimate winter cover crop nutrient uptake efficiency. Journal of Soil and Water Conservation (Ankeny), 64, 303–313.

[cl21149-bib-0195] Honarvar, A. R. , & Sami, A. (2019). Towards sustainable smart city by particulate matter prediction using urban big data, excluding expensive air pollution infrastructures. Big Data Research, 17, 56–65. 10.1016/j.bdr.2018.05.006

[cl21149-bib-0196] Hristova, D. , Rutherford, A. , Anson, J. , Luengo‐Oroz, M. , & Mascolo, C. (2016). The international postal network and other global flows as proxies for national wellbeing. PLOS One, 11, e0155976. 27248142 10.1371/journal.pone.0155976 PMC4889156

[cl21149-bib-0197] Huan, J. , Cao, W. J. , & Liu, X. Q. (2017). A dissolved oxygen prediction method based on K‐means clustering and the ELM neural network: A case study of the Changdang Lake, China. Applied Engineering in Agriculture, 33, 461–469.

[cl21149-bib-0198] Huang, H. P. , Li, Q. Z. , & Zhang, Y. (2019). Urban residential land suitability analysis combining remote sensing and social sensing data: A case study in Beijing, China. Sustainability, 11, 10.3390/su11082255

[cl21149-bib-0199] Huang, Z. R. , Ling, X. M. , Wang, P. , Zhang, F. , Mao, Y. P. , Lin, T. , & Wang, F. Y. (2018). Modeling real‐time human mobility based on mobile phone and transportation data fusion. Transportation Research Part C‐Emerging Technologies, 96, 251–269. 10.1016/j.trc.2018.09.016

[cl21149-bib-0200] Hung, T. L. , Tuyen, V. D. , & Hiep, D. N. (2015). Evaluation of soil erosion risk using remote sensing and GIS data (a case study: Lang Chanh district, Thanh Hoa province, Vietnam). Vestnik OrelGAU, 57–64.

[cl21149-bib-0201] Ibrahim, M. , & Koch, B. (2015). Assessment and mapping of groundwater vulnerability using SAR concentrations and GIS: A case study in Al‐Mafraq, Jordan. Journal of Water Resource and Protection, 7, 588–596.

[cl21149-bib-0202] Ikemi, H. (2017). Geologically constrained changes to landforms caused by human activities in the 20th century: A case study from Fukuoka Prefecture, Japan. Applied Geography, 87, 115–126.

[cl21149-bib-0203] Jahan, S. , Kalita, S. , & Kumar, B. B. (2015). An assessment of land use–land cover change using geoinformatics in Sonai‐Rupai Wildlife Sanctuary, Assam, India. Journal of Environmental Research and Development, 9, 1257–1263.

[cl21149-bib-0204] Jahani, E. , Sundsøy, P. , Bjelland, J. , Bengtsson, L. , Pentland, A., ('S.') , & de Montjoye, Y.‐A. (2017). Improving official statistics in emerging markets using machine learning and mobile phone data. EPJ Data Science, 6, 3. 10.1140/epjds/s13688-017-0099-3

[cl21149-bib-0205] Jain, M. , Singh, B. , Srivastava, A. A. K. , Malik, R. K. , McDonald, A. J. , & Lobell, D. B. (2017). Using satellite data to identify the causes of and potential solutions for yield gaps in India's Wheat Belt. Environmental Research Letters, 12, 094011. 10.1088/1748-9326/aa8228

[cl21149-bib-0206] Jain, R. K. , Smith, K. M. , Culligan, P. J. , & Taylor, J. E. (2014). Forecasting energy consumption of multi‐family residential buildings using support vector regression: Investigating the impact of temporal and spatial monitoring granularity on performance accuracy. Applied Energy, 123, 168–178.

[cl21149-bib-0207] Jamal, S. , Javed, A. , & Khanday, Y. (2016). Evaluation of land degradation and socio‐environmental issues: A case study of semi arid watershed in Western Rajasthan. Journal of Environmental Protection, 7, 1132–1147.

[cl21149-bib-0208] Jäppinen, S. , Toivonen, T. , & Salonen, M. (2013). Modelling the potential effect of shared bicycles on public transport travel times in Greater Helsinki: An open data approach. Applied Geography, 43, 13–24. 10.1016/j.apgeog.2013.05.010

[cl21149-bib-0209] Järv, O. , Ahas, R. , Saluveer, E. , Derudder, B. , & Witlox, F. (2012). Mobile phones in a traffic flow: A geographical perspective to evening rush hour traffic analysis using call detail records. PLOS One, 7, e49171. 23155461 10.1371/journal.pone.0049171 PMC3498329

[cl21149-bib-0210] Jawdar, M. Y. A. , Shiobara, M. , & Onuma, T. (2005). Chapter 2 Monitoring of coastal environment using satellite images in the United Arab Emirates. Developments in Earth and Environmental Sciences, 3, 13–29. 10.1016/S1571-9197(05)80026-6

[cl21149-bib-0211] Jayasekara, M. J. P. T. M. , Kadupitiya, H. K. , & Vitharana, U. W. A. (2018). Mapping of soil erosion hazard zones of Sri Lanka. Tropical Agricultural Research, 29, 135–146.

[cl21149-bib-0212] Jean, N. , Burke, M. , Xie, M. , Davis, W. M. , Lobell, D. B. , & Ermon, S. (2016). Combining satellite imagery and machine learning to predict poverty. Science, 353, 790–794. 10.1126/science.aaf7894 27540167

[cl21149-bib-0213] Jiang, Y. , Shi, T. , & Gu, X. (2016). Healthy urban streams: The ecological continuity study of the Suzhou creek corridor in Shanghai. Cities, 59, 80–94. 10.1016/j.cities.2016.06.002

[cl21149-bib-0214] Jin, Z. , Azzari, G. , Burke, M. , Aston, S. , & Lobell, D. B. (2017). Mapping smallholder yield heterogeneity at multiple scales in Eastern Africa. Remote Sensing, 9(9), 931. 10.3390/rs9090931

[cl21149-bib-0215] Jones, M. O. , Allred, B. W. , Naugle, D. E. , Maestas, J. D. , Donnelly, P. , Metz, L. J. , Karl, J. , Smith, R. , Bestelmeyer, B. , Boyd, C. , Kerby, J. D. , & McIver, J. D. (2018). Innovation in rangeland monitoring: Annual, 30 m, plant functional type percent cover maps for US rangelands, 1984–2017. Ecosphere, 9, e02430.

[cl21149-bib-0216] Jones, M. O. , Kimball, J. S. , & Jones, L. A. (2013). Satellite microwave detection of boreal forest recovery from the extreme 2004 wildfires in Alaska and Canada. Global Change Biology, 19, 3111–3122. 23749682 10.1111/gcb.12288

[cl21149-bib-0217] Juniper, S. K. , Thornborough, K. , Douglas, K. , & Hillier, J. (2019). Remote monitoring of a deep‐sea marine protected area: The Endeavour Hydrothermal Vents. Aquatic Conservation‐Marine and Freshwater Ecosystems, 29, 84–102. 10.1002/aqc.3020

[cl21149-bib-0218] Kabisch, N. , Selsam, P. , Kirsten, T. , Lausch, A. , & Bumberger, J. (2019). A multi‐sensor and multi‐temporal remote sensing approach to detect land cover change dynamics in heterogeneous urban landscapes. Ecological Indicators, 99, 273–282.

[cl21149-bib-0219] Kaiser, M. F. , Aboulela, H. , El Serehy, H. , & Ezz Edin, H. (2010). Spectral enhancement of SPOT imagery data to assess marine pollution near Port Said, Egypt. International Journal of Remote Sensing, 31, 1753–1764. 10.1080/01431160902926624

[cl21149-bib-0220] Kaliraj, S. , Chandrasekar, N. , & Ramachandran, K. K. (2017). Mapping of coastal landforms and volumetric change analysis in the south west coast of Kanyakumari, South India using remote sensing and GIS techniques. Egyptian Journal of Remote Sensing and Space Sciences, 20, 265–282. 10.1016/j.ejrs.2016.12.006

[cl21149-bib-0221] Kalvani, S. R. , Sharaai, A. , Manaf, L. , & Hamidian, A. (2019). Assessing ground and surface water scarcity indices using ground and surface water footprints in the Tehran province of Iran. Applied Ecology and Environmental Research, 17, 4985–4997.

[cl21149-bib-0222] Kan, Z. H. , Tang, L. L. , Kwan, M. P. , Ren, C. , Liu, D. , Pei, T. , Liu, Y. , Deng, M. , & Li, Q. Q. (2018a). Fine‐grained analysis on fuel‐consumption and emission from vehicles trace. Journal of Cleaner Production, 203, 340–352. 10.1016/j.jclepro.2018.08.222

[cl21149-bib-0223] Kan, Z. H. , Tang, L. L. , Kwan, M. P. , & Zhang, X. (2018b). Estimating vehicle fuel consumption and emissions using GPS big data. International Journal of Environmental Research and Public Health, 15. 10.3390/ijerph15040566 29561813 PMC5923608

[cl21149-bib-0224] Karan, S. K. , & Samadder, S. R. (2016). Accuracy of land use change detection using support vector machine and maximum likelihood techniques for open‐cast coal mining areas. Environmental Monitoring and Assessment, 188, 486. 27461425 10.1007/s10661-016-5494-x

[cl21149-bib-0225] Karapetyan, D. , & d'Adda, G. (2014). Determinants of conservation among the rural poor: A charitable contribution experiment. Ecological Economics, 99, 74–87. 10.1016/j.ecolecon.2014.01.009

[cl21149-bib-0226] Keeratikasikorn, C. , & Bonafoni, S. (2018). Urban heat island analysis over the land use zoning plan of Bangkok by means of Landsat 8 imagery. Remote Sensing, 10, 440.

[cl21149-bib-0227] Khakh, A. K. , & Fast, V. (2017). Measuring spatial accessibility of healthcare services in calgary (poster). Journal of Transport and Health, 7, S13–S14. 10.1016/j.jth.2017.11.023

[cl21149-bib-0228] Khalil, A. , Hanich, L. , Hakkou, R. , & Lepage, M. (2014). GIS‐based environmental database for assessing the mine pollution: A case study of an abandoned mine site in Morocco. Journal of Geochemical Exploration, 144, 468–477. 10.1016/j.gexplo.2014.03.023

[cl21149-bib-0229] Khomba, D. C. , & Trew, A. (2017). *Aid and growth in Malawi*. AidData Working Paper #42. AidData, Williamsburg, VA.

[cl21149-bib-0230] Khorchani, M. , Vicente‐Serrano, S. M. , Azorin‐Molina, C. , Garcia, M. , Martin‐Hernandez, N. , Peña‐Gallardo, M. , El Kenawy, A. , & Domínguez‐Castro, F. (2018). Trends in LST over the peninsular Spain as derived from the AVHRR imagery data. Global and Planetary Change, 166, 75–93. 10.1016/j.gloplacha.2018.04.006

[cl21149-bib-0231] Kim, H. Y. , & Lee, H. K. (2014). Enhanced validity and reliability of spatial decision support systems (SDSS) for sustainable transportation decision‐making. Applied Geography, 51, 65–71. 10.1016/j.apgeog.2014.03.009

[cl21149-bib-0232] Kim, J. K. , Noh, J. W. , Son, K. H. , & Kim, I. J. (2012). Impacts of GIS data quality on determination of runoff and suspended sediments in the Imha watershed in Korea. Geosciences Journal, 16, 181–192.

[cl21149-bib-0233] Kim, S. J. , Marsch, L. A. , Hancock, J. T. , & Das, A. K. (2017). Scaling up research on drug abuse and addiction through social media big data. Journal of Medical Internet Research, 19. 10.2196/jmir.6426 PMC5686417 29089287

[cl21149-bib-0234] Kim, Y. H. , Im, J. , Ha, H. K. , Choi, J. K. , & Ha, S. H. (2014). Machine learning approaches to coastal water quality monitoring using GOCI satellite data. GIScience and Remote Sensing, 51, 158–174.

[cl21149-bib-0235] Kinyanjui, M. J. (2011). NDVI‐based vegetation monitoring in Mau forest complex, Kenya. African Journal of Ecology, 49, 165–174.

[cl21149-bib-0236] Klemens, B. , Coppola, A. , & Shron, M. (2015). *Estimating local poverty measures using satellite images: A pilot application to Central America*. Policy Research Working Papers. The World Bank. 10.1596/1813-9450-7329

[cl21149-bib-0237] Knadel, M. , Thomsen, A. , Schelde, K. , & Greve, M. H. (2015). Soil organic carbon and particle sizes mapping using vis‐NIR, EC and temperature mobile sensor platform. Computers and Electronics in Agriculture, 114, 134–144.

[cl21149-bib-0238] Knudby, A. , Roelfsema, C. , Lyons, M. , Phinn, S. , & Jupiter, S. (2011). Mapping fish community variables by integrating field and satellite data, object‐based image analysis and modeling in a traditional Fijian fisheries management area. Remote Sensing, 3, 460–483.

[cl21149-bib-0239] Kong, C. F. , Lan, H. , Yang, G. , & Xu, K. (2016). Geo‐environmental suitability assessment for agricultural land in the rural‐urban fringe using BPNN and GIS: A case study of Hangzhou. Environmental Earth Sciences, 75, 1136. 10.1007/s12665-016-5956-z

[cl21149-bib-0240] Kong, C. , Xu, K. , & Wu, C. (2006). Classification and extraction of urban land‐use information from high‐resolution image based on object multi‐features. Journal of China University of Geosciences, 17, 151–157. 10.1016/S1002-0705(06)60021-6

[cl21149-bib-0241] Kraemer, M. U. G. , Faria, N. R. , Reiner, R. C. J. , Golding, N. , Nikolay, B. , Stasse, S. , Johansson, M. A. , Salje, H. , Faye, O. , Wint, G. R. W. , Niedrig, M. , Shearer, F. M. , Hill, S. C. , Thompson, R. N. , Bisanzio, D. , Taveira, N. , Nax, H. H. , Pradelski, B. S. R. , Nsoesie, E. O. , … Cauchemez, S. (2017). Spread of yellow fever virus outbreak in Angola and the Democratic Republic of the Congo 2015–16: A modelling study. The Lancet Infectious Diseases, 17, 330–338. 10.1016/S1473-3099(16)30513-8 28017559 PMC5332542

[cl21149-bib-0242] Kreindler, G. , & Miyauchi, Y. (2015). Commuting and productivity: Quantifying urban economic activity using cell phone data. https://bit.ly/3nGHNqn

[cl21149-bib-0243] Krishnadas, M. , Agarwala, M. , Sridhara, S. , & Eastwood, E. (2018). Parks protect forest cover in a tropical biodiversity hotspot, but high human population densities can limit success. Biological Conservation, 223, 147–155. 10.1016/j.biocon.2018.04.034

[cl21149-bib-0244] Kropáček, J. , Braun, A. , Kang, S. , Feng, C. , Ye, Q. , & Hochschild, V. (2012). Analysis of lake level changes in Nam Co in central Tibet utilizing synergistic satellite altimetry and optical imagery. International Journal of Applied Earth Observations and Geoinformation, 17, 3–11. 10.1016/j.jag.2011.10.001

[cl21149-bib-0245] Krtička, L. , Tomčíková, I. , & Rakytová, I. (2018). Development versus conservation: Evaluation of landscape structure changes in Demänovská Valley, Slovakia. Journal of Mountain Science, 15, 1153–1170.

[cl21149-bib-0246] Kubitza, C. , Krishna, V. V. , Urban, K. , Alamsyah, Z. , & Qaim, M. (2018). Land property rights, agricultural intensification, and deforestation in Indonesia. Ecological Economics, 147, 312–321.

[cl21149-bib-0247] Kwasi Appeaning, A. (2015). Monitoring sea level rise‐induced hazards along the coast of Accra in Ghana. Natural Hazards, 1293, 10.1007/s11069-015-1771-1

[cl21149-bib-0248] Ladd, B. , Laffan, S. W. , Amelung, W. , Peri, P. L. , Silva, L. C. R. , Gervassi, P. , Bonser, S. P. , Navall, M. , & Sheil, D. (2013). Estimates of soil carbon concentration in tropical and temperate forest and woodland from available GIS data on three continents. Global Ecology and Biogeography, 22, 461–469.

[cl21149-bib-0249] Lai, S. , zu Erbach‐Schoenberg, E. , Pezzulo, C. , Ruktanonchai, N. W. , Sorichetta, A. , Steele, J. , Li, T. , Dooley, C. A. , & Tatem, A. J. (2019). Exploring the use of mobile phone data for national migration statistics. Palgrave Communications, 5, 34. 10.1057/s41599-019-0242-9 31579302 PMC6774788

[cl21149-bib-0250] Le Blanc, D. , & Perez, R. (2008). The relationship between rainfall and human density and its implications for future water stress in sub‐Saharan Africa. Ecological Economics, 66, 319–336.

[cl21149-bib-0251] Le Menach, A. , Tatem, A. J. , Cohen, J. M. , Hay, S. I. , Randell, H. , Patil, A. P. , & Smith, D. L. (2011). Travel risk, malaria importation and malaria transmission in Zanzibar. Scientific Reports, 1, 93. 10.1038/srep00093 22355611 PMC3216579

[cl21149-bib-0252] Leauthaud, C. , Belaud, G. , Duvail, S. , Moussa, R. , Grünberger, O. , & Albergel, J. (2013). Characterizing floods in the poorly gauged wetlands of the Tana River Delta, Kenya, using a water balance model and satellite data. Hydrology and Earth System Sciences, 17, 3059–3075.

[cl21149-bib-0253] Lee, E. C. , Asher, J. M. , Goldlust, S. , Kraemer, J. D. , Lawson, A. B. , & Bansal, S. (2016). Mind the scales: Harnessing spatial big data for infectious disease surveillance and inference. Journal of Infectious Diseases, 214, S409–S413. 28830109 10.1093/infdis/jiw344 PMC5144899

[cl21149-bib-0254] Lee, S. M. , Lee, S. R. , Lee, M. J. , & Jung, H. S. (2018). Spatial assessment of urban flood susceptibility using data mining and geographic information system (GIS) tools. Sustainability, 10, 648.

[cl21149-bib-0255] Lee, T. , Park, H. , & Lee, J. (2019). Collaborative accountability for sustainable public health: A Korean perspective on the effective use of ICT‐based health risk communication. Government Information Quarterly, 36, 226–236. 10.1016/j.giq.2018.12.008 32288166 PMC7125608

[cl21149-bib-0256] Lehtomäki, J. , Tomppo, E. , Kuokkanen, P. , Hanski, I. , & Moilanen, A. (2009). Applying spatial conservation prioritization software and high‐resolution GIS data to a national‐scale study in forest conservation. Forest Ecology and Management, 258, 2439–2449. 10.1016/j.foreco.2009.08.026

[cl21149-bib-0257] Li, H. M. , Ma, Y. X. , Aide, T. M. , & Liu, W. J. (2008). Past, present and future land‐use in Xishuangbanna, China and the implications for carbon dynamics. Forest Ecology and Management, 255, 16–24.

[cl21149-bib-0258] Li, S. , Kyllo, J. M. , & Guo, X. (2013). An integrated model based on a hierarchical indices system for monitoring and evaluating urban sustainability. Sustainability, 5(2), 524–559.

[cl21149-bib-0259] Li, T. , Dejby, J. , Albert, M. , Bengtsson, L. , & Lefebvre, V. (2019). Detecting individual internal displacements following a sudden‐onset disaster using time series analysis of call detail records. arXiv, 1908, 02377.

[cl21149-bib-0260] Li, T. , Dejby, J. , Albert, M. , Bengtsson, L. , & Lefebvre, V. (2019). Estimating the resilience to natural disasters by using call detail records to analyse the mobility of internally displaced persons. arXiv, 1908, 02381.

[cl21149-bib-0261] Li, X. (2014). Can night‐time light images play a role in evaluating the Syrian Crisis? International Journal of Remote Sensing, 35(18), 6648–6661. 10.1080/01431161.2014.971469

[cl21149-bib-0262] Linard, C. , Kabaria, C. W. , Gilbert, M. , Tatem, A. J. , Gaughan, A. E. , Stevens, F. R. , Sorichetta, A. , Noor, A. M. , & Snow, R. W. (2017). Modelling changing population distributions: An example of the Kenyan Coast, 1979–2009. International Journal of Digital Earth, 10, 1017–1029. 10.1080/17538947.2016.1275829 29098016 PMC5632926

[cl21149-bib-0263] Llorente, A. , Garcia‐Herranz, M. , Cebrian, M. , & Moro, E. (2015). Social media fingerprints of unemployment. PLOS One, 10, e0128692. 10.1371/journal.pone.0128692 26020628 PMC4447438

[cl21149-bib-0264] Lobell, D. B. , Azzari, G. , Burke, M. , & Gourlay, S. (2018). *Eyes in the sky, boots on the ground: Assessing satellite‐ and ground‐based approaches to crop yield measurement and analysis in Uganda*. Policy Research Working Papers. The World Bank. 10.1596/1813-9450-8374

[cl21149-bib-0265] Lobell, D. B. , Ortiz‐Monasterio, J. I. , & Lee, A. S. (2010). Satellite evidence for yield growth opportunities in Northwest India. Field Crops Research, 118, 13–20. 10.1016/j.fcr.2010.03.013

[cl21149-bib-0266] Lopreite, M. , Puliga, M. , & Riccaboni, M. (2018). *The global health networks: A comparative analysis of tuberculosis, malaria and pneumonia using social media data*. https://bit.ly/3nHEDCK

[cl21149-bib-0267] Louail, T. , Lenormand, M. , Cantu, R. , Ros, O. G. , Picornell, M. , Herranz, R. , Frias‐Martinez, E. , Ramasco, J. J. , & Barthelemy, M. (2014). From mobile phone data to the spatial structure of cities. Scientific Reports, 4, 5276. 10.1038/srep05276 24923248 PMC4055889

[cl21149-bib-0268] Lu, D. , Hetrick, S. , Moran, E. , & Li, G. (2012). Application of time series Landsat images to examining land‐use/land‐cover dynamic change. Photogrammetric Engineering and Remote Sensing, 78(7), 747–755. 25328256 10.14358/pers.78.7.747 PMC4201056

[cl21149-bib-0269] Lu, X. , Bengtsson, L. , & Holme, P. (2012). Predictability of population displacement after the 2010 Haiti earthquake. Proceedings of the National Academy of Sciences of the United States of America, 109(29), 11576–11581. 10.1073/pnas.1203882109 22711804 PMC3406871

[cl21149-bib-0270] Lu, X. , & Brelsford, C. (2014). Network structure and community evolution on twitter: Human behavior change in response to the 2011 Japanese earthquake and tsunami. Scientific Reports, 4, 6773. 10.1038/srep06773 25346468 PMC4209381

[cl21149-bib-0271] Lu, X. , Wetter, E. , Bharti, N. , Tatem, A. J. , & Bengtsson, L. (2013). Approaching the limit of predictability in human mobility. Scientific Reports, 3, 2923. 10.1038/srep02923 24113276 PMC3795357

[cl21149-bib-0272] Lu, X. , Wrathall, D. J. , Sundsøy, P. R. , Nadiruzzaman, M. , Wetter, E. , Iqbal, A. , Qureshi, T. , Tatem, A. , Canright, G. , Engø‐Monsen, K. , & Bengtsson, L. (2016a). Unveiling hidden migration and mobility patterns in climate stressed regions: A longitudinal study of six million anonymous mobile phone users in Bangladesh. Global Environmental Change, 38, 1–7. 10.1016/j.gloenvcha.2016.02.002

[cl21149-bib-0273] Lu, X. , Wrathall, D. J. , Sundsøy, P. , Nadiruzzaman, M. , Wetter, E. , Iqbal, A. , Qureshi, T. , Tatem, A. , Canright, G. , Engø‐Monsen, K. , & Bengtsson, L. (2016b). Detecting climate adaptation with mobile network data in Bangladesh: Anomalies in communication, mobility and consumption patterns during cyclone Mahasen. Climatic Change, 138, 505–519. 10.1007/s10584-016-1753-7 32355373 PMC7175666

[cl21149-bib-0274] Lupia, F. , Baiocchi, V. , Lelo, K. , & Pulighe, G. (2017). Exploring rooftop rainwater harvesting potential for food production in urban areas. Agriculture (London), 7, 1.

[cl21149-bib-0275] Madden, M. , & Ross, A. (2009). Genocide and GIScience: Integrating personal narratives and geographic information science to study human rights. Professional Geographer, 61, 508–526. 10.1080/00330120903163480

[cl21149-bib-0276] Madhawa, K. , Lokanathan, S. , Maldeniya, D. , & Samarajiva, R. (2015, August). *Using mobile network big data for land use classification*. Communication Policy Research South Conference.

[cl21149-bib-0277] Madu, I. A. (2011). Spatial vulnerability of rural Nigeria to climate change: Implications for internal security. International Journal of Climate Change: Impacts and Responses, 3, 79–98.

[cl21149-bib-0278] Mahler, H. , Searle, S. , Plotkin, M. , Kulindwa, Y. , Greenberg, S. , Mlanga, E. , Njeuhmeli, E. , & Lija, G. (2015). Covering the last kilometer: Using gis to scale‐up voluntary medical male circumcision services in Iringa and Njombe regions, Tanzania. Global Health: Science and Practice, 3(3), 503–515. 26374807 10.9745/GHSP-D-15-00151 PMC4570020

[cl21149-bib-0279] Maina, J. , Venus, V. , McClanahan, T. R. , & Ateweberhan, M. (2008). Modelling susceptibility of coral reefs to environmental stress using remote sensing data and GIS models. Ecological Modelling, 212, 180–199. 10.1016/j.ecolmodel.2007.10.033

[cl21149-bib-0280] Malleson, N. , & Andresen, M. A. (2015). The impact of using social media data in crime rate calculations: Shifting hot spots and changing spatial patterns. Cartography and Geographic Information Science, 42, 112–121. 10.1080/15230406.2014.905756

[cl21149-bib-0281] Mao, H. , Shuai, X. , Ahn, Y. Y. , & Bollen, J. (2015). Quantifying socio‐economic indicators in developing countries from mobile phone communication data: Applications to Côte d'Ivoire. EPJ Data Science, 4, 15. 10.1140/epjds/s13688-015-0053-1

[cl21149-bib-0282] March, R. G. , & Smith, E. H. (2012). Modeling potential coastal vegetation response to sea level rise and storm surge on estuarine peninsulas. Journal of Coastal Research, 28, 993–1007.

[cl21149-bib-0283] Marfai Muh, A. (2014). Impact of sea level rise to coastal ecology: A case study on the Northern Part Of Java Island, Indonesia. Quaestiones Geographicae, 33, 107–114.

[cl21149-bib-0284] Martins, F. R. , Pereira, E. B. , & Abreu, S. L. (2007). Satellite‐derived solar resource maps for Brazil under SWERA project. Solar Energy, 81, 517–528. 10.1016/j.solener.2006.07.009

[cl21149-bib-0285] Mathew, A. , Khandelwal, S. , & Nivedita, K. (2018). Spatio‐temporal variations of surface temperatures of Ahmedabad city and its relationship with vegetation and urbanization parameters as indicators of surface temperatures. Remote Sensing Applications: Society and Environment, 11, 119–139. 10.1016/j.rsase.2018.05.003

[cl21149-bib-0286] Melchiorri, M. , Pesaresi, M. , Florczyk, A. J. , Corbane, C. , & Kemper, T. (2019). Principles and applications of the global human settlement layer as baseline for the land use efficiency indicator‐DSDG 11.3.1. ISPRS International Journal of Geo‐Information, 8(2), 96. 10.3390/ijgi8020096

[cl21149-bib-0287] Merem, C. E. , Isokpehi, P. , Wesley, J. , Nwagboso, E. , Richardson, C. , Fageir, S. , Iwehabura, S. , & Crisler, M. (2014). The analysis of coal mining impacts on West Virginia's environment. British Journal of Applied Science and Technology, 4, 1171–1197.

[cl21149-bib-0288] Miles, V. V. , & Esau, I. (2016). Spatial heterogeneity of greening and browning between and within bioclimatic zones in northern West Siberia. Environmental Research Letters, 11, 115002.

[cl21149-bib-0289] Min, B. , Gaba, K. M. , Sarr, O. F. , & Agalassou, A. (2013). Detection of rural electrification in Africa using DMSP‐OLS night lights imagery. International Journal of Remote Sensing, 34, 8118–8141. 10.1080/01431161.2013.833358

[cl21149-bib-0290] Moffitt, D. L. , & Lalit, K. (2018). Remote sensing of a shallow, fringing reef platform for analysis of island sector susceptibility and development of a coastal vulnerability index. Journal of Coastal Research, 34, 122–135.

[cl21149-bib-0291] Mokgedi, L. , Nobert, J. , & Munishi, S. (2019). Assessment of lake surface dynamics using satellite imagery and in‐situ data: Case of Lake Ngami in North‐West Botswana. Physics and Chemistry of the Earth, 112, 175–186. 10.1016/j.pce.2018.12.008

[cl21149-bib-0292] Molla, M. B. (2015). Land use/land cover dynamics in the Central Rift Valley region of Ethiopia: Case of Arsi Negele District. African Journal of Agricultural Research, 10, 434–449.

[cl21149-bib-0293] Mondal, P. , & Tatem, A. J. (2012). Uncertainties in measuring populations potentially impacted by sea level rise and coastal flooding. PLOS One, 7, e48191. 23110208 10.1371/journal.pone.0048191 PMC3480473

[cl21149-bib-0294] Mori, H. , Sah, A. K. , Sah, B. P. , Yamaya, M. , & Senthil, S. (2015). An approach for monitoring the reforestation and conservation efforts by local communities. International Journal of Environmental and Rural Development, 6, 91–96.

[cl21149-bib-0295] Moss, R. , Naghizade, E. , Tomko, M. , & Geard, N. (2019). What can urban mobility data reveal about the spatial distribution of infection in a single city? BMC Public Health, 19, 656. 31142311 10.1186/s12889-019-6968-x PMC6542035

[cl21149-bib-0296] Moufaddal, W. (2005). Use of satellite imagery as environmental impact assessment tool: A case Study from the New Egyptian Red Sea Coastal Zone. Environmental Monitoring and Assessment, 107, 427–452. 10.1007/s10661-005-3576-2 16418927

[cl21149-bib-0297] Msoffe, F. U. , Said, M. Y. , Ogutu, J. O. , Kifugo, S. C. , de Leeuw, J. , van Gardingen, P. , & Reid, R. S. (2011). Spatial correlates of land‐use changes in the Maasai‐Steppe of Tanzania: Implications for conservation and environmental planning. International Journal of Biodiversity and Conservation, 3, 280–290.

[cl21149-bib-0298] Musaoglu, N. , Gurel, M. , Ulugtekin, N. , Tanik, A. , & Seker, D. Z. (2006). Use of remotely sensed data for analysis of land‐use change in a highly urbanized district of Mega City, Istanbul. Journal of Environmental Science and Health, Part A: Toxic/Hazardous Substances and Environmental Engineering, 41, 2057–2069. 10.1080/10934520600780719 16849146

[cl21149-bib-0299] Musaoglu, N. , Tanik, A. , Dikerler, T. , & Buhur, S. (2015). Use of remote sensing and geographic information systems in the determination of high‐risk areas regarding marine traffic in the Istanbul Strait. Environmental Hazards: Human and Policy Dimensions, 14, 54–73. 10.1080/17477891.2014.986042

[cl21149-bib-0300] Nackoney, J. , Rybock, D. , Dupain, J. , & Facheux, C. (2013). Coupling participatory mapping and GIS to inform village‐level agricultural zoning in the Democratic Republic of the Congo. Landscape and Urban Planning, 110, 164–174.

[cl21149-bib-0301] Nawapramote, W. , Prabudhanitisarn, S. , Sangawongse, S. , & Promburom, P. (2014). Co‐management in land demarcation to reduce forest utilization conflicts: A case study of Mae Tia‐Mae Tae watershed, Chom Thong district, Chiang Mai province. Environment and Natural Resources Journal, 12, 47–57.

[cl21149-bib-0302] Nieves, J. J. , Stevens, F. R. , Gaughan, A. E. , Linard, C. , Sorichetta, A. , Hornby, G. , & Tatem, A. J. (2017). Examining the correlates and drivers of human population distributions across low‐and middle‐income countries. Journal of the Royal Society Interface, 14(137), 20170401. 29237823 10.1098/rsif.2017.0401 PMC5746564

[cl21149-bib-0303] Niranjana, K. V. , & Sathish, A. (2011). Remote sensing and GIS approach for watershed based resources management in the Eastern Dry Zone of Karnataka. Mysore Journal of Agricultural Sciences, 45, 316–321.

[cl21149-bib-0304] Noori, A. M. , Biswajeet, P. , & Ajaj, Q. M. (2019). Dam site suitability assessment at the Greater Zab River in northern Iraq using remote sensing data and GIS. Journal of Hydrology (Amsterdam), 574, 964–979.

[cl21149-bib-0305] Novak, J. , Ahas, R. , Aasa, A. , & Silm, S. (2013). Application of mobile phone location data in mapping of commuting patterns and functional regionalization: A pilot study of Estonia. Journal of Maps, 9, 10–15. 10.1080/17445647.2012.762331

[cl21149-bib-0306] Ojoyi, M. M. , Antwi‐Agyei, P. , Mutanga, O. , Odindi, J. , & Abdel‐Rahman, E. M. (2015). An analysis of ecosystem vulnerability and management interventions in the Morogoro region landscapes, Tanzania. Tropical Conservation Science, 8, 662–680.

[cl21149-bib-0307] Oloo, F. (2018). Mapping rural road networks from global positioning system (GPS) trajectories of motorcycle Taxis in Sigomre Area, Siaya County, Kenya. ISPRS International Journal of Geo‐Information, 7(8), 309. 10.3390/ijgi7080309

[cl21149-bib-0308] Ozbay, G. , Fan, C. , & Yang, Z. (2017). Relationship between land use and water quality and its assessment using hyperspectral remote sensing in mid‐atlantic estuaries. Water Quality, 169–222.

[cl21149-bib-0309] Papastergiadou, E. , Kagalou, I. , Stefanidis, K. , Retalis, A. , & Leonardos, I. (2010). Effects of anthropogenic influences on the trophic state, land uses and aquatic vegetation in a shallow Mediterranean Lake: Implications for restoration. Water Resources Management, 24, 415–435. 10.1007/s11269-009-9453-y

[cl21149-bib-0310] Pape, U. , Parisotto, L. , Lefebvre, V. , Qader, S. , Ninneman, A. , & Bird, T. (2019a). Estimating poverty in a fragile context: The high frequency survey in South Sudan. The World Bank.

[cl21149-bib-0311] Pape, U. , Wollburg, P. , Lefebvre, V. , Qader, S. , Ninneman, A. , Thomson, D. , & Bird, T. (2019b). Estimation of poverty in Somalia using innovative methodologies. The World Bank.

[cl21149-bib-0312] Pappalardo, L. , Pedreschi, D. , Smoreda, Z. , & Giannotti, F. (2015). *Using big data to study the link between human mobility and socio‐economic development*. The 2015 IEEE International Conference on Big Data (Big Data), pp. 871–78. 10.1109/BigData.2015.7363835

[cl21149-bib-0313] Pastor‐Escuredo, D. , Morales‐Guzmán, A. , Torres‐Fernández, Y. , Bauer, J. , Wadhwa, A. , Castro‐Correa, C. , Romanoff, L. , Lee, J.G. , Rutherford, A. , Frías‐Martínez, V. , Oliver, N. , Frias‐Martinez, E. , & Luengo‐Oroz, M. (2014). *Flooding through the lens of mobile phone activity*. IEEE Global Humanitarian Technology Conference (GHTC 2014), pp. 279–86. 10.1109/GHTC.2014.6970293

[cl21149-bib-0314] Pásztor, L. , Laborczi, A. , Takács, K. , Szatmári, G. , Fodor, N. , Illés, G. , Farkas‐Iványi, K. , Bakacsi, Z. , & Szabó, J. (2017). Chapter 9—Compilation of functional soil maps for the support of spatial planning and land management in Hungary. In P. Pereira , E. C. Brevik , M. Muñoz‐Rojas & B. A. Miller (Eds.), Soil mapping and process modeling for sustainable land use management (pp. 293–317). Elsevier. 10.1016/B978-0-12-805200-6.00009-8

[cl21149-bib-0315] Patel, N. N. , Angiuli, E. , Gamba, P. , Gaughan, A. , Lisini, G. , Stevens, F. R. , Tatem, A. J. , & Trianni, G. (2015). Multitemporal settlement and population mapping from Landsat using Google Earth Engine. International Journal of Applied Earth Observation and Geoinformation, 35, 199–208. 10.1016/j.jag.2014.09.005

[cl21149-bib-0316] Patel, N. N. , Stevens, F. R. , Huang, Z. , Gaughan, A. E. , Elyazar, I. , & Tatem, A. J. (2017). Improving large area population mapping using geotweet densities. Transactions in GIS, 21, 317–331. 10.1111/tgis.12214 28515661 PMC5412862

[cl21149-bib-0317] Patode, R. S. , Pande, C. B. , Nagdeve, M. B. , Moharir, K. N. , & Wankhade, R. M. (2017). Planning of conservation measures for watershed management and development by using geospatial technology: A case study of Patur watershed in Akola district of Maharashtra. Current World Environment, 12, 708–716.

[cl21149-bib-0318] Peak, C. M. , Wesolowski, A. , zu Erbach‐Schoenberg, E. , Tatem, A. J. , Wetter, E. , Lu, X. , Power, D. , Weidman‐Grunewald, E. , Ramos, S. , Moritz, S. , Buckee, C. O. , & Bengtsson, L. (2018). Population mobility reductions associated with travel restrictions during the Ebola epidemic in Sierra Leone: Use of mobile phone data. International Journal of Epidemiology, 47, 1562–1570. 10.1093/ije/dyy095 29947788 PMC6208277

[cl21149-bib-0319] Pellikka, P. K. E. , Clark, B. J. F. , Gosa, A. G. , Himberg, N. , Hurskainen, P. , Maeda, E. , Mwang'ombe, J. , Omoro, L. M. A. , & Siljander, M. (2013). Chapter 13—Agricultural expansion and its consequences in the Taita Hills, Kenya. Developments in Earth Surface Processes, 16, 165–179. 10.1016/B978-0-444-59559-1.00013-X

[cl21149-bib-0320] Peng, J. , Wu, J. S. , Yin, H. , Li, Z. G. , Chang, Q. , & Mu, T. L. (2008). Rural land use change during 1986–2002 in Lijiang, China, based on remote sensing and GIS data. Sensors, 8, 8201–8223. 10.3390/s8128201 27873983 PMC3791014

[cl21149-bib-0321] Pfeifer, M. , Boyle, M. J. W. , Dunning, S. , & Olivier, P. I. (2019). Forest floor temperature and greenness link significantly to canopy attributes in South Africa's fragmented coastal forests. PeerJ, 7, e6190. 30648017 10.7717/peerj.6190 PMC6330204

[cl21149-bib-0322] Picoli, M. C. A. , Camara, G. , Sanches, I. , Simões, R. , Carvalho, A. , Maciel, A. , Coutinho, A. , Esquerdo, J. , Antunes, J. , Begotti, R. A. , Arvor, D. , & Almeida, C. (2018). Big earth observation time series analysis for monitoring Brazilian agriculture. ISPRS Journal of Photogrammetry and Remote Sensing, 145, 328–339. 10.1016/j.isprsjprs.2018.08.007

[cl21149-bib-0323] Pillarisetti, A. , Allen, T. , Ruiz‐Mercado, I. , Edwards, R. , Chowdhury, Z. , Garland, C. , Hill, L. D. , Johnson, M. , Litton, C. D. , Lam, N. L. , Pennise, D. , & Smith, K. R. (2017). Small, smart, fast, and cheap: Microchip‐based sensors to estimate air pollution exposures in rural households. Sensors (Basel, Switzerland), 17, 1879. 10.3390/s17081879 28812989 PMC5579926

[cl21149-bib-0324] Pindolia, D. K. , Garcia, A. J. , Huang, Z. , Fik, T. , Smith, D. L. , & Tatem, A. J. (2014). Quantifying cross‐border movements and migrations for guiding the strategic planning of malaria control and elimination. Malaria Journal, 13, 169. 10.1186/1475-2875-13-169 24886389 PMC4057586

[cl21149-bib-0325] Ping, L. , Jin, W. , Sangaiah, A. K. , Yang, X. , & Xinchun, Y. (2019). Analysis and prediction of water quality using LSTM deep neural networks in IoT environment. Sustainability, 11(7), 2058.

[cl21149-bib-0326] Pokhriyal, N. , & Jacques, D. C. (2017). Combining disparate data sources for improved poverty prediction and mapping. Proceedings of the National Academy of Sciences of the United States of America, 114, E9783–E9792. 10.1073/pnas.1700319114 29087949 PMC5699027

[cl21149-bib-0327] Potter, C. (2014a). Monitoring the production of Central California coastal rangelands using satellite remote sensing. Journal of Coastal Conservation, 18, 213–220. 10.1007/s11852-014-0308-1

[cl21149-bib-0328] Potter, C. (2014b). Ten years of forest cover change in the Sierra Nevada detected using Landsat satellite image analysis. International Journal of Remote Sensing, 35, 7136–7153. 10.1080/01431161.2014.968687

[cl21149-bib-0329] Rahman, M. M. , Csaplovics, E. , & Koch, B. (2008). Satellite estimation of forest carbon using regression models. International Journal of Remote Sensing, 29, 6917–6936.

[cl21149-bib-0330] Rai, S. M. , Upreti, B. N. , Dhakal, S. , Bhattarai, T. N. , Adhikari, B. R. , Bajracharya, S. R. , & Yoshida, M. (2017). Climate change impact on glacier retreat and local community in the Langtang Valley, Central Nepal. Journal of Development Innovations, 1(1), 45–59.

[cl21149-bib-0331] Rangel, M. A. , & Vogl, T. (2016). *Agricultural fires and infant health*. National Bureau of Economic Research Working Paper Series No. 22955. 10.3386/w22955

[cl21149-bib-0332] Raucoules, D. , Le Cozannet, G. , Wöppelmann, G. , de Michele, M. , Gravelle, M. , & Daag Arturo, M. M. (2013). High nonlinear urban ground motion in Manila (Philippines) from 1993 to 2010 observed by DInSAR: Implications for sea‐level measurement. Remote Sensing of Environment, 139, 386–397. 10.1016/j.rse.2013.08.021

[cl21149-bib-0333] Reddy, C. S. , Alekhya, V. V. L. P. , Saranya, K. R. L. , Athira, K. , Jha, C. S. , Diwakar, P. G. , & Dadhwal, V. K. (2017). Monitoring of fire incidences in vegetation types and Protected Areas of India: Implications on carbon emissions. Journal of Earth System Science, 126, 11.

[cl21149-bib-0334] Redowan, M. , Akter, S. , & Islam, N. (2014). Analysis of forest cover change at Khadimnagar National Park, Sylhet, Bangladesh, using Landsat TM and GIS data. Journal of Forestry Research, 25, 393–400.

[cl21149-bib-0335] Robin, M. , Chapuis, J. L. , & Lebouvier, M. (2011). Remote sensing of vegetation cover change in islands of the Kerguelen archipelago. Polar Biology, 34, 1689–1700.

[cl21149-bib-0336] Robinson, T. , Emwanu, T. , & Rogers, D. (2007). Environmental approaches to poverty mapping: An example from Uganda. Information Development, 23, 205–215. 10.1177/0266666907079077

[cl21149-bib-0337] Rodriguez, F. M. , Garcia, A. C. , Alonso, I. G. , & Casanova, E. Z. (2016). Using the big data generated by the Smart Home to improve energy efficiency management. Energy Efficiency, 9, 249–260. 10.1007/s12053-015-9361-3

[cl21149-bib-0338] Rogers, D. , Emwanu, T. , & Robinson, T. (2006). Poverty mapping in Uganda: An analysis using remotely sensed and other environmental data. Pro‐Poor Livestock Policy Initiative, Rome, Italy, 67.

[cl21149-bib-0339] Roy, K. C. , Cebrian, M. , & Hasan, S. (2019). Quantifying human mobility resilience to extreme events using geo‐located social media data. Epj Data Science, 8. 10.1140/epjds/s13688-019-0196-6

[cl21149-bib-0340] Rui, Y. H. , Fu, D. F. , Ha, D. M. , Radhakrishnan, M. , Zevenbergen, C. , & Pathirana, A. (2018). Urban surface water quality, flood water quality and human health impacts in Chinese cities. What do we know? Water, 10, 240.

[cl21149-bib-0341] Ruktanonchai, N. W. , DeLeenheer, P. , Tatem, A. J. , Alegana, V. A. , Caughlin, T. T. , zu Erbach‐Schoenberg, E. , Lourenço, C. , Ruktanonchai, C. W. , & Smith, D. L. (2016). Identifying malaria transmission foci for elimination using human mobility data. PLOS Computational Biology, 12, e1004846. 27043913 10.1371/journal.pcbi.1004846 PMC4820264

[cl21149-bib-0342] Saha, M. , & Eckelman, M. J. (2017). Growing fresh fruits and vegetables in an urban landscape: A geospatial assessment of ground level and rooftop urban agriculture potential in Boston, USA. Landscape and Urban Planning, 165, 130–141. 10.1016/j.landurbplan.2017.04.015

[cl21149-bib-0343] Salhab, J. , Wang, J. J. , Anjum, S. A. , & Chen, Y. X. (2010). Assessment of the grassland degradation in the southeastern part of the source region of the Yellow River from 1994 to 2001. Journal of Food, Agriculture and Environment, 8, 1367–1372.

[cl21149-bib-0344] Sanga‐Ngoie, K. , Iizuka, K. , & Kobayashi, S. (2012). Estimating CO_2_ sequestration by forests in Oita Prefecture, Japan, by combining LANDSAT ETM+ and ALOS satellite remote sensing data. Remote Sensing, 4, 3544–3570.

[cl21149-bib-0345] Sani, N. A. , Kafaky, S. B. , Pukkala, T. , & Mataji, A. (2016). Integrated use of GIS, remote sensing and multi‐criteria decision analysis to assess ecological land suitability in multi‐functional forestry. Journal of Forestry Research, 27, 1127–1135.

[cl21149-bib-0346] Saher, F. N. , Nasly, M. A. , Kadir, T. A. B. A. , Yahaya, N. K. E. , & Ishak, W. M. F. W. (2014). Harnessing floodwater of hill torrents for improved spate irrigation system using geo‐informatics approach. Research Journal of Recent Sciences, 3, 14–22.

[cl21149-bib-0347] Schneider, A. , Mertes, C. M. , Tatem, A. J. , Tan, B. , Sulla‐Menashe, D. , Graves, S. J. , Patel, N. N. , Horton, J. A. , Gaughan, A. E. , Rollo, J. T. , Schelly, I. H. , Stevens, F. R. , & Dastur, A. (2015). A new urban landscape in East–Southeast Asia, 2000–2010. Environmental Research Letters, 10, 034002. 10.1088/1748-9326/10/3/034002

[cl21149-bib-0348] Scholte, R. G. C. , Freitas, C. C. , Dutra, L. V. , Guimaraes, R. J. P. S. , Drummond, S. C. , Oliveira, G. , & Carvalho, O. S. (2012). Utilizing environmental, socioeconomic data and GIS techniques to estimate the risk for ascariasis and trichuriasis in Minas Gerais, Brazil. Acta Tropica, 121, 112–117. 22041638 10.1016/j.actatropica.2011.10.011

[cl21149-bib-0349] Sedda, L. , Tatem, A. J. , Morley, D. W. , Atkinson, P. M. , Wardrop, N. A. , Pezzulo, C. , Sorichetta, A. , Kuleszo, J. , & Rogers, D. J. (2015). Poverty, health and satellite‐derived vegetation indices: Their inter‐spatial relationship in West Africa. International Health, 7, 99–106. 10.1093/inthealth/ihv005 25733559 PMC4357798

[cl21149-bib-0350] See, L. , McCallum, I. , Fritz, S. , Perger, C. , Kraxner, F. , Obersteiner, M. , Baruah, U. D. , Nitashree, M. , & Kalita, N. R. (2013). Mapping cropland in Ethiopia using crowdsourcing. International Journal of Geosciences, 4, 6–13.

[cl21149-bib-0351] Seitz, N. E. , Westbrook, C. J. , Dubé, M. G. , & Squires, A. J. (2013). Assessing large spatial scale landscape change effects on water quality and quantity response in the lower Athabasca River basin. Integrated Environmental Assessment and Management, 9, 392–404. 10.1002/ieam.1336 22778001

[cl21149-bib-0352] Shadumyan, H. (2006). *Application of geographic information systems (GIS) Armenia: Opportunities for expanded use in the health sphere*. https://bit.ly/3nIakvW

[cl21149-bib-0353] Shanableh, A. , Al‐Ruzouq, R. , Yilmaz, A. G. , Siddique, M. , Merabtene, T. , & Imteaz, M. A. (2018). Effects of land cover change on urban floods and rainwater harvesting: A case study in Sharjah, UAE. Water, 10, 631.

[cl21149-bib-0354] Shomar, B. , Fakher, S. A. , & Yahya, A. (2010). Assessment of groundwater quality in the Gaza Strip, Palestine using GIS mapping. Journal of Water Resource and Protection, 2, 93–104.

[cl21149-bib-0355] Shukla, A. , & Jain, K. (2019). Critical analysis of rural‐urban transitions and transformations in Lucknow city, India. Remote Sensing Applications: Society and Environment, 13, 445–456. 10.1016/j.rsase.2019.01.001

[cl21149-bib-0356] Siddiqui, Z. (2011). Holistic approach to mitigate the pollution impacts in the coastal ecosystem of Thailand using the remote sensing techniques. International Journal of Environmental Research, 5, 297–306.

[cl21149-bib-0357] Singh, R. (2005). *Water productivity analysis from field to regional scale: Integration of crop and soil modelling, remote sensing and geographical information*. Wageningen; the Netherlands.

[cl21149-bib-0358] Singh, S. , & Yassine, A. (2018). Big data mining of energy time series for behavioral analytics and energy consumption forecasting. Energies, 11(2), 452.

[cl21149-bib-0359] Siyal, A. A. , Misrani, D. M. , Dars, G. H. , & Ahmad, S. (2018, May). *Application of GIS and remote sensing for identification of potential runoff harvesting sites: A case study of Karoonjhar Mountainous Area, Pakistan*. World Environmental and Water Resources Congress 2018: International Perspectives, History and Heritage, Emerging Technologies, and Student Papers (pp. 20–33). Reston, VA: American Society of Civil Engineers.

[cl21149-bib-0360] Sofeska, E. (2017). Understanding the livability in a city through smart solutions and urban planning toward developing sustainable livable future of the City of Skopje. Procedia Environmental Sciences, 37, 442–453. 10.1016/j.proenv.2017.03.014

[cl21149-bib-0361] Song, J. , Zhao, C. , Lin, T. , Li, X. , & Prishchepov, A. V. (2019). Spatio‐temporal patterns of traffic‐related air pollutant emissions in different urban functional zones estimated by real‐time video and deep learning technique. Journal of Cleaner Production, 238. 10.1016/j.jclepro.2019.117881

[cl21149-bib-0362] Soytong, P. , Janchidfa, K. , Phengphit, N. , Chayhard, S. , & Perera, R. (2016). The effects of land use change and climate change on water resources in the eastern region of Thailand. International Journal of Agricultural Technology, 12, 1697–1724.

[cl21149-bib-0363] Steele, J. E. , Sundsøy, P. R. , Pezzulo, C. , Alegana, V. A. , Bird, T. J. , Blumenstock, J. , Bjelland, J. , Engø‐Monsen, K. , de Montjoye, Y.‐A. , Iqbal, A. M. , Hadiuzzaman, K. N. , Lu, X. , Wetter, E. , Tatem, A. J. , & Bengtsson, L. (2017). Mapping poverty using mobile phone and satellite data. Journal of the Royal Society Interface, 14, 20160690. 10.1098/rsif.2016.0690 28148765 PMC5332562

[cl21149-bib-0364] Stein, C. , Ernstson, H. , & Barron, J. (2011). A social network approach to analyzing water governance: The case of the Mkindo catchment, Tanzania. Physics and Chemistry of the Earth, 36, 1085–1092. 10.1016/j.pce.2011.07.083

[cl21149-bib-0365] Stergiadou, A. , Libello, D. , Cavalli, R. , & Krč, J. (2009). Estimating forest harvesting operations to achieve sustainable rural development in Samarina (Greece). Folia Forestalia Polonica. Seria A, Leśnictwo, 51, 21–28.

[cl21149-bib-0366] Stevens, F. R. , Gaughan, A. E. , Linard, C. , & Tatem, A. J. (2015). Disaggregating census data for population mapping using random forests with remotely‐sensed and ancillary data. PLOS One, 10, e0107042. 25689585 10.1371/journal.pone.0107042 PMC4331277

[cl21149-bib-0367] Sturrock, H. J. W. , Cohen, J. M. , Keil, P. , Tatem, A. J. , Le Menach, A. , Ntshalintshali, N. E. , Hsiang, M. S. , & Gosling, R. D. (2014). Fine‐scale malaria risk mapping from routine aggregated case data. Malaria Journal, 13, 421. 10.1186/1475-2875-13-421 25366929 PMC4349235

[cl21149-bib-0368] Subasinghe, S. , Estoque, R. C. , & Murayama, Y. (2016). Spatiotemporal analysis of urban growth using GIS and remote sensing: A case study of the Colombo Metropolitan Area, Sri Lanka. ISPRS International Journal of Geo‐Information, 5, 197.

[cl21149-bib-0369] Sundsøy, P. (2016). Can mobile usage predict illiteracy in a developing country? arXiv, 1607, 01337. https://arxiv.org/ftp/arxiv/papers/1607/1607.01337.pdf

[cl21149-bib-0370] Sutton, P. , Elvidge, C. , & Ghosh, T. (2007). Estimation of gross domestic product at sub‐national scales using nighttime satellite imagery. International Journal of Ecological Economics and Statistics, 8, S07.

[cl21149-bib-0371] Tanaka, K. , & Keola, S. (2017). Shedding light on the shadow economy: A nighttime light approach. Journal of Development Studies, 53, 32–48. 10.1080/00220388.2016.1171845

[cl21149-bib-0372] Taramelli, A. , Valentini, E. , & Sterlacchini, S. (2015). A GIS‐based approach for hurricane hazard and vulnerability assessment in the Cayman Islands. Ocean & Coastal Management, 108, 116–130.

[cl21149-bib-0373] Tatem, A. J. , Huang, Z. , Narib, C. , Kumar, U. , Kandula, D. , Pindolia, D. K. , Smith, D. L. , Cohen, J. M. , Graupe, B. , Uusiku, P. , & Lourenço, C. (2014). Integrating rapid risk mapping and mobile phone call record data for strategic malaria elimination planning. Malaria Journal, 13, 52. 10.1186/1475-2875-13-52 24512144 PMC3927223

[cl21149-bib-0374] Tatem, A. J. , Qiu, Y. , Smith, D. L. , Sabot, O. , Ali, A. S. , & Moonen, B. (2009). The use of mobile phone data for the estimation of the travel patterns and imported *Plasmodium falciparum* rates among Zanzibar residents. Malaria Journal, 8, 287. 10.1186/1475-2875-8-287 20003266 PMC2800118

[cl21149-bib-0375] Teerayut, H. (2010). *A study on urban mobility and dynamic population estimation by using aggregate mobile phone sources* (Doctoral dissertation, University of Tokyo). https://bit.ly/3nHA7nM

[cl21149-bib-0376] Thomson, D. R. , Kools, L. , & Jochem, W. C. (2018). Linking synthetic populations to household geolocations: A demonstration in Namibia. Data, 3, 30. 10.3390/data3030030

[cl21149-bib-0377] Tian, J. , Zhao, N. , Samson, E. L. , & Wang, S. (2014). Brightness of nighttime lights as a proxy for freight traffic: A case study of China. IEEE Journal of Selected Topics in Applied Earth Observations and Remote Sensing, 7, 206–212. 10.1109/JSTARS.2013.2258892

[cl21149-bib-0378] Toole, J. L. , Lin, Y.‐R. , Muehlegger, E. , Shoag, D. , González, M. C. , & Lazer, D. (2015). Tracking employment shocks using mobile phone data. Journal of the Royal Society, Interface, 12, 107. 10.1098/rsif.2015.0185 PMC4590504 26018965

[cl21149-bib-0379] Trigg, S. N. , Curran, L. M. , & McDonald, A. K. (2006). Utility of Landsat 7 satellite data for continued monitoring of forest cover change in protected areas in Southeast Asia. Singapore Journal of Tropical Geography, 27, 49–66. 10.1111/j.1467-9493.2006.00239.x

[cl21149-bib-0380] Tzavella, K. , Fekete, A. , & Fiedrich, F. (2018). Opportunities provided by geographic information systems and volunteered geographic information for a timely emergency response during flood events in Cologne, Germany. Natural Hazards, 29, 10.1007/s11069-017-3102-1

[cl21149-bib-0381] Uddin, K. , Murthy, M. S. R. , Wahid, S. M. , & Matin, M. A. (2016). Estimation of soil erosion dynamics in the Koshi basin using GIS and remote sensing to assess priority areas for conservation. PLOS One, 11(3), e0150494. 26964039 10.1371/journal.pone.0150494 PMC4786292

[cl21149-bib-0382] USAID . (2018). *Real‐time monitoring for improved water services in the Ethiopian Lowlands*. https://bit.ly/3lQ9vjU

[cl21149-bib-0383] Utazi, C. E. , Thorley, J. , Alegana, V. A. , Ferrari, M. J. , Takahashi, S. , Metcalf, C. J. E. , Lessler, J. , & Tatem, A. J. (2018). High resolution age‐structured mapping of childhood vaccination coverage in low and middle income countries. Vaccine, 36, 1583–1591. 10.1016/j.vaccine.2018.02.020 29454519 PMC6344781

[cl21149-bib-0384] Valjarević, A. , Djekić, T. , Stevanović, V. , Ivanović, R. , & Jandziković, B. (2018). GIS numerical and remote sensing analyses of forest changes in the Toplica region for the period of 1953–2013. Applied Geography, 92, 131–139. 10.1016/j.apgeog.2018.01.016

[cl21149-bib-0385] Van Beijma, S. , Chatterton, J. , Page, S. , Rawlings, C. , Tiffin, R. , & King, H. (2018). The challenges of using satellite data sets to assess historical land use change and associated greenhouse gas emissions: A case study of three Indonesian provinces. Carbon Management, 9, 399–413. 10.1080/17583004.2018.1511383

[cl21149-bib-0386] Varshney, K. R. , Chen, G. H. , Abelson, B. , Nowocin, K. , Sakhrani, V. , Xu, L. , & Spatocco, B. L. (2015). Targeting villages for rural development using satellite image analysis. Big Data, 3, 41–53. 10.1089/big.2014.0061 27442844

[cl21149-bib-0387] Viña, A. , Chen, X. , McConnell, W. J. , Liu, W. , Xu, W. , Ouyang, Z. , Zhang, H. , & Liu, J. (2011). Effects of natural disasters on conservation policies: The case of the 2008 Wenchuan Earthquake, China. Ambio, 40, 274–284. 21644456 10.1007/s13280-010-0098-0 PMC3357801

[cl21149-bib-0388] Vogel, K.B. , Goldblatt, R. , Hanson, G.H. , & Khandelwal, A.K. (2018). *Detecting urban markets with satellite imagery: An application to India*. National Bureau of Economic Research Working Paper Series No. 24796. 10.3386/w24796

[cl21149-bib-0389] Wahyu, A.N. , Hidayah, Z. , & Insafitri . (2013). *Developing Coral Reef Conservation Zones in the Kangean Archipelago, Indonesia*.

[cl21149-bib-0390] Wang, A. X. , Tran, C. , Desai, N. , Lobell, D. , & Ermon, S. (2018, June). *Deep transfer learning for crop yield prediction with remote sensing data*. Proceedings of the 1st ACM SIGCAS Conference on Computing and Sustainable Societies (pp. 1–5).

[cl21149-bib-0391] Wang, L. Y. , Fan, H. , & Wang, Y. K. (2019). Fine‐resolution population mapping from international space station nighttime photography and multisource social sensing data based on similarity matching. Remote Sensing, 11(16), 1900. 10.3390/rs11161900

[cl21149-bib-0392] Wang, S. , Azzari, G. , & Lobell, D. B. (2019). Crop type mapping without field‐level labels: Random forest transfer and unsupervised clustering techniques. Remote Sensing of Environment, 222, 303–317. 10.1016/j.rse.2018.12.026

[cl21149-bib-0393] Wang, S. , Chen, W. , Xie, S. M. , Azzari, G. , & Lobell, D. B. (2020). Weakly supervised deep learning for segmentation of remote sensing imagery. Remote Sensing, 12(2), 207. 10.3390/rs12020207

[cl21149-bib-0394] Weinzierl, T. , Wehberg, J. , Böhner, J. , & Conrad, O. (2016). Spatial assessment of land degradation risk for the Okavango River catchment, southern Africa. Land Degradation and Development, 27, 281–294.

[cl21149-bib-0395] Weng, Q. , Fu, P. , & Gao, F. (2014). Generating daily land surface temperature at Landsat resolution by fusing Landsat and MODIS data. Remote Sensing of Environment, 145, 55–67. 10.1016/j.rse.2014.02.003

[cl21149-bib-0396] Wesolowski, A. , Buckee, C. O. , Bengtsson, L. , Wetter, E. , Lu, X. , & Tatem, A. J. (2014a). Commentary: Containing the Ebola outbreak—The potential and challenge of mobile network data. PLOS Currents, 6, 10.1371/currents.outbreaks.0177e7fcf52217b8b634376e2f3efc5e PMC4205120 25642369

[cl21149-bib-0397] Wesolowski, A. , Buckee, C. O. , Pindolia, D. K. , Eagle, N. , Smith, D. L. , Garcia, A. J. , & Tatem, A. J. (2013a). The use of census migration data to approximate human movement patterns across temporal scales. PLOS One, 8, e52971. 23326367 10.1371/journal.pone.0052971 PMC3541275

[cl21149-bib-0398] Wesolowski, A. , Eagle, N. , Noor, A. M. , Snow, R. W. , & Buckee, C. O. (2013b). The impact of biases in mobile phone ownership on estimates of human mobility. Journal of the Royal Society Interface, 10, 20120986. 10.1098/rsif.2012.0986 23389897 PMC3627108

[cl21149-bib-0399] Wesolowski, A. , Eagle, N. , Tatem, A. J. , Smith, D. L. , Noor, A. M. , Snow, R. W. , & Buckee, C. O. (2012). Quantifying the impact of human mobility on malaria. Science, 338, 267–270. 10.1126/science.1223467 23066082 PMC3675794

[cl21149-bib-0400] Wesolowski, A. , Metcalf, C. J. E. , Eagle, N. , Kombich, J. , Grenfell, B. T. , Bjørnstad, O. N. , Lessler, J. , Tatem, A. J. , & Buckee, C. O. (2015). Quantifying seasonal population fluxes driving rubella transmission dynamics using mobile phone data. Proceedings of the National Academy of Sciences of the United States of America, 112, 11114–11119. 10.1073/pnas.1423542112 26283349 PMC4568255

[cl21149-bib-0401] Wesolowski, A. , O'Meara, W. P. , Tatem, A. J. , Ndege, S. , Eagle, N. , & Buckee, C. O. (2015). Quantifying the impact of accessibility on preventive healthcare in sub‐Saharan Africa using mobile phone data. Epidemiology (Cambridge, Mass.), 26(2), 223–228. 25643101 10.1097/EDE.0000000000000239 PMC4323566

[cl21149-bib-0402] Wesolowski, A. , Stresman, G. , Eagle, N. , Stevenson, J. , Owaga, C. , Marube, E. , Bousema, T. , Drakeley, C. , Cox, J. , & Buckee, C. O. (2014b). Quantifying travel behavior for infectious disease research: A comparison of data from surveys and mobile phones. Scientific Reports, 4, 5678. 10.1038/srep05678 25022440 PMC4894426

[cl21149-bib-0403] Wey, W.‐M. , & Huang, J.‐Y. (2018). Urban sustainable transportation planning strategies for livable City's quality of life. Habitat International, 82, 9–27. 10.1016/j.habitatint.2018.10.002

[cl21149-bib-0404] Wilson, R. , zu Erbach‐Schoenberg, E. , Albert, M. , Power, D. , Tudge, S. , Gonzalez, M. , Guthrie, S. , Chamberlain, H. , Brooks, C. , Hughes, C. , Pitonakova, L. , Buckee, C. , Lu, X. , Wetter, E. , Tatem, A. , & Bengtsson, L. (2016). Rapid and near real‐time assessments of population displacement using mobile phone data following disasters: The 2015 Nepal Earthquake. PLOS Currents, 8. 10.1371/currents.dis.d073fbece328e4c39087bc086d694b5c PMC4779046 26981327

[cl21149-bib-0405] Witmer, F. , & O'Loughlin, J. (2011). Detecting the effects of wars in the caucasus regions of Russia and Georgia using radiometrically normalized DMSP‐OLS nighttime lights imagery. GIScience and Remote Sensing, 48, 478–500. 10.2747/1548-1603.48.4.478

[cl21149-bib-0406] Wondrade, N. , Øystein, D. , & Havard, T. (2014). GIS based mapping of land cover changes utilizing multi‐temporal remotely sensed image data in Lake Hawassa Watershed, Ethiopia. Environmental Monitoring and Assessment, 186, 1765–1780. 10.1007/s10661-013-3491-x 24310365

[cl21149-bib-0407] Wong, M. S. , Zhu, R. , Liu, Z. , Lu, L. , Peng, J. , Tang, Z. , Lo, X. H. , & Chan, W. K. (2016). Estimation of Hong Kong's solar energy potential using GIS and remote sensing technologies. Renewable Energy, 99, 325–335. 10.1016/j.renene.2016.07.003

[cl21149-bib-0408] Wu, W. , Ren, H. Y. , Yu, M. , & Wang, Z. (2018). Distinct influences of urban villages on urban heat islands: A case study in the Pearl River Delta, China. International Journal of Environmental Research and Public Health, 15, 1666. 10.3390/ijerph15081666 30082641 PMC6121422

[cl21149-bib-0409] Xia, T. Q. , Song, X. , Zhang, H. R. , Song, X. Y. , Kanasugi, H. , & Shibasaki, R. (2019). Measuring spatio‐temporal accessibility to emergency medical services through big GPS data. Health and Place, 56, 53–62. 10.1016/j.healthplace.2019.01.012 30703630

[cl21149-bib-0410] Xie, H. , He, Y. , & Xie, X. (2017). Exploring the factors influencing ecological land change for China's Beijing–Tianjin–Hebei Region using big data. Journal of Cleaner Production, 142, 677–687. 10.1016/j.jclepro.2016.03.064

[cl21149-bib-0411] Xu, Q. N. , Gel, Y. R. , Ramirez‐Ramirez, L. L. , Nezafati, K. , Zhang, Q. P. , & Tsui, K. L. (2017). Forecasting influenza in Hong Kong with Google search queries and statistical model fusion. PLOS One, 12, e0176690. 28464015 10.1371/journal.pone.0176690 PMC5413039

[cl21149-bib-0412] Xu, W. , Zheng, T. , & Li, Z. (2011). *A neural network based forecasting method for the unemployment rate prediction using the search engine query data*. The 2011 IEEE 8th International Conference on e‐Business Engineering (pp. 9–15). 10.1109/ICEBE.2011.21

[cl21149-bib-0413] Xu, Y. , Jiang, S. , Li, R. , Zhang, J. , Zhao, J. , Abbar, S. , & González, M. C. (2019). Unraveling environmental justice in ambient PM2.5 exposure in Beijing: A big data approach. Computers, Environment and Urban Systems, 75, 12–21. 10.1016/j.compenvurbsys.2018.12.006

[cl21149-bib-0414] Yamagata, Y. , Yoshida, T. , Murakami, D. , Matsui, T. , & Akiyama, Y. (2018). Seasonal urban carbon emission estimation using spatial micro big data. Sustainability, 10, 1.

[cl21149-bib-0415] Yang, R. , Luo, Y. , Yang, K. , Hong, L. , & Zhou, X. (2019). Analysis of forest deforestation and its driving factors in Myanmar from 1988 to 2017. Sustainability, 11, 1.

[cl21149-bib-0416] Yang, S. , Santillana, M. , & Kou, S. C. (2015). Accurate estimation of influenza epidemics using Google search data via ARGO. Proceedings of the National Academy of Sciences of the United States of America, 112, 14473–14478. 10.1073/pnas.1515373112 26553980 PMC4664296

[cl21149-bib-0417] Yang, X. H. , Smith, P. L. , Yu, T. , & Gao, H. L. (2011). Estimating evapotranspiration from terrestrial groundwater‐dependent ecosystems using Landsat images. International Journal of Digital Earth, 4, 154–170.

[cl21149-bib-0418] Yang, Y. , Anderson, M. C. , Gao, F. , Hain, C. R. , Semmens, K. A. , Kustas, W. P. , Noormets, A. , Wynne, R. H. , Thomas, V. A. , & Sun, G. (2017). Daily landsat‐scale evapotranspiration estimation over a forested landscape in North Carolina, USA, using multi‐satellite data fusion. Hydrology and Earth System Sciences, 21, 1017–1037.

[cl21149-bib-0419] Yin, J. , Yin, Z. , Zhong, H. , Xu, S. , Hu, X. , Wang, J. , & Wu, J. (2011). Monitoring urban expansion and land use/land cover changes of Shanghai metropolitan area during the transitional economy (1979–2009) in China. Environmental Monitoring and Assessment, 177, 609–621. 10.1007/s10661-010-1660-8 20824336

[cl21149-bib-0420] Zabala, A. (2013). Comparing global spatial data on deforestation for institutional analysis in Africa. Reference Module in Earth Systems and Environmental Sciences, 10.1016/B978-0-12-409548-9.09681-0

[cl21149-bib-0421] Zafar, S. , & Zaidi, A. (2019). Impact of urbanization on basin hydrology: A case study of the Malir Basin, Karachi, Pakistan. Regional Environmental Change, 19, 1815–1827. 10.1007/s10113-019-01512-9

[cl21149-bib-0422] Zagatti, G. A. , Gonzalez, M. , Avner, P. , Lozano‐Gracia, N. , Brooks, C. J. , Albert, M. , Gray, J. , Antos, S. E. , Burci, P. , zu Erbach‐Schoenberg, E. , Tatem, A. J. , Wetter, E. , & Bengtsson, L. (2018). A trip to work: Estimation of origin and destination of commuting patterns in the main metropolitan regions of Haiti using CDR. Development Engineering, 3, 133–165. 10.1016/j.deveng.2018.03.002

[cl21149-bib-0423] Zhang, C. Z. , Cai, J. H. , Xiao, D. Q. , Ye, Y. W. , & Chehelamirani, M. (2018). Research on vegetable pest warning system based on multidimensional big data. Insects, 9, 66. 29899312 10.3390/insects9020066 PMC6023421

[cl21149-bib-0424] Zhang, H. , Song, X. , Long, Y. , Xia, T. , Fang, K. , Zheng, J. , Huang, D. , Shibasaki, R. , & Liang, Y. (2019). Mobile phone GPS data in urban bicycle‐sharing: Layout optimization and emissions reduction analysis. Applied Energy, 242, 138–147.

[cl21149-bib-0425] Zhang, J. H. , Yao, F. M. , Liu, C. , Yang, L. M. , & Boken, V. K. (2011). Detection, emission estimation and risk prediction of forest fires in China using satellite sensors and simulation models in the past three decades—An overview. International Journal of Environmental Research and Public Health, 8, 3156–3178. 21909297 10.3390/ijerph8083156 PMC3166733

[cl21149-bib-0426] Zhang, P. C. , Zhao, Q. , Gao, J. , Li, W. R. , & Lu, J. M. (2019). Urban street cleanliness assessment using mobile edge computing and deep learning. IEEE Access, 7, 63550–63563. 10.1109/access.2019.2914270

[cl21149-bib-0427] Zhang, R. , Su, H. , Tian, J. , Li, Z. , Chen, S. , Zhan, J. , Deng, X. , Sun, X. , & Wu, J. (2008). Drought monitoring in Northern China based on remote sensing data and land surface modeling. *The IGARSS 2008–2008 IEEE International Geoscience and Remote Sensing Symposium*, pp. III–860. 10.1109/IGARSS.2008.4779485

[cl21149-bib-0428] Zhang, X. , Chen, J. , Tan, M. , & Sun, Y. (2007). Assessing the impact of urban sprawl on soil resources of Nanjing city using satellite images and digital soil databases. Catena, 69, 16–30. 10.1016/j.catena.2006.04.020

[cl21149-bib-0429] Zhang, X. , Fu, J. Y. , Lin, G. , Jiang, D. , & Yan, X. X. (2017). Switchgrass‐based bioethanol productivity and potential environmental impact from marginal lands in China. Energies, 10, 260.

[cl21149-bib-0430] Zhang, Y. , Yang, J. , He, H. S. , Dijak, W. D. , Shifley, S. R. , & Palik, B. J. (2009). Integration of satellite imagery and forest inventory in mapping dominant and associated species at a regional scale. Environmental Management, 44, 312–323. 10.1007/s00267-009-9307-7 19488811

[cl21149-bib-0431] Zheng, H. , Hong, Y. , Long, D. , & Jing, H. (2017). Monitoring surface water quality using social media in the context of citizen science. Hydrology and Earth System Sciences, 21, 949–961.

[cl21149-bib-0432] Zheng, X. , Zhu, J. , & Yan, Q. (2013). Monthly air temperatures over Northern China Estimated By Integrating MODIS data with GIS techniques. Journal of Applied Meteorology and Climatology, 52, 1987–2000.

[cl21149-bib-0433] Zhou, D. M. , Radke, J. , Tian, Y.‐Q. , Xu, J.‐C. , & Mu, L. (2005). A model supported by GIS for locating and quantifying PM_{2.5} emission originated from residential wood burning. Journal of Environmental Sciences (IOS Press), 17, 861–865. 16313020

[cl21149-bib-0434] Živković, L. , & Đorđević, A. (2016). Building a GIS platform for sustainable land management: A case study of the City of Čačak, Serbia. Journal of Urban Technology, 23, 29–46. 10.1080/10630732.2015.1102420

[cl21149-bib-0435] Zonghao, R. , Yang, D. , & Duan, Z. (2013). Resident mobility analysis based on mobile‐phone billing data. Procedia—Social and Behavioral Sciences, 96, 2032–2041. 10.1016/j.sbspro.2013.08.229

[cl21149-bib-0436] Zope, P. E. , Eldho, T. I. , & Jothiprakash, V. (2017). Hydrological impacts of land use–land cover change and detention basins on urban flood hazard: A case study of Poisar River basin, Mumbai, India. Natural Hazards, 87, 1267–1283. 10.1007/s11069-017-2816-4

[cl21149-bib-0437] Zu Erbach‐Schoenberg, E. , Alegana, V. A. , Sorichetta, A. , Linard, C. , Lourenço, C. , Ruktanonchai, N. W. , Graupe, B. , Bird, T. J. , Pezzulo, C. , Wesolowski, A. , & Tatem, A. J. (2016). Dynamic denominators: The impact of seasonally varying population numbers on disease incidence estimates. Population health metrics, 14, 35. 10.1186/s12963-016-0106-0 27777514 PMC5062910

[cl21149-bib-0438] Archie, M. , Gershon, S. , Katcoff, A. , & Zeng, A. (2018). Who's watching? De‐anonymization of Netflix reviews using Amazon reviews, https://bit.ly/2HjFaLR

[cl21149-bib-0439] Bamberger, M. (2018). Dealing with complexity in development evaluation: A practical approach. Sage Publications.

[cl21149-bib-0440] BenYishay, A. , Trichler, R. , Runfola, D. , & Goodman, S. (2018). Final report: Evaluation of the infrastructure needs program II. AidData at William & Mary.

[cl21149-bib-0441] Blondel, V. D. , Decuyper, A. , & Krings, G. (2015). A survey of results on mobile phone datasets analysis. EPJ Data Science, 4(1), 10.

[cl21149-bib-0442] Blumenstock, J. , Cadamuro, G. , & On, R. (2015). Predicting poverty and wealth from mobile phone metadata. Science, 350(6264), 1073–1076. 26612950 10.1126/science.aac4420

[cl21149-bib-0443] Blazquez, D. , & Domenech, J. (2018). Big data sources and methods for social and economic analyses. Technological Forecasting & Social Change, 130, 99–113.

[cl21149-bib-0444] Burke, M. , & Lobell, D. (2017). Satellite‐based assessment of African yields. Proceedings of the National Academy of Sciences of the United States of America, 114(9), 2189–2194. 10.1073/pnas.1616919114 28202728 PMC5338538

[cl21149-bib-0445] Carrera, F. , Guerin, S. , & Thorp, J. (2013). By the people, for the people: The crowdsourcing of “streetbump”: An automatic pothole mapping app. International Archives of the Photogrammetry, Remote Sensing and Spatial Information Sciences, XL‐4/W1, 19–23. 10.5194/isprsarchives-XL-4-W1-19-2013

[cl21149-bib-0446] Chernozhukov, V. , Demirer, M. , Duflo, E. , & Fernadezval, I. (2019). Generic machine learning inference on heterogeneous treatment effects in randomized experiments. arXiv:1712.04802v4.

[cl21149-bib-0447] Decuyper, A. , Rutherford, A. , Wadhwa, A. , Bauer, J. M. , Krings, G. , Gutierrez, T. , & Luengo‐Oroz, A. (2014). Estimating food consumption and poverty indices with mobile phone data. CoRR. abs/1412.2595.

[cl21149-bib-0448] DFID . (2016). *Memorandum by the Department for International Development*. http://data.parliament.uk/writtenevidence/committeeevidence.svc/evidencedocument/international-development-committee/dfids-allocation-of-resources/written/28276.pdf

[cl21149-bib-0449] Edjekumhene, I. , Voors, M. , Lujala, P. , Brunnschweiler, C. , Owusu, C. K. , & Nyamekye, A. (2019). Impacts of key provisions in Ghana's Petroleum Revenue Management Act, 3ie Impact Evaluation Report 94. New Delhi: International Initiative for Impact Evaluation (3ie). 10.23846/TW8IE94

[cl21149-bib-0450] Fu, H. (2019). *Data for development impact: Why we need to invest in data, people and ideas*. https://blogs.worldbank.org/voices/data-for-development-impact-why-we-need-to-invest-in-data-people-and-ideas

[cl21149-bib-0451] Gaarder, M. , & Annan, J. (2013). *Impact evaluation of conflict prevention and peacebuilding interventions*. The World Bank Policy Research Working Paper 6496. http://documents.worldbank.org/curated/en/445741468177842482/pdf/WPS6496.pdf

[cl21149-bib-0452] Gerber, M. S. (2014). Predicting crime using Twitter and kernel density estimation. Decision Support Systems, 61, 115–125.

[cl21149-bib-0453] Hammer, C. , Kostroch, D. , & Quiros, G. (2017). Big data: Potential, challenges and statistical implications. Staff Discussion Notes, 17, 1. 10.5089/9781484310908.006

[cl21149-bib-0454] Hansen, M. C. , Potapov, P. V. , Moore, R. , Hancher, M. , Turubanova, S. A. , Tyukavina, A. , Thau, D. , Stehman, S. V. , Goetz, S. J. , Loveland, T. R. , Kommareddy, A. , Egorov, A. , Chini, L. , Justice, C. O. , & Townshend, J. R. G. (2013). High‐resolution global maps of 21st‐century forest cover change. Science, 342(6160), 850–853. 24233722 10.1126/science.1244693

[cl21149-bib-0455] Head, A. , Manguin, M. , Tran, N. , & Blumenstock, J. E (2017, November 16–19). “Can human development be measured with satellite imagery?” The Ninth International Conference, ICTD.Pakistan.

[cl21149-bib-0456] Horowitz, M. C. , Allen, G. C. , Saravalle, E. , Cho, A. , Frederick, K. , & Scharre, P. (2018). Artificial intelligence and international security. Center for a New American Security.

[cl21149-bib-0457] Jain, M. (2020). The benefits and pitfalls of using satellite data for causal inference. Review of Environmental Economics and Policy, 14(1), 157–169. 10.1093/reep/rez023

[cl21149-bib-0458] Jaiswal, S. , Bensch, G. , Navalkar, A. , Jayaraman, T. , Murari, K. , & Patnaik, U. (2020). Evaluating the impact of infrastructure development: case study of the Konkan Railway in India, 3ie Impact Evaluation Report 114. New Delhi: International Initiative for Impact Evaluation (3ie). 10.23846/DPW1IE114

[cl21149-bib-0459] Jayachandran, S. , de Laat, J. , Lambin, E. F. , Stanton, C. Y. , Audy, R. , & Thomas, N. E. (2017). Cash for carbon: A randomized trial of payments for ecosystem services to reduce deforestation. Science, 357(6348), 267–273. 28729505 10.1126/science.aan0568

[cl21149-bib-0460] Jean, N. , Burke, M. , Xie, M. , Davis, W. M. , Lobell, D. B. , & Ermon, S. (2016). Combining satellite imagery and machine learning to predict poverty. Science, 353(6301), 790–794. 27540167 10.1126/science.aaf7894

[cl21149-bib-0461] Letouzé, E. (2016). *Big data and development: An overview*. https://datapopalliance.org/item/white-paper-series-official-statistics-big-data-and-human-development/

[cl21149-bib-0462] Lokanathan, S. , Gomez, T. , & Zuhyle, S. (2017). Mapping big data solutions for the sustainable development goals. LIRNEasia.

[cl21149-bib-0463] Lu, X. , Bengtsson, L. , & Holme, P. (2012). Predictability of population displacement after the 2010 Haiti earthquake. Proceedings of the National Academy of Sciences of the United States of America, 109(29), 11576–11581. 22711804 10.1073/pnas.1203882109 PMC3406871

[cl21149-bib-0464] Manyika, J. , Chui, M. , Brown, B. , Bughin, J. , Dobbs, R. , Roxburgh, C. , & Byers, A. H. (2011). Big data: The next frontier for innovation, competition, and productivity. McKinsey & Company.

[cl21149-bib-0465] Olteanu, A. , Castillo, C. , Diaz, F. , & Kıcıman, E. (2019). Social data: Biases, methodological pitfalls, and ethical boundaries. Frontiers in Big Data, 2, 13. 10.3389/fdata.2019.00013 33693336 PMC7931947

[cl21149-bib-0466] Osorio, J. (2014). *Numbers under fire: The challenges of gathering quantitative data in highly violent settings*. Social Science Research Council, Drugs, Security and Democracy Program (DSD) Working Papers on Research Security 6.

[cl21149-bib-0467] Pande, R. , & Sudarshan, A. (2019). *Harnessing transparency initiatives to improve India's environmental clearance process for the mineral mining sector*. 3ie Impact Evaluation Report 92. New Delhi: International Initiative for Impact Evaluation (3ie). 10.23846/TW8IE92

[cl21149-bib-0468] Pellegrini, L. (2019). Community monitoring of socio‐environmental liabilities with advanced technologies in the Ecuadorian and Peruvian Amazon, 3ie Grantee Final Report. New Delhi: International Initiative for Impact Evaluation (3ie).

[cl21149-bib-0469] Pelletier, J. , Gélinas, N. , & Skutsch, M. (2016). The place of community forest management in the REDD+ landscape. Forests, 2016(7), 170.

[cl21149-bib-0470] Perera Gomez, T. , & Lokanathan, S. (2017). Leveraging big data to support measurement of the sustainable development goals. SSRN Electronic Journal, 10.2139/ssrn.3058530

[cl21149-bib-0471] Phillips, D. , Coffey, C. , Tsoli, S. , Stevenson, J. , Waddington, H. , Eyers, J. , White, H. , & Snilstveit, B. (2017). *A map of evidence maps relating to sustainable development in low‐ and middle income countries evidence gap map report*. CEDIL Pre‐Inception Paper.

[cl21149-bib-0472] Puri, J. , Nath, M. , Bhatia, R. , & Glew, L. (2016). *Examining the evidence base for forest conservation interventions*. Evidence Gap Map Report 4. New Delhi: International Initiative for Impact Evaluation (3ie).

[cl21149-bib-0473] Puri, J. , & Rathinam, F. (2019). *Challenges in real‐world impact evaluations: Some learning on costs and timeliness*. IEU Working Paper No. 03. Songdo, South Korea: Green Climate Fund.

[cl21149-bib-0474] Rathinam, F. , Cardoz, P. , Siddiqui, Z. , & Gaarder, M. (2019a). *Transparency and accountability in the extractives sector: A synthesis of what works and what does not*. 3ie Working Paper 33. New Delhi: International Initiative for Impact Evaluation (3ie). 10.23846/WP0033

[cl21149-bib-0475] Rathinam, F. , Finetti, J. , Snilstveit, B. , Siddiqui, Z. , Chirgwin, H. , Appell, R. , Dickens, E. , & Gaarder, M. (2019b). *The effect of transparency and accountability interventions in the extractive sectors: an evidence gap map*. 3ie Evidence Gap Map Report 14. New Delhi: International Initiative for Impact Evaluation (3ie). 10.23846/EGM014

[cl21149-bib-0476] Sabet, S. M. , & Brown, A. N. (2018). Is impact evaluation still on the rise? The new trends in 2010–2015. Journal of Development Effectiveness, 10(3), 291–304. 10.1080/19439342.2018.1483414UN

[cl21149-bib-0477] Salganik, M. J. (2017). Bit by bit: Social research in the digital age. Princeton University Press.

[cl21149-bib-0478] Serajuddin, U. , Uematsu, H. , Wieser, C. , Yoshida, N. , & Dabalen, A.L. (2015). *Data deprivation: Another deprivation to end (English)*. Policy Research Working Paper no. WPS 7252. Washington DC: World Bank Group.

[cl21149-bib-0479] Snilstveit, B , Bhatia, R , Rankin , & Kand Leach, B (2017). *3ie evidence gap maps: A starting point for strategic evidence production and use*. 3ie Working Paper 28. New Delhi: International Initiative for Impact Evaluation (3ie).

[cl21149-bib-0480] UN DESA (2018). The sustainable development goals report 2018. New York: UN. 10.18356/7d014b41-en

[cl21149-bib-0481] UN . (2019). *Global indicator framework for the sustainable development goals and targets of the 2030 agenda for sustainable development*. https://bit.ly/3nJ3pTk

[cl21149-bib-0482] UN Global Pulse . (2012). *Big data for development: Challenges and opportunities*. https://bit.ly/3kUMLhK

[cl21149-bib-0483] UN Global Pulse . (2013). *Big data for development: A primer*. https://www.unglobalpulse.org/document/big-data-for-development-primer/

[cl21149-bib-0484] UN Global Pulse . (2016). *Integrating big data into the monitoring and evaluation of development programmes*. https://beta.unglobalpulse.org/wp-content/uploads/2016/12/integratingbigdataintomedpwebungp-161213223139.pdf

[cl21149-bib-0485] UN Statistical Commission . (2014). Big data and modernization of statistical systems—Report of the Secretary General. UN Economic Social Council March.

[cl21149-bib-0486] Vaitla, B. (2014). The landscape of big data for development: Key actors and major research themes. UN Foundation/Data2X.

[cl21149-bib-0487] Van der Windt, P. , & Humphreys, M. (2016). Crowdseeding in Eastern Congo: Using cell phones to collect conflict events data in real time. Journal of Conflict Resolution, 60(4), 748–781.

[cl21149-bib-0488] Wassenich, P. (2007). *Data for impact evaluation (English)*. Doing Impact Evaluation Series no. 6. Washington DC: World Bank. http://documents.worldbank.org/curated/en/332891468313760995/Data-for-impact-evaluation

[cl21149-bib-0489] Webster, J. , Landegger, J. , Bruce, J. , Malunda, D. , Chantler, T. , Kumakech, E. , Schmucker, L. , Kiapi, L. , Kozuki, N. , Olorunsaiye, C. , & Byrne, E. (2019). *Impacts of IRC's Fifth Child community engagement strategy to increase immunisation in northern Uganda*. 3ie Grantee Final Report. New Delhi: International Initiative for Impact Evaluation (3ie).

[cl21149-bib-0490] Wilson, R. , zu Erbach‐Schoenberg, E. , Albert, M. , Power, D. , Tudge, S. , Gonzalez, M. , & Pitonakova, L. (2016). Rapid and near real‐time assessments of population displacement using mobile phone data following disasters: The 2015 Nepal earthquake. PLOS Currents, 8. 10.1371/currents.dis.d073fbece328e4c39087bc086d694b5c PMC4779046 26981327

[cl21149-bib-0492] Xu, W. , Li, Z. , Cheng, C. , & Zheng, T. (2013). Data mining for unemployment rate prediction using search engine query data. SOCA, 7, 33–42. 10.1007/s11761-012-0122-2

[cl21149-bib-0493] York, P. , & Bamberger, M. (2020). *Measuring results and impact in the age of big data: The nexus of evaluation, analytics, and digital technology*. The Rockefeller Foundation. https://www.rockefellerfoundation.org/wp-content/uploads/Measuring-results-and-impact-in-the-age-of-big-data-by-York-and-Bamberger-March-2020.pdf

